# One Atom Can Make All the Difference: Gas-Induced Phase Transformations in Bisimidazole-Linked Diamondoid Coordination Networks

**DOI:** 10.1021/jacs.3c01113

**Published:** 2023-04-26

**Authors:** Kyriaki Koupepidou, Varvara I. Nikolayenko, Debobroto Sensharma, Andrey A. Bezrukov, Matthias Vandichel, Sousa Javan Nikkhah, Dominic C. Castell, Kolade A. Oyekan, Naveen Kumar, Aizhamal Subanbekova, William G. Vandenberghe, Kui Tan, Leonard J. Barbour, Michael J. Zaworotko

**Affiliations:** † Bernal Institute, Department of Chemical Sciences, University of Limerick, Limerick V94 T9PX, Republic of Ireland; ‡ Advanced Materials and Bioengineering Research (AMBER) Centre, Dublin D02 R590, Republic of Ireland; § Department of Materials Science and Engineering, University of Texas at Dallas, Richardson, Texas 75080, United States; ∥ Department of Chemistry and Polymer Science, University of Stellenbosch, Matieland 7602, South Africa

## Abstract

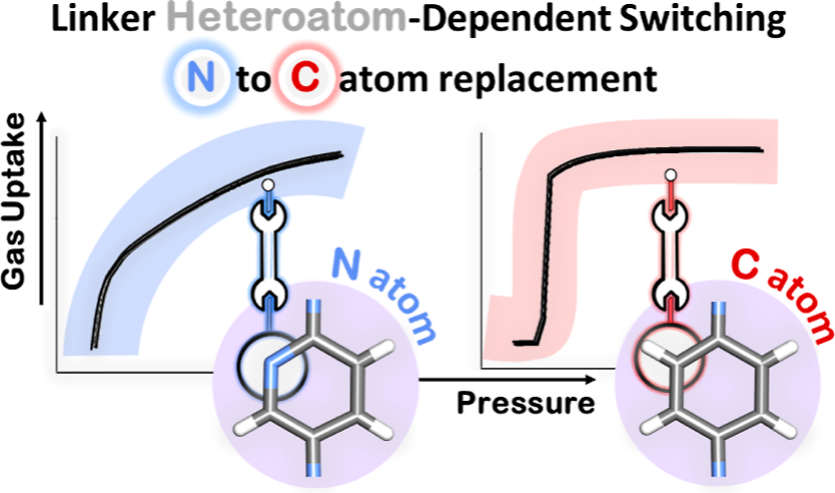

Coordination networks (CNs) that undergo gas-induced transformation from closed (nonporous) to open (porous) structures are of potential utility in gas storage applications, but their development is hindered by limited control over their switching mechanisms and pressures. In this work, we report two CNs, [Co(bimpy)(bdc)]_*n*_ (**X-dia-4-Co**) and [Co(bimbz)(bdc)]_*n*_ (**X-dia-5-Co**) (H_2_bdc = 1,4-benzendicarboxylic acid; bimpy = 2,5-bis(1H-imidazole-1-yl)pyridine; bimbz = 1,4-bis(1H-imidazole-1-yl)benzene), that both undergo transformation from closed to isostructural open phases involving at least a 27% increase in cell volume. Although **X-dia-4-Co** and **X-dia-5-Co** only differ from one another by one atom in their *N*-donor linkers (bimpy = pyridine, and bimbz = benzene), this results in different pore chemistry and switching mechanisms. Specifically, **X-dia-4-Co** exhibited a gradual phase transformation with a steady increase in the uptake when exposed to CO_2_, whereas **X-dia-5-Co** exhibited a sharp step (type F-IV isotherm) at *P*/*P*_0_ 0.008 or *P* 3 bar (195 or 298 K, respectively). Single-crystal X-ray diffraction, *in situ* powder XRD, *in situ* IR, and modeling (density functional theory calculations, and canonical Monte Carlo simulations) studies provide insights into the nature of the switching mechanisms and enable attribution of pronounced differences in sorption properties to the changed pore chemistry.

## Introduction

The design and performance fine-tuning of sorbents for gas storage technologies for gaseous energy sources^1,2^ remain a challenge. In the context of gas separations, a new generation of ultramicroporous physisorbents offers advantages over chemisorbents due to lower energy requirements for sorbent recycling.^3−8^ For gas storage using physisorbents, flexible metal–organic materials (FMOMs),^9^ or soft porous crystals,^10−13^ have emerged as attractive alternatives to rigid sorbents for storage applications since they can offer enhanced working capacity.^14,15^ FMOMs have been developed in the past two decades, and their sorption profiles tend to be different from rigid coordination networks (CNs),^9,13,16,17^ with isotherm types that occur because of dynamic behavior. These FMOMs can respond to external stimuli such as light,^18^ temperature,^19^ mechanical pressure,^20^ and guest molecules,^21^ making them of interest for gas separation,^22^ catalysis,^23^ drug delivery,^24^ and sensing^25^ in addition to gas storage.

“Switching” CNs belong to a subset of FMOMs^26^ that meet certain criteria, *i.e.*, they reversibly transform between nonporous (closed) and porous (open) phases. Such structural transformations are enabled by “breathing”^11^ or “gate-opening”,^22,27^*i.e.*, a gradual or a sharp increase in the uptake, respectively, triggered by a threshold pressure. This behavior has been classified as a type F-IV isotherm^9^ and is desirable for gas storage applications, due to the increased working capacity compared to type I sorbents.^28^ Nevertheless, despite their potential technological utility and the >100,000 crystal structures of metal–organic frameworks (MOFs) archived in the CSD,^29^ switching CNs remain relatively understudied. Indeed, our recent survey enumerated just 60 examples that are confirmed to switch from closed to open phases.^26^ Even when switching has been reported, the associated large structural transformations can impact crystal quality, and so, the crystal structures of closed phases are rarely reported. The dearth of structural information hinders systematic analysis of breathing phenomena, which is needed to elucidate crystal engineering principles for the next generation of switching sorbents.^30^

The inherent modularity of most CNs enables tailoring of their properties through substitution of framework components (nodes and linkers). Although the relationship between structure and sorption properties in CNs is complex, some factors that can affect CN gas/vapor sorption profiles have been identified. Metal cation substitution^31−37^ revealed that the metal can influence the threshold pressure of phase transitions and, in some cases, can induce a change of isotherm type. Since flexibility in FMOMs often arises from motion of the organic linker, ligand modification may also affect sorption profiles. Ligand functionalization has been shown to transform isotherm types from rigid to flexible by addition of flexible pendant groups on the framework,^38^ or by substituting hydrogen atoms with halogen atoms^39^ or other functional groups.^40,41^ Additionally, halogen atom substitution^42^ or addition of functional groups^43^ on the linker has been observed to alter the gate-opening pressure values. Ligand extension^44^ has been used to modify isotherm types, and ligand substitution^45^ has been reported to shift gate-opening pressure values depending on the rotational freedom of the ligand. Apart from composition, sorption profiles can also be affected by the crystal size,^46−50^ crystal defects,^51−53^ repeated cycling,^54^ sample pretreatment,^55−57^ and temperature.^58^

Owing to the limited number of switching CNs and systematic experimental and computational analysis of the factors that affect switching, design principles for switching sorbents remain in their infancy, highlighting the need for further insights into switching phenomena. To the best of our knowledge, while there are a handful of examples where functionalizing a linker with a halogen atom as a substituent can affect the sorption profiles,^39,42^ only one example where the replacement of a single atom on the linker core afforded a change in isotherm type is known.^59^ However, this example did not involve switching between closed and open phases. Here, we demonstrate for the first time that substitution of an N atom by a CH moiety results in a profound change in switching behavior through the study of two CNs, [Co(bdc)(bimpy)]_*n*_ (**X-dia-4-Co**) and **[Co(bdc)(bimbz)]**_***n***_ (**X-dia-5-Co**) (H_2_bdc = 1,4-benzendicarboxylic acid; bimpy = 2,5-bis(1*H*-imidazole-1-yl)pyridine; bimbz = 1,4-bis(1*H*-imidazole-1-yl)benzene), with isostructural open phases.

## Results and Discussion

**Figure 1:**
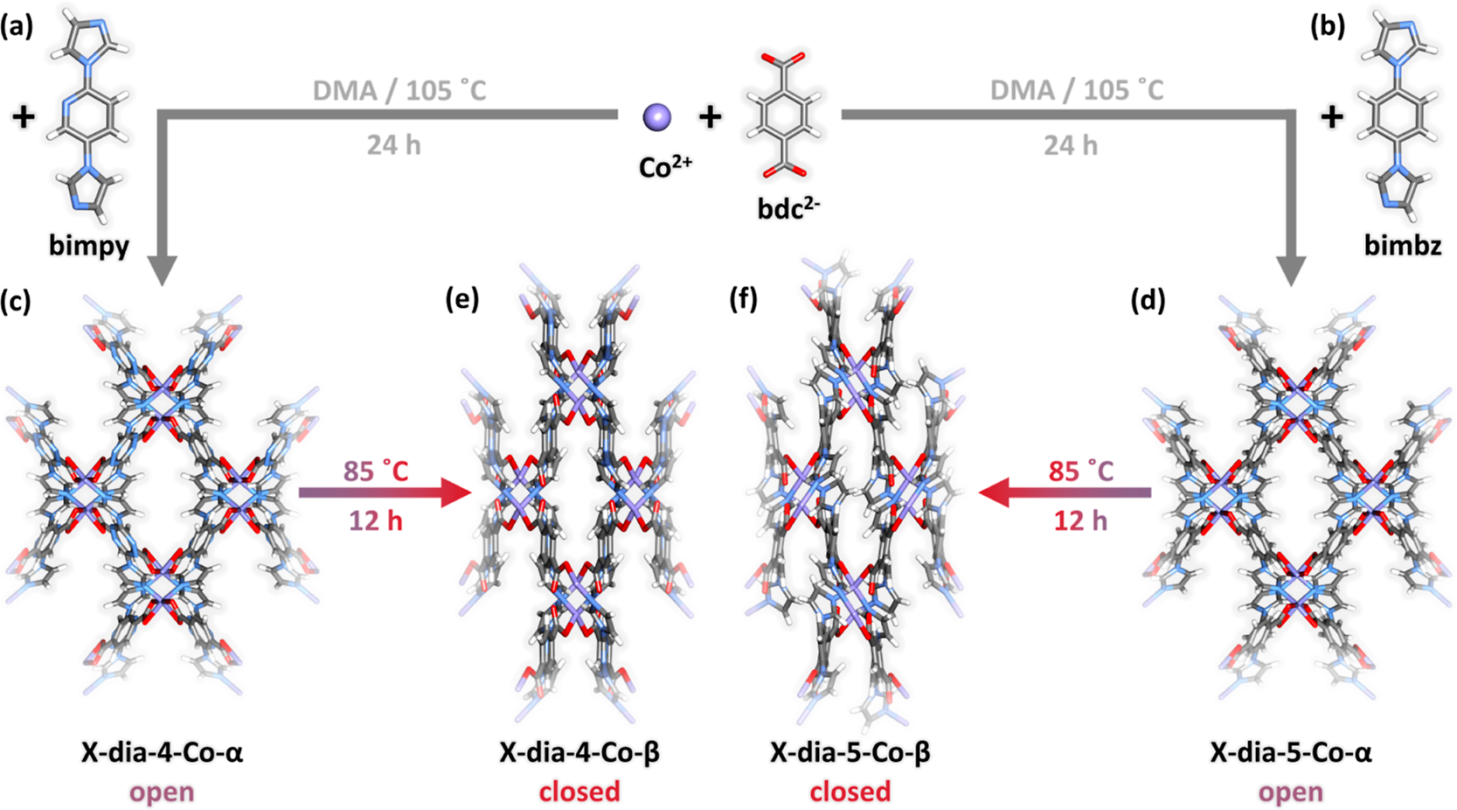
Schematic representation of phase transformations in **X-dia-4-Co** and **X-dia-5-Co**. (a) Bimpy linker and (b) bimbz linker. Crystal packing in (c) **X-dia-4-Co-α**, (d) **X-dia-5-Co-α**, (e) **X-dia-4-Co-β**, and (f) **X-dia-5-Co-β**. DMA = *N,N*-Dimethylacetamide. Color codes: N, blue; Co, purple; H, white; C, gray; and O, red.

The linkers selected herein, bimpy and bimbz, differ by just one atom in their core (Figure 1) and were studied because bisimidazole linkers are known to induce framework flexibility through conformational freedom,^60−63^ rather than contortion (strain) in pyridyl or dipyridyl linkers.^9^ A CSD search (version 2022.1.0) revealed six entries of CNs sustained by bimpy (Figure 1a), including an example with Co^2+^ nodes and **bdc^2–^** linkers.^64^ In contrast, bimbz (Figure 1b) has been used in >500 CNs. While there are no examples of FMOMs based on bimpy, FMOMs based on bimbz exist.^62^ No previous studies have compared how these two linkers impact sorption properties, the matter we address herein.

### Structural Analysis

Crystals of **X-dia-4-Co-α** and **X-dia-5-Co-α** suitable for single-crystal X-ray diffraction (SCXRD) analysis were obtained solvothermally (see Section S1 for experimental details). **X-dia-4-Co-α** and **X-dia-5-Co-α** crystallized in the orthorhombic space group *Pnna*. The asymmetric unit (Figure S1) comprises half a Co^2+^ ion, half a bdc^2–^ ligand, and a bimpy or bimbz linker, for **X-dia-4-Co-α** and **X-dia-5-Co-α**, respectively. The bimpy or bimbz linkers are positionally disordered over two general positions of equal occupancy which are related to each other through a crystallographic center of inversion (Figure S2). Additionally, the bimpy linker in **X-dia-4-Co-α** shows substitutional disorder of the nitrogen atom in the central ring over four positions (Figures S3–S5, see Section S2 for refinement details). Despite positional and substitutional disorder in the structures, all calculations regarding guest-accessible volume (probe radius 1.2 Å) were taken as an average of all possible configurations in each case.

Topological analysis revealed that both CNs exhibit diamondoid or dia network topology, with slight differences in cell parameters (Table S1), and are classified as IIIa dia nets^65^ with two pairs of two-fold interpenetrated dia nets that generate an overall four-fold interpenetrated dia framework (Figures S6 and S7). Each pair of nets has internetwork Co···Co distances of 11.611 and 11.698 Å, while the shortest Co···Co distance between adjacent pairs of nets is 8.138 and 7.970 Å, for **X-dia-4-Co-α** and **X-dia-5-Co-α**, respectively. Despite four-fold interpenetration, the CNs exhibit rectangular channels along the *b*-axis (Figure 1c,d) with a guest accessible void volume of *ca.* 28.7% for both compounds. Thermogravimetric (TG) analysis (Figures S8 and S9) showed an initial solvent loss of 15.0 and 16.0 wt % from 50 to 170 °C for **X-dia-4-Co-α** and **X-dia-5-Co-α**, respectively, corresponding to loss of one *N,N*-dimethylacetamide (DMA) guest molecule per Co atom. Both compounds were observed to be stable up to 380 °C. Powder X-ray diffraction (PXRD) patterns (Figure S10) demonstrated bulk phase purity of both as-synthesized compounds.

### Open to Closed Phase Transformations

Heating the as-synthesized **α** phases induced structural transformations to the respective closed phases **X-dia-4-Co-β** (Figure 1e) and **X-dia-5-Co-β** (Figure 1f). Despite the fact that the two closed phases arise from isostructural open phases, they are different in terms of space group, guest accessible space, and linker conformations. The differences in atomic positions resulted in distinctive PXRD patterns (Figure S11), which also validate phase purity of the closed phases.

**Figure 2:**
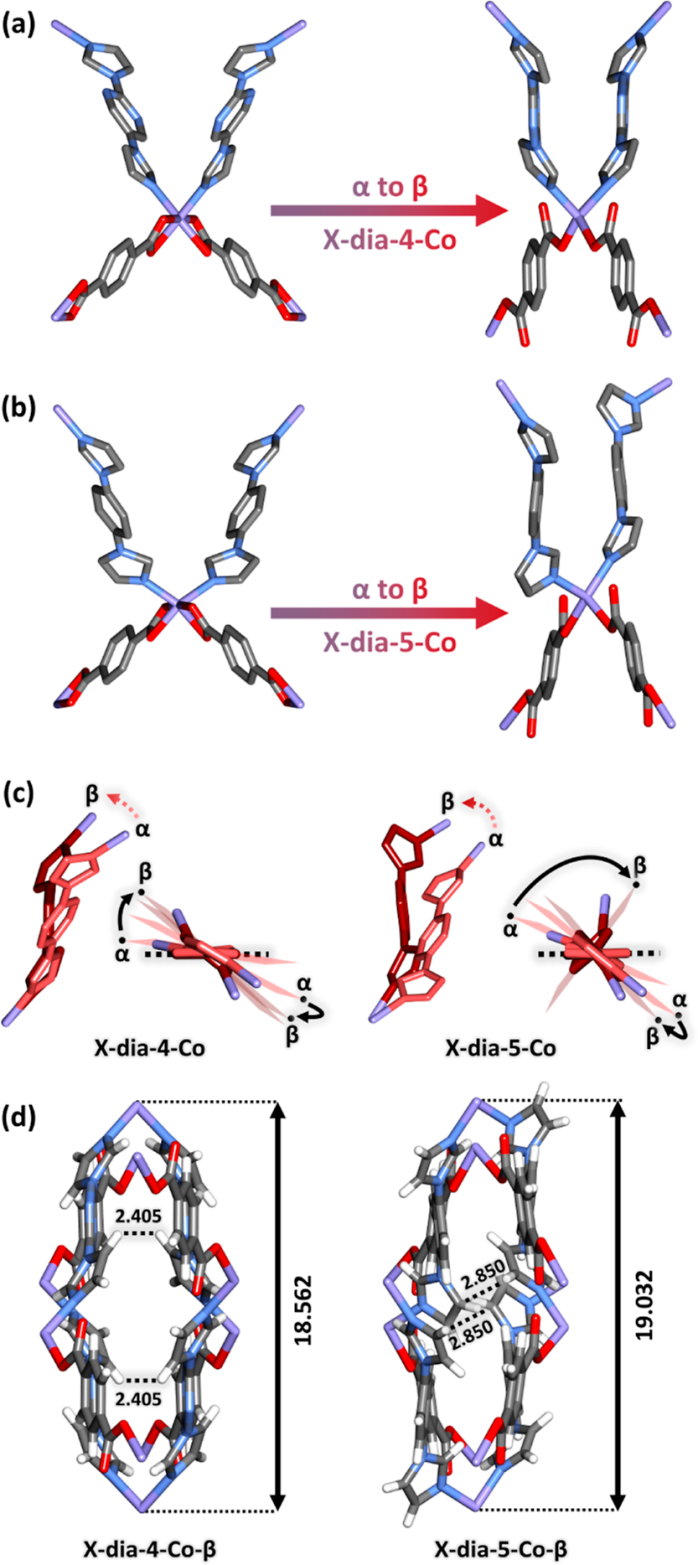
Structural transformations: (a) from **X-dia-4-Co-α** to **X-dia-4-Co-β** and (b) **X-dia-5-Co-α** to **X-dia-5-Co-β**. (c) Linker motions during the transformation from the α phase (light red) to the **β** phase (dark red) in **X-dia-4-Co** and **X-dia-5-Co**. (d) Crystal packing with close contact distances (CH···HC and CH···C interactions) and Co···Co distances noted in Å in **X-dia-4-Co-β** and **X-dia-5-Co-β**. Color codes: N, blue; Co, purple; H, white; C, gray; and O, red.

Although the **X-dia-4-Co-α** to **X-dia-4-Co-β** transformation was not accompanied by a change in the space group (both remained as *Pnna*), the as-synthesized phase underwent a 21.4% reduction in unit cell volume. SCXRD analysis of the two phases revealed that the transformation was enabled by motion of the bimpy and bdc^2–^ linkers (Figures 2a, S12, and S13). In **X-dia-4-Co**, rotation and bending of the imidazole rings with respect to the central ring (Figures 2c and S12) resulted in longer intranetwork Co···Co distances. In addition, the bdc^2–^ coordination mode changed from chelating to monodentate, promoting a larger intranetwork Co···Co distance in **X-dia-4-Co-β** vs **X-dia-4-Co-α** (Figure S13). As a result, each adamantoid cage was elongated along its diagonal from 31.279 to 36.062 Å (Figure S14) and underwent changes in its Co–Co–Co angle (Figure S15). Like **X-dia-4-Co-α**, **X-dia-4-Co-β** exhibited four-fold interpenetration. The phase change led to the individual nets moving further apart from each other (Figures S4 and S5) and altered intranetwork Co···Co distances and angles (Figure S16), causing a reduction in guest accessible space from *ca.* 28.7 to *ca.* 7.3%.

In the case of **X-dia-5-Co-β**, activation induced a change in space group symmetry from *Pnna* to *Pna*2_1_. The transformation to a non-centrosymmetric space group arose from the loss of the center of inversion in the bimbz linker (Figure 2b). The non-centrosymmetric character of the space group is also apparent in differences in the coordination environment around the metal center (Figure S17, Tables S2 and S3). The bulk phase purity was also tested by Pawley profile fitting, which demonstrated good agreement between calculated and experimental PXRD patterns (Figure S18 and Table S4). Similar bending motions of the linkers (Figures 2b,c, S19, and S20) as seen in **X-dia-4-Co-β** and subnetwork displacement (Figures S6 and S7) enabled transformation to a closed phase with negligible guest accessible space (0%).

Structural analysis of the two closed phases revealed that **X-dia-5-Co-β** contracted even more than **X-dia-4-Co-β** due to further twisting of the imidazole rings (Figure 2c). In **X-dia-4-Co-β**, a close contact distance between two hydrogen atoms from two facing imidazole rings in opposite pore walls precluded further contraction of the structure (Figure 2d). Variable-temperature PXRD studies under N_2_ (Figures S21 and S22) revealed that both open phases converted to the corresponding closed phases from 70 to 90 °C and that the closed phases were maintained after cooling to room temperature. These results are also in agreement with differential scanning calorimetry (DSC) analysis, which showed an exothermic peak for the first heating cycle of **X-dia-4-Co-α** and **X-dia-5-Co-α** but not for the second cycle (Figures S23 and S24). TG analysis (Figures S8 and S9) confirmed that both phases are guest-free despite the existence of *ca.* 142.43 Å^3^ of solvent accessible space in **X-dia-4-Co-β**.

### Gas Sorption Studies

**Figure 3:**
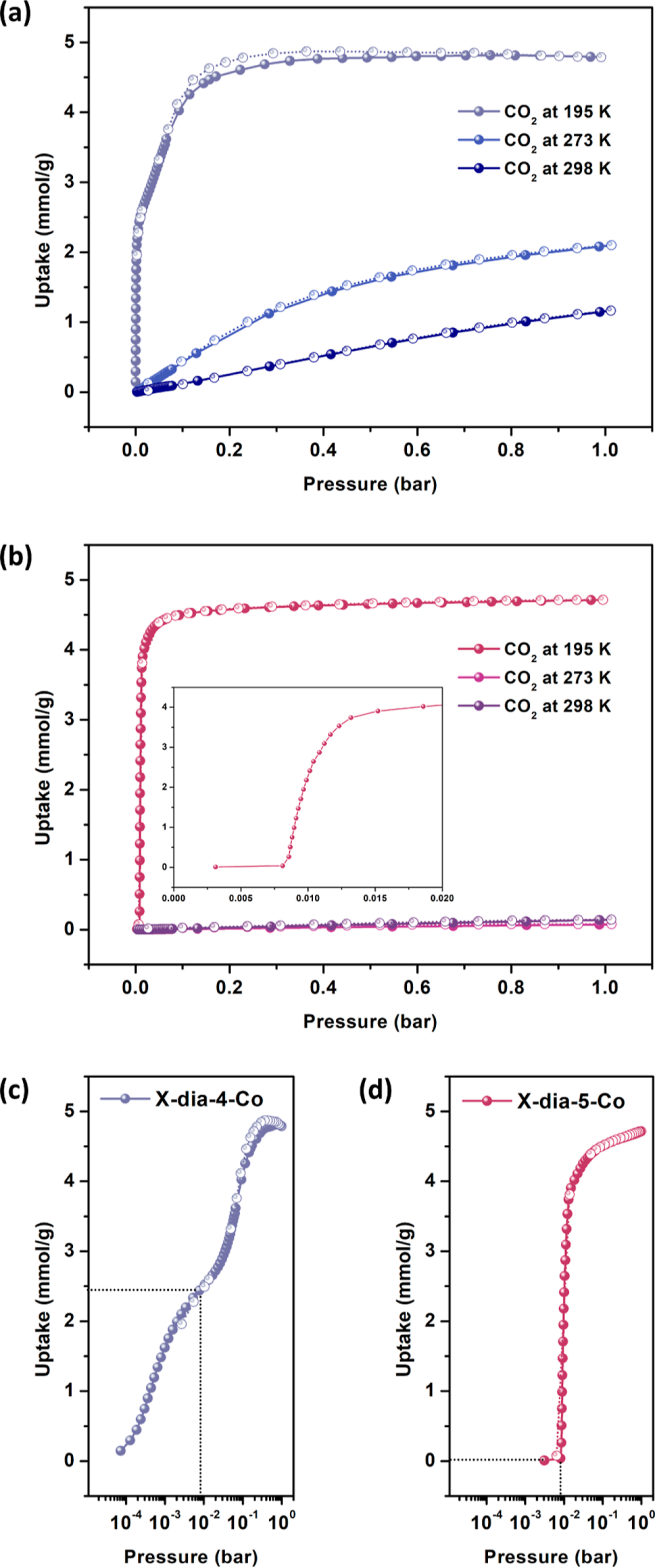
Low-pressure CO_2_ sorption isotherms collected at 195, 273, and 298 K for (a) **X-dia-4-Co-β** and (b) **X-dia-5-Co-β**. Log plots of the isotherms collected at 195 K for (c) **X-dia-4-Co** (blue) and (d) **X-dia-5-Co** (red). Black dotted lines show the difference in uptakes at *P* 0.01 bar.

The structural differences between the two closed phases, **X-dia-4-Co-β** and **X-dia-5-Co-β** (Figure 2d), as well as their different pore chemistry, prompted us to investigate if their sorption behavior might be different. Low-pressure CO_2_ sorption isotherms collected at 195 K revealed that **X-dia-4-Co-β** steadily adsorbed CO_2_ with increasing pressure in a manner resembling a type I isotherm, as exhibited by most rigid sorbents (Figure 3a). The saturation uptake of 4.79 mmol/g (or 107.7 cm^3^/g) at *P*/*P*_0_ = 1 and minor deviations from ideal Langmuir-type behavior were evident. Conversely, **X-dia-5-Co-β** exhibited a sharp step indicative of switching behavior under the same conditions (Figure 3b). The resulting single-step type F-IV isotherm had a gate-opening event at *P*/*P*_0_ 0.008 and a saturation uptake of 4.71 mmol/g (or 102.5 cm^3^/g) at *P*/*P*_0_ = 1. The calculated single-point pore volumes^66^ of 0.164 and 0.160 cm^3^/g agree with the calculated values from the crystal structures of **X-dia-4-Co-α** (0.165 cm^3^/g) and **X-dia-5-Co-α** (0.167 cm^3^/g), respectively. The Langmuir surface areas for the open phases were determined to be 499.6 and 482.5 m^2^/g for **X-dia-4-Co** and **X-dia-5-Co**, respectively (Table S5). Low-pressure cycling experiments (Figure S25) demonstrated both repeatability of the isotherms and particle size independence. CO_2_ gas sorption studies at higher temperatures (273 and 298 K) confirmed the significant difference in properties; **X-dia-4-Co** showed appreciable adsorption at low pressures with an uptake of 2.10 mmol/g at 273 K, whereas **X-dia-5-Co** afforded negligible uptake (Figure 3). N_2_ gas sorption studies at 77 K revealed that neither material showed appreciable uptake (Figure S26), while gas sorption studies for C2 gases (C_2_H_2_, C_2_H_4_, and C_2_H_6_) at 298 K confirmed similar behavior to the one shown for CO_2_ (Figure S27), *i.e.*, **X-dia-4-Co** displaying a smeared isotherm profile for all three gases, **X-dia-5-Co** showing steep gate-opening for C_2_H_2_ and negligible uptake for C_2_H_4_ and C_2_H_6_.

**Figure 4:**
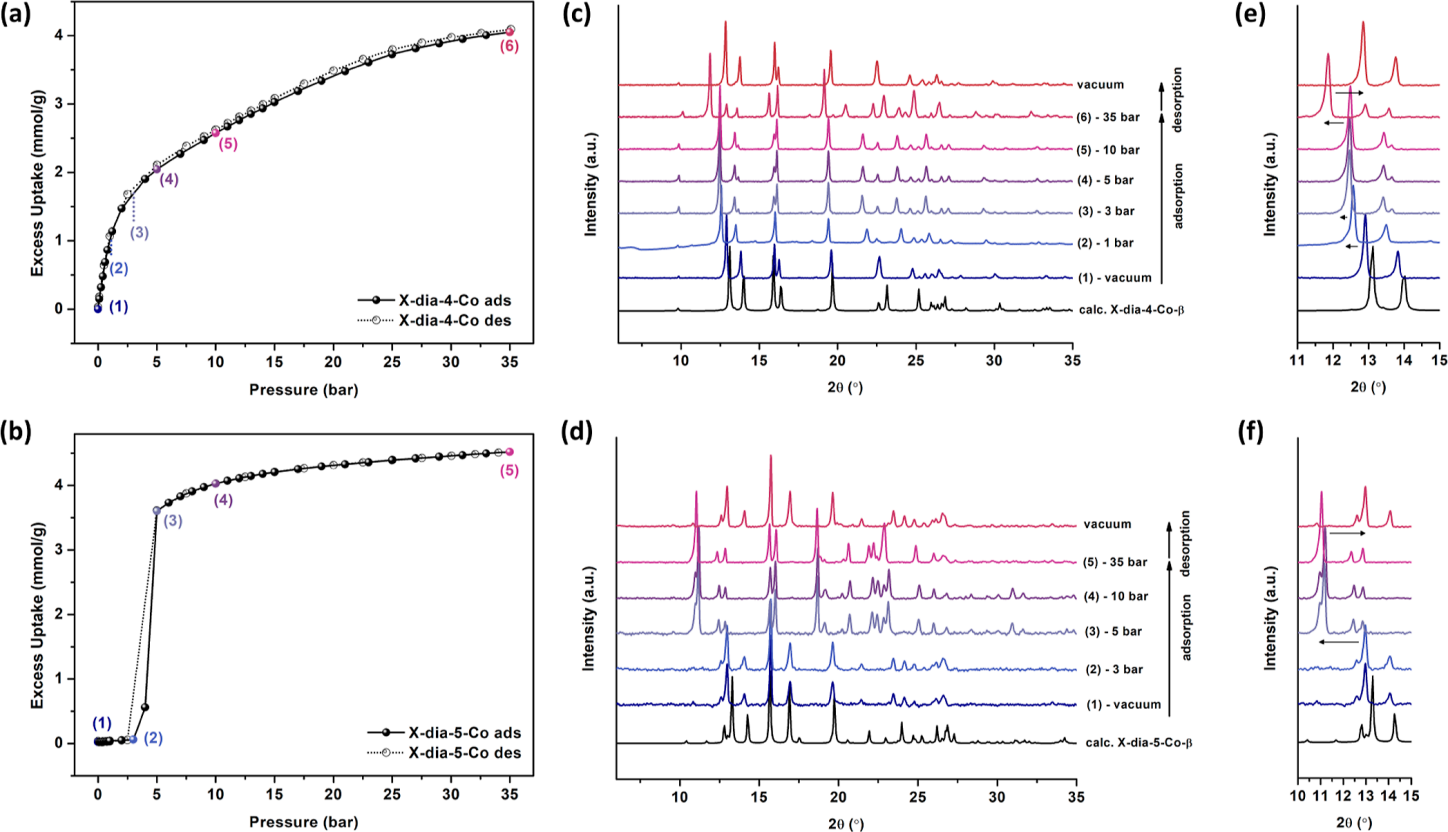
High-pressure CO_2_ sorption isotherm for (a) **X-dia-4-Co-β** and (b) **X-dia-5-Co-β** at 298 K. Selected corresponding *in situ* CO_2_-loaded PXRD patterns for (c) **X-dia-4-Co-β** and (d) **X-dia-5-Co-β** at different adsorption (ads; closed sphere) and desorption (des; open sphere) points. Magnified PXRD patterns from 10 to 15° 2θ values for (e) **X-dia-4-Co-β** and (f) **X-dia-5-Co-β**.

High-pressure CO_2_ sorption isotherms collected at 298 K (Figure 4) were consistent with the low-pressure isotherms. **X-dia-4-Co** exhibited steady uptake of CO_2_ with increasing pressure to an uptake of 4.05 mmol/g at 35 bar. Similar behavior was observed toward CO_2_ at 273 K or CH_4_ at 298 K (Figure S28). **X-dia-5-Co** showed a single-step type F-IV isotherm with gate-opening at 3 bar and an uptake of 4.52 mmol/g at 35 bar. This gate-opening pressure shifted to 1 bar at 273 K (Figure S29). Cycling experiments revealed that the CO_2_-induced switching is reversible, the sorption capacity of **X-dia-5-Co** being retained after eight cycles. The switching behavior of **X-dia-5-Co** is also apparent for other gases including CH_4_ (Figure S30), which exhibited a type F-IV isotherm at 298 K with gate-opening at 15 bar and uptakes of 2.57 mmol/g at 35 bar and 2.92 mmol/g at 65 bar. This uptake is too low for practical utility of **X-dia-5-Co** for methane storage. Nevertheless, this is a rare example of a switching CH_4_ sorbent with the gate-opening pressure value in the desirable pressure range (between 5 and 35 bar) for ANG technologies.^9,14^

*In situ* variable-pressure PXRD studies of the CO_2_-loaded materials provided insights into the phase transitions associated with gas sorption. The loading of **X-dia-4-Co-β** is consistent with a two-step mechanism (Figures 3c, 4c,e). The first step involved flexibility with a gradual phase transition from the closed phase (vacuum) to a CO_2_-loaded phase (3 bar). The flexibility was confirmed by shifting of the peak positions upon adsorbing CO_2_ in the low-pressure region (0–1 bar CO_2_, Figure S31). Above 3 bar, the material continued to adsorb CO_2_ while remaining in this intermediate phase without significant unit cell change, as shown by the *in situ* PXRD patterns collected at 3, 5, and 10 bar. The second step involved another phase transition from the intermediate phase (10 bar) to the fully loaded phase (35 bar). In contrast, the sharp uptake of **X-dia-5-Co-β** can be attributed to an abrupt phase transformation (Figures 3d, 4d,f) from the closed phase (vacuum) to the CO_2_-loaded phase (35 bar). Both materials return to their respective closed phases after desorption. Even though the two CO_2_-loaded phases at 35 bar are not identical to the as-synthesized open phases **X-dia-4-Co-α** and **X-dia-5-Co-α** (Figure S32), Pawley profile fits for the two materials provided structural insights (Figures S33 and S44 and Tables S6 and S7). Fitting of the in situ PXRD patterns of **X-dia-4-Co** at 3, 5, and 10 bar confirmed the existence of an intermediate phase with a cell volume of *ca.* 2055 Å^3^ (Table S6), while **X-dia-5-Co** demonstrated an open phase for the CO_2_-loaded structure with a cell volume of *ca.* 2300 Å^3^ (Table S7).

Such a significant alteration of isotherm types induced by a single heteroatom substitution is intriguing. It was previously shown that on already established switching platforms, crystals smaller than a critical diameter can retain their open state, showing a type I isotherm.^67^ However, the particle size distributions for **X-dia-4-Co-β** and **X-dia-5-Co-β** are equivalent to each other (Figures S45–S49), eliminating particle size as being responsible for the different isotherm profiles. SEM images after CO_2_ sorption indicated that the crystal size and quality had diminished after repeated cycling (Figure S50); however, we observed reproducible isotherms over five cycles without an appreciable change in inflection points (Figure S25). We then focused on framework components. Given that the bound bdc^2–^ linker behaved consistently during phase transformations (Figures S7 and S9), we shifted our attention to the other organic linker ligand. SCXRD revealed that the difference in isotherm shapes could be attributed to differences in the two closed phases arising from the chemical composition of the **bimpy** and **bimbz** linkers, which in turn results in distinct mechanisms for phase transformation. The denser packing of **X-dia-5-Co-β** vs **X-dia-4-Co-β** (Figure 2d) could be attributed to observation of four close contacts in **5** (CH···O, 2.642 and 2.675 Å; CH···C, 2.850 and 2.886 Å) *vs.* one close contact in **4** (CH···O, 2.642 Å), all involving CH hydrogen atoms from imidazole moieties (Figure S51). These additional contacts may be responsible for a higher barrier for transformation to the open phase in **5**, causing it to remain closed below the threshold pressure. Therefore, it is possible to assert that the transformation from closed to open is gradual in **X-dia-4-Co** but sharp in **X-dia-5-Co** due to the nature of the bisimidazole linkers. As previously discussed,^52^ it is also possible that the substitutional disorder in **X-dia-4-Co** has an impact on flexibility.

### *In Situ* Infrared Spectroscopy

**Figure 5:**
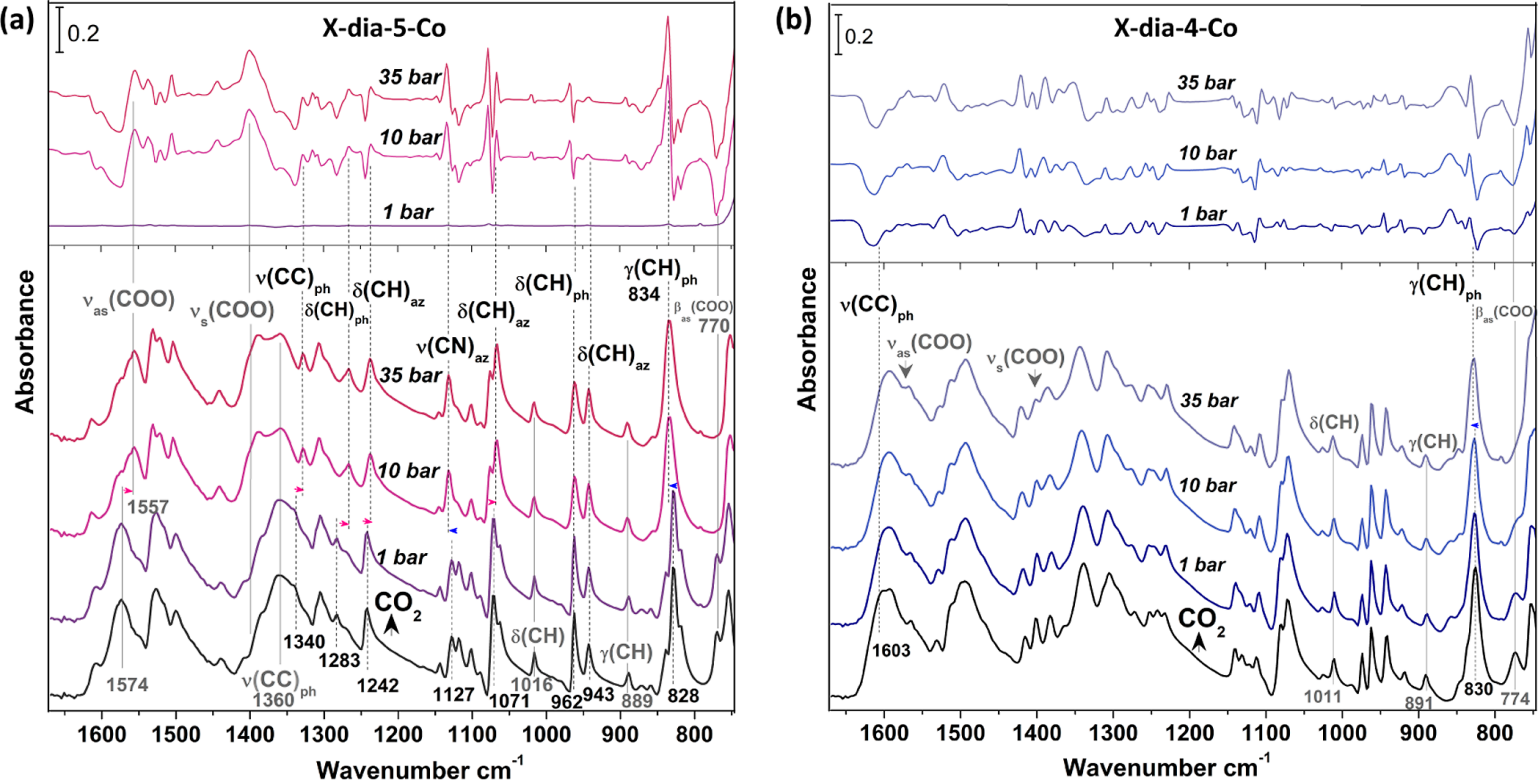
IR spectra of (a) **X-dia-5-Co** and (b) **X-dia-4-Co** upon loading of CO_2_ as a function of pressure. Bottom panel: adsorption spectra of the samples referenced to blank KBr pellet under vacuum. Top panel: difference spectra obtained by subtracting the spectrum of the activated sample (black line) from that of CO_2_-loaded at varying pressures to show the changes after CO_2_ adsorption. Vibrational modes of bimbz/bimpy and bdc^2–^ are labeled by black dashed lines and gray solid lines, respectively. Notations and acronyms: ν, stretch; δ, in plane deformation; γ, out of plane deformation; β, bend; ph, phenyl; az, azole; s, symmetric; and as, asymmetric.

To unveil mechanistic insights into CO_2_ binding and the structural transformations of **X-dia-4-Co** and **X-dia-5-Co**, *in situ* infrared spectroscopy measurements^68−71^ were conducted on the samples upon increasing loading of CO_2_ (see Section S10 for experimental details). The IR spectra of activated **X-dia-4-Co-β** and **X-dia-5-Co-β** (Figure 5) are dominated by bands associated with the organic linkers bdc^2–^, bimpy, and bimbz, which are summarized in Table S8. Bands were identified by inspecting the spectra of the free linkers (Figure S52) and by calculating their vibrational modes using density functional theory (DFT) calculations (Figure S53). The comparative analysis of bimpy and bimbz showed that bimpy displays a series of new bands that are not present in bimbz, due to the nitrogen atom of the central pyridyl ring, which not only produces new vibrations (Figure S54) but also breaks the molecular symmetry, thus making some modes IR-active.

The phonon modes of **X-dia-4-Co-β** and **X-dia-5-Co-β** were analyzed upon loading of CO_2_ as a function of pressure since the signal of the gas phase CO_2_ was prohibitively high, making it impossible to directly observe the adsorbed CO_2_ (Figure S55). As shown in Figure 5a, loading of CO_2_ at 1 bar did not trigger noticeable changes to **X-dia-5-Co**. Pronounced changes occurred by increasing CO_2_ pressure to ∼10 bar (see Table S8). Regarding the bdc^2–^ linker, the following changes were observed: (i) ν_as_(COO^–^) mode at 1574 cm^–1^ downward (red-) shifted to 1557 cm^–1^, which is consistent with the elongation and/or softening of carboxylate bonds as a result of structural expansion after adsorbing CO_2_; (ii) ν_s_(COO^–^) was enhanced in its intensity, which can be caused by the decrease of O–C–O angle and related increase of its total dipole moment. Regarding the bimbz linker, bands associated with the phenyl ring underwent the most notable changes: (i) γ(CH)_ph_ at 828 cm^–1^ upward (blue-) shifted to 834 cm^–1^; (ii) in plane modes δ(CH)_ph_ at 1283 cm^–1^ red-shifted to 1267 cm^–1^; and (iii) the two in plane deformation modes δ(CH)_az_ of the azole −CH group next to the phenyl ring at 1242 and 1071 cm^–1^ exhibited a clear red-shift. In contrast, the 943 cm^–1^ δ(CH)_az_ mode involving azole CH deformation near the Co–N bond (Figure S54g), as well as the δ(CH) and γ(CH) modes of bdc^2–^ linker at 1016 and 889 cm^–1^, respectively, were barely affected. Therefore, we infer that adsorbed CO_2_ interacts primarily with the bimbz linker, most likely in close proximity to the plane of the phenyl ring. Furthermore, the phenyl ring stretching mode ν(C=C) on the bimbz linker was red-shifted from 1340 to 1328 cm^–1^, suggesting elongation or softening of the C=C bonds. The azole ring ν(C–N) mode near to the Co–N bond was slightly blue-shifted by 5 to 1132 cm^–1^, indicating shortening and/or hardening of the C–N bonds (Figure S54d). Increasing the pressure to 35 bar did not cause further changes, indicating completion of the structural transformation at 10 bar.

In the case of **X-dia-4-Co**, noticeable changes to the phonon modes occurred after loading of CO_2_ at 1 bar and continued upon increasing pressure, as indicated by the difference spectra (Figure 5b). Analysis of these changes is more challenging than that of **X-dia-5-Co**, due to the appearance of more infrared-active modes from the bimpy linker (Figure S54) that overlap, *e.g.*, the position and shift of *v*_as, s_(COO^–^) modes were obscured by the pyridyl ring modes that occurred in the same region. Even so, we still observed that the distinct β_as_(COO-) mode at 774 cm^–1^ in pristine **X-dia-4-Co** gradually decreased upon loading CO_2_ from 1 to 10 bar, indicative of the onset of the structural transformation in this pressure region. At 35 bar, the original mode β_as_(COO^–^) at 832 cm^–1^ almost disappeared, which corresponds to the completion of structural transformation at this pressure value. Similar to the observation in **X-dia-5-Co**, the pyridyl ring mode γ(CH) at 830 cm^–1^ of the bimpy linker showed blue-shift (∼2 cm^–1^ at 35 bar) upon loading of CO_2_, while the δ(CH)/γ(CH) modes of the bdc^2–^ linker revealed only slight perturbation. Moreover, a decrease of the intensity of the mode around 1603 cm^–1^ that can be attributed to C–C stretching of the pyridyl ring on the bimpy linker (Figure S54i) was evident. These observations indicate not only that CO_2_ interacts with the pyridyl moiety of the bimpy linker but also that the behavior of **X-dia-4-Co** upon increasing pressure of CO_2_ is distinct to that of **X-dia-5-Co**.

### Computational Studies

**Figure 6:**
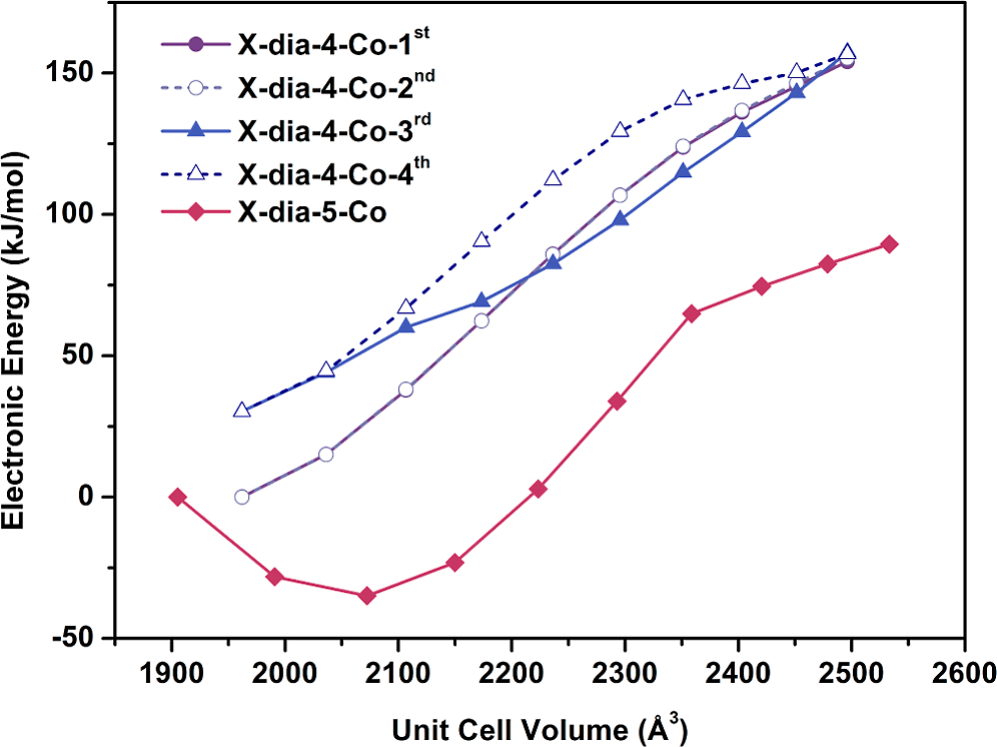
Optimized energy landscape (kJ/mol) of structural transitions between **β** and **α** phases as a function of unit cell volume (Å^3^) for **X-dia-4-Co-1^st^**, **X-dia-4-Co-2^nd^**, **X-dia-4-Co-3^rd^**, **X-dia-4-Co-4^th^**, and **X-dia-5-Co** determined via NEB calculations. Note that the plots for **X-dia-4-Co-1^st^** (full circles, solid line) and **X-dia-4-Co-2^nd^** (open circles, dashed line) almost completely overlap.

To further understand the effect of chemical composition on sorption properties, we performed DFT calculations and canonical Monte Carlo (CMC) simulations. For **X-dia-5-Co**, only one structure was considered when modeling CO_2_-binding sites since the linker bimbz is symmetrical. In contrast, four structures were considered for **X-dia-4-Co** because there are four possible positions for the nitrogen atom in the central pyridyl ring of the bimpy linker (**X-dia-4-Co-1^st^**, **X-dia-4-Co-2^nd^**, **X-dia-4-Co-3^rd^**, and **X-dia-4-Co-4^th^**, see Section S11 for computational details). The relative energies of the four optimized closed pore phases of **X-dia-4-Co-β** were determined to be 0.0, 0.0, 30.3, and 30.3 kJ/mol (Table S9). In the large pore phases of **X-dia-4-Co-α**, the relative energies of the four configurations were similar: 154.1, 155.3, 157.4, and 156.8 kJ/mol, respectively. While a large energy difference of more than 120 kJ/mol between the closed and open pore phases of **X-dia-4-Co** was calculated, interestingly, the energy difference between **X-dia-5-Co-β** and **X-dia-5-Co-α** was only 89.5 kJ/mol, which intuitively would point toward an easier opening of the structure (or easier breathing behavior) in the case of **X-dia-5-Co** compared to **X-dia-4-Co**. Yet, experimentally the opposite was observed: **X-dia-5-Co** required a higher adsorbate pressure to transform from the closed to the open phase (Figure 4). Therefore, the transitions between closed and open pore structures can be considered to unravel plausible intermediate structures and their relative energies, and such empty host energy profiles representing the energy of the framework as function of the volume can be useful for further studies.^72−75^ The closed (**β**) to open (**α**) empty host energy landscapes were calculated via nudged elastic band (NEB) calculations and demonstrated that the opening up of **X-dia-5-Co-β** resulted initially in more stable structures for cell volumes between 1900 and 2200 Å^3^ (Figure 6). This is mainly due to enhanced ligand–ligand stacking interactions at cell volumes around 2050 Å^3^. Therefore, it seems less likely for CO_2_ to occupy binding sites in **X-dia-5-Co** for cell volumes lower than 2200 Å^3^. Indeed, the calculated CO_2_ adsorption in a 2072.40 Å^3^ unit cell was determined to be exothermic by −2.2 kJ/mol but still endergonic by +36.8 kJ/mol (see Table S10 for adsorption enthalpies and Gibbs free energies). For the next structural image having a larger unit cell volume of 2149.89 Å^3^, the CO_2_ adsorption was found to be exothermic by −16.0 kJ/mol but still endergonic by +23.6 kJ/mol. Interestingly, the CO_2_ adsorption in an even larger cell volume of 2223.40 Å^3^ became even more exothermic (by −29.5 kJ/mol) but remained endergonic by +8.5 kJ/mol. Based on making a linear extrapolation of the Gibbs free energy differences that represent the spontaneity of a process, the adsorption process at 298 K is likely to start around or after an adsorbate pressure of 1 bar and around a unit cell volume of 2270 Å^3^, which agrees with our experimental observations (Figure S44 and Table S6). Indeed, for **X-dia-5-Co** with a unit cell volume of *V*_5_ = 2292.98 Å^3^, the CO_2_ adsorption enthalpy and Gibbs free energy were determined to be −37.3 and −0.3 kJ/mol, respectively.

CMC simulations at a fixed loading of four molecules per unit cell were performed on relevant structures of **X-dia-4-Co** and **X-dia-5-Co** (Figure S56, Tables S11 and S12). Interestingly, only two and three CO_2_-binding sites per unit cell could be identified for the **X-dia-5-Co** structures with unit cell volumes of 2072.40 and 2149.89 Å^3^, respectively, while starting from 2223.40 Å^3^, four binding sites were observed. The identified binding sites from CMC are visualized in Figures S57–S62. The CO_2_ coordinates of the most stable identified DFT-optimized binding site can be visualized together with the CO_2_-binding site isosurfaces derived from the CMC simulations, as shown in Figures S63–S67. This was carried out for frameworks with realistic unit cell volumes based on our experimental observations: **X-dia-4-Co** (*V*_1_ = 2036.27 Å^3^) and **X-dia-5-Co** (*V*_2_ = 2072.40 Å^3^, *V*_3_ = 2149.89 Å^3^, *V*_4_ = 2223.40 Å^3^, and *V*_5_ = 2292.98 Å^3^). For **X-dia-4-Co**, the DFT-optimized most stable CO_2_ configuration overlapped with one of the four CO_2_-binding site isosurfaces identified via CMC simulations (Figures S63–S66). In the case of **X-dia-5-Co**, no overlap was observed between most stable DFT-binding sites and CMC-binding isosurfaces; in contrast, less stable CO_2_-binding sites were identified upon optimization of CO_2_ (Figure S67). However, when placing a CO_2_ molecule in the middle of the identified CMC-binding site isosurface for **X-dia-5-Co** (*V*_5_ = 2292.98 Å^3^) followed by DFT optimization, a realistic CO_2_ adsorption enthalpy and Gibbs free energy of −35.2 and 3.2 kJ/mol, respectively, were found, indicating that this position is only slightly less favorable compared to the most stable binding site identified with DFT.

**Figure 7:**
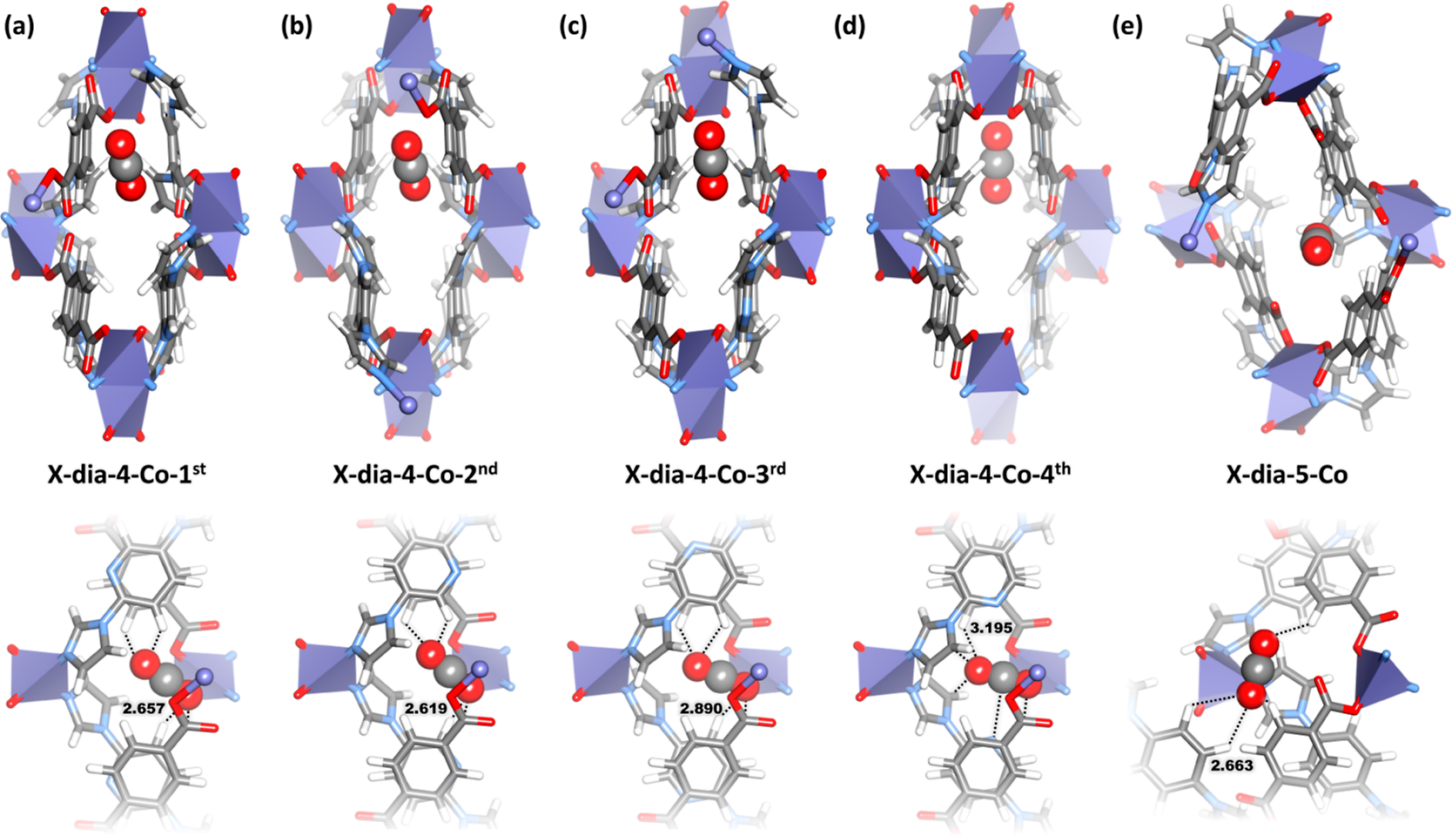
Binding sites of CO_2_ in **X-dia-4-Co** (*V*_1_ = 2036.27 Å^3^) (a–d) and **X-dia-5-Co** (*V*_5_ = 2292.98 Å^3^) (e); obtained with DFT calculations. Color codes: N, blue; Co, purple; H, white; C, gray; and O, red. Selected close contact distances are shown in black dotted lines, while the shortest close contact distance in each framework is listed (in Å).

The most stable DFT-optimized binding sites are shown in Figure 7. The calculated CO_2_-loaded frameworks are consistent with our experimental CO_2_-loaded PXRD patterns at 10 bar (Figures S68 and S69). The adsorbed CO_2_ molecules in the **X-dia-4-Co** (*V*_1_ = 2036.27 Å^3^) frameworks interact primarily with the central pyridyl ring of the **bimpy** linker, with the shortest  distances being 2.657, 2.619, and 2.890 Å for **X-dia-4-Co-1^st^**, **X-dia-4-Co-2^nd^**, and **X-dia-4-Co-3^rd^**, respectively, and the  distance being 3.465 Å in **X-dia-4-Co-4^th^** (see Table S13 for full list of interactions). This is in agreement with our *in situ* IR measurements (Figure 5). In the case of **X-dia-5-Co** (*V*_5_ = 2292.98 Å^3^), the predominant CO_2_ interactions were observed to involve the phenyl ring of the **bimbz** linker, as also validated by our *in situ* IR results, with two  distances of 2.663 and 2.885 Å. Mapping out the position of the adsorbed CO_2_ in **X-dia-5-Co** frameworks with intermediate pore openings (*V*_2_ = 2072.40 Å^3^, *V*_3_ = 2149.89 Å^3^, and *V*_4_ = 2223.40 Å^3^) revealed that CO_2_ molecules adopt a similar position regardless of the unit cell volume (Figure S70), with prominent  interactions (Table S13).

In order to further study the differences in sorption behavior of the two compounds, DSC sorption experiments were conducted on **X-dia-4-Co-β** and **X-dia-5-Co-β** (Figure S71, see Section S7 for experimental details). Analysis of the DSC profiles showed that while **X-dia-5-Co** displayed a single sharp exothermic peak upon adsorption of CO_2_ at 198 K, **X-dia-4-Co** showed an additional broad shoulder peak, confirming a two-step CO_2_ sorption mechanism and indicating that there is more than one possible binding site for CO_2_ in **X-dia-4-Co**. The presence of multiple binding sites in **X-dia-4-Co** could be the underlying reason of the experimental observation of a two-step phase transition, instead of steep gate-opening.

## Conclusions

We report two new CNs with isostructural as-synthesized (open) phases, **X-dia-4-Co** and **X-dia-5-Co**, and demonstrate that they undergo solvent and gas-induced phase transformations. While **X-dia-4-Co** showed steadily increasing CO_2_ uptake as a function of pressure, **X-dia-5-Co** exhibited closed to open switching under the same conditions. The phase changes were structurally characterized using SCXRD experiments and *in situ* PXRD studies, which enabled structure-adsorptive property comparisons. An explanation for the different sorption profiles is proposed based on the differences in the two closed phases and chemical composition, which was supported by computational studies that suggest different energy landscapes as a function of unit cell volume, as well as different CO_2_-binding sites for the two frameworks. *In situ* IR measurements provided further experimental validation of the different mechanism of adsorption, confirming the interactions between the frameworks and the adsorbed CO_2_ found by DFT calculations. This contribution therefore affords insights into the mechanism of gate-opening in switching CNs and provides a possibly general crystal engineering approach to tune sorption isotherm shapes. With respect to crystal engineering, the abundance of linker ligands with phenyl and/or azole moieties offers an opportunity to test how substitution of even one linker ligand atom can be used to control both the mechanism of gate-opening and threshold pressure of switching CNs. Studies are underway to address this matter.

## Supporting Information Available

Experimental and computational details, supporting isotherms and PXRD patterns, SEM images, and miscellaneous characterization (PDF)
Crystallographic information files (simulated) for the most stable DFT-optimized binding sites in **X-dia-4-Co** and **X-dia-5-Co** for specific unit cell volumes: **X_dia_4_Co_1st_V1_CO2** (CIF)
**X_dia_4_Co_2nd_V1_CO2** (CIF)
**X_dia_4_Co_3rd_V1_CO2** (CIF)
**X_dia_4_Co_4th_V1_CO2** (CIF)
**X_dia_5_Co_V5_CO2** (CIF)

## Supplementary Material

ja3c01113_si_001.pdf

ja3c01113_si_002.cif

ja3c01113_si_003.cif

ja3c01113_si_004.cif

ja3c01113_si_005.cif

ja3c01113_si_006.cif
