# Supplementary material for: One Atom Can Make All the Difference: Gas-Induced Phase Transformations in Bisimidazole-Linked Diamondoid Coordination Networks
Source: J Am Chem Soc. 2023 Apr 26;145(18):10197–207. doi: 10.1021/jacs.3c01113 (PMC10176468; doi:10.1021/jacs.3c01113)
Supplement: Supplementary file 1 — ja3c01113_si_001.pdf [file ja3c01113_si_001.pdf]

Supporting Information (SI)

One atom can make all the difference: gas-induced phase transformations in bisimidazole linked diamondoid coordination networks

Kyriaki Koupepidou,[a] Varvara I. Nikolayenko,[a] Debobroto Sensharma,[a] Andrey A. Bezrukov,[a] Matthias Vandichel,[a,b] Sousa Javan Nikkhah,[a] Dominic C. Castell,[a] Kolade A. Oyekan,[c] Naveen Kumar,[a] Aizhamal Subanbekova,[a] William G. Vandenberghe,[c] Kui Tan,[c] Leonard J. Barbour[d] and Michael J. Zaworotko*[a,b]

[a] Bernal Institute, Department of Chemical Sciences, University of Limerick, Limerick V94 T9PX, Republic of Ireland.

[b] Advanced Materials and Bioengineering Research (AMBER) Centre, Dublin, D02 R590 Republic of Ireland.

[c] Department of Materials Science and Engineering, University of Texas at Dallas, Richardson, Texas 75080, United States of America.

[d] Department of Chemistry and Polymer Science, University of Stellenbosch, 7602, Matieland, South Africa.

Table of Contents

Methods 8

S1. Materials and Synthesis 8

S2. Single-crystal X-ray Diffraction Measurements 9

S2A. General Procedure 9

S2B. Specific Refinement Details 9

S3. Powder X-ray Diffraction (PXRD) Measurements 12

S4. Pawley Fits for Powder X-ray Diffraction 12

S5. Variable-Temperature Powder X-ray Diffraction (VT-PXRD) Measurements 12

S6. *In situ* Powder X-ray Diffraction 13

S7. Thermogravimetric Analyses (TGA) and Differential Scanning Calorimetry (DSC) Analyses 13

S8. Scanning Electron Microscopy (SEM) and Particle Size Distributions 13

S9. Sorption Studies 14

S9A. Low-pressure Gas Sorption Measurements 14

S9B. Langmuir Surface Areas 14

S9C. High-pressure Gas Sorption Measurements 14

S10. *In situ* Infrared (IR) spectroscopy 15

S11. CMC Simulations and Binding Site Modelling 15

S11A. Periodic Density Functional Theory (DFT) Calculations 15

S11B. Canonical Monte Carlo (CMC) Simulations 16

Figures and Tables 18

References 69

Table of Figures

Figure S1. Asymmetric unit for: (a) **X-dia-4-Co-α**, (b) **X-dia-5-Co-α**, (b) **X-dia-4-Co-β** and (d) **X-dia-5-Co-β**. 18
Figure S2. Two-fold positional disorder for: (a) **bimpy** linker in **X-dia-4-Co-α** and (b) **bimbz** linker in **X-dia-5-Co-α**. 18
Figure S3. Possible positions for the nitrogen atom in the central pyridine ring in **X-dia-4-Co**. 19
Figure S4. Positions **X-dia-4-Co-1st** and **X-dia-4-Co-2nd** for the nitrogen atom of the **bimpy** linker in **X-dia-4-Co**. Positions **X-dia-4-Co-1st** and **X-dia-4-Co-2nd** generate the same combination of two different pore chemistry environments (environments 1 and 2). 19
Figure S5. Positions **X-dia-4-Co-3rd** and **X-dia-4-Co-4th** for the nitrogen atom of the **bimpy** linker in **X-dia-4-Co**. Positions **X-dia-4-Co-3rd** and **X-dia-4-Co-4th** generate the same combination of two different pore chemistry environments (environments 3 and 4). 20
Figure S6. Comparative analysis of the 4-fold interpenetrated diamondoid nets in: (a) **X-dia-4-Co-α**, (b) **X-dia-5-Co-α**, (c) **X-dia-4-Co-β** and (d) **X-dia-5-Co-β.** 22
Figure S7. Comparative analysis of the 4-fold interpenetrated diamondoid nets in: (a) **X-dia-4-Co-α**, (b) **X-dia-5-Co-α**, (c) **X-dia-4-Co-β** and (d) **X-dia-5-Co-β.** 23
Figure S8. TG curves of **X-dia-4-Co-α** (black) and **X-dia-4-Co-β** (red) under N_2 environment. 24
Figure S9. TG curves of **X-dia-5-Co-α** (black) and **X-dia-5-Co-β** (red) under N_2 environment. 24
Figure S10. PXRD patterns for the open phases: (a) **X-dia-4-Co-α** and (b) **X-dia-5-Co-α**. 25
Figure S11. PXRD patterns for the closed phases: (a) **X-dia-4-Co-β** and (b) **X-dia-5-Co-β**. 25
Figure S12. Comparative analysis of the **bimpy** linker conformations along different directions, in the two isolated phases of **X-dia-4-Co**: (a) **X-dia-4-Co-α** and (b) **X-dia-4-Co-β**. 26
Figure S13. Comparative analysis of the **bdc^{2-}** linker conformations along different directions, in the two isolated phases of **X-dia-4-Co**: (a) **X-dia-4-Co-α** and (b) **X-dia-4-Co-β**. 26
Figure S14. Single adamantoid cage representations in: (a) **X-dia-4-Co-α**, (b) **X-dia-5-Co-α**, (c) **X-dia-4-Co-β** and (d) **X-dia-5-Co-β**. 27
Figure S15. Single adamantoid cage representations in: (a) **X-dia-4-Co-α** (disordered), (b) **X-dia-5-Co-α** (disordered), (c) **X-dia-4-Co-β** and (d) **X-dia-5-Co-β**. 27
Figure S16. Single net representations in: in: (a) **X-dia-4-Co-α**, (b) **X-dia-5-Co-α**, (c) **X-dia-4-Co-β**, and (d) **X-dia-5-Co-β**. 28
Figure S17. Coordination environment around the Co^{2+} center in: (a) **X-dia-4-Co-α**, (b) **X-dia-5-Co-α**, (c) **X-dia-4-Co-β** and (d) **X-dia-5-Co-β**. 28
Figure S18. Pawley profile fit of PXRD pattern for **X-dia-5-Co-β**. 30
Figure S19. Comparative analysis of the **bimbz** linker conformations along different directions, in the two isolated phases of **X-dia-5-Co**: (a) **X-dia-5-Co-α** and (b) **X-dia-5-Co-β**. 31
Figure S20. Comparative analysis of the **bdc^{2-}** linker conformations along different directions, in the two isolated phases of **X-dia-5-Co**: (a) **X-dia-5-Co-α** and (b) **X-dia-5-Co-β**. 31
Figure S21. *In situ* variable temperature PXRD patterns of **X-dia-4-Co-α**. 32
Figure S22. *In situ* variable temperature PXRD patterns of **X-dia-5-Co-α.** 32
Figure S23. DSC profile of **X-dia-4-Co-α** for two consecutive cycles. 33
Figure S24. DSC profile of **X-dia-5-Co-α** for two consecutive cycles. 33

Figure S25. Cycling experiments for CO_2 at 195 K. Five consecutive cycles for **X-dia-4-Co** (a) and **X-dia-5-Co** (c). Log plots for five consecutive cycles for **X-dia-4-Co** (b) and **X-dia-5-Co** (d). Adsorption: closed sphere; desorption: open sphere. 34
Figure S26. Low-pressure CO_2 and N_2 sorption isotherms for: (a) **X-dia-4-Co** and (b) **X-dia-5-Co**. Adsorption: closed sphere; desorption: open sphere. 35
Figure S27. Low-pressure C_2H_2, C_2H_4 and C_2H_6 at 298 K sorption isotherms for: (a) **X-dia-4-Co** and (b) **X-dia-5-Co**. Adsorption: closed sphere; desorption: open sphere. 35
Figure S28. (a) High-pressure CO_2 sorption isotherms for **X-dia-4-Co** at 273 K (red) and 298 K (maroon). (b) High-pressure CH_4 sorption isotherm for **X-dia-4-Co** at 298 K. Adsorption: closed sphere; desorption: open sphere. 35
Figure S29. (a) High-pressure CO_2 sorption isotherms for **X-dia-5-Co** at 273 K (red) and 298 K (maroon). Adsorption: closed sphere; desorption: open sphere. (b) Cycling experiment for **X-dia-5-Co** under CO_2 at 298 K between 0 and 20 bar. 36
Figure S30. High-pressure CH_4 sorption isotherm for **X-dia-5-Co** at 298 K. Adsorption: closed sphere; desorption: open sphere. 36
Figure S31. (a) *In situ* PXRD patterns for **X-dia-4-Co** in the presence of CO_2, collected at 298 K in the pressure region of 0 to 1 bar. Red rectangles show highlight the regions with major peak changes. Magnified *in situ* PXRD patterns in the 2θ range: (b) 15˚ to 17˚ and (c) 21˚ to 23.5˚. 37
Figure S32. The *in situ* CO_2-loaded PXRD pattern of **β** phases at 35 bar and 298 K differ from the calculated PXRD pattern of the as-synthesised **α** phases: (a) **X-dia-4-Co** and (b) **X-dia-5-Co**. 37
Figure S33. Profile fit of *in situ* PXRD pattern of **X-dia-4-Co-β** at 0 bar and 298 K. 38
Figure S34. Profile fit of *in situ* CO_2-loaded PXRD pattern of **X-dia-4-Co-β** at 3 bar and 298 K. 38
Figure S35. Profile fit of *in situ* CO_2-loaded PXRD pattern of **X-dia-4-Co-β** at 5 bar and 298 K. 39
Figure S36. Profile fit of *in situ* CO_2-loaded PXRD pattern of **X-dia-4-Co-β** at 10 bar and 298 K. 39
Figure S37. Unit cell parameters of **X-dia-4-Co-β** obtained from Pawley profile fits of high-pressure *in situ* CO_2-loaded PXRD patterns at 298 K. 40
Figure S38. Unit cell parameters of **X-dia-4-Co-β** obtained from Pawley profile fits of low- and high-pressure *in situ* CO_2-loaded PXRD patterns at 298 K (stars: obtained from low-pressure region of 0 to 1 bar; spheres: obtained from high-pressure region of 1 to 10 bar – see Section S6 for experimental details). 41
Figure S39. Profile fit of *in situ* PXRD pattern of **X-dia-5-Co-β** at 0 bar and 298 K. 42
Figure S40. Profile fit of *in situ* CO_2-loaded PXRD pattern of **X-dia-5-Co-β** at 3 bar and 298 K. 42
Figure S41. Profile fit of *in situ* CO_2-loaded PXRD pattern of **X-dia-5-Co-β** at 5 bar and 298 K. 43
Figure S42. Profile fit of *in situ* CO_2-loaded PXRD pattern of **X-dia-5-Co-β** at 10 bar and 298 K. 43
Figure S43. Profile fit of *in situ* CO_2-loaded PXRD pattern of **X-dia-5-Co-β** at 35 bar and 298 K. 44
Figure S44. Unit cell parameters of **X-dia-5-Co-β** obtained from Pawley profile fits of high-pressure *in situ* CO_2-loaded PXRD patterns at 298 K. 45
Figure S45. SEM images. Crystal morphology of **X-dia-4-Co-β** (a) and **X-dia-5-Co-β** (b). Representative images for the bulk phase of **X-dia-4-Co-β** (c) and **X-dia-5-Co-β** (d). Examples of crystal length (orange) and crystal width (red) are marked. 47
Figure S46. Crystal size distributions for **X-dia-4-Co-β**: (a) crystal length and (b) crystal width. 48

Figure S47. Crystal width plotted against crystal length for **X-dia-4-Co-β**. 48
Figure S48. Crystal size distributions for **X-dia-5-Co-β**: (a) crystal length and (b) crystal width. 49
Figure S49. Crystal width plotted against crystal length for **X-dia-5-Co-β**. 49
Figure S50. SEM images post CO_2 sorption for: (a) **X-dia-4-Co** and (b) **X-dia-5-Co**. 50
Figure S51. Comparison of hydrogen bonding in the two closed phases: (a) **X-dia-4-Co-β** and (b) **X-dia-5-Co-β**. 50
Figure S52. IR spectra of **bimbz**, **bimpy** and **H_2bdc** ligands. All the spectra are referenced to blank KBr pellet. 51
Figure S53. Calculated spectra of **bimbz** and **bimpy** ligands. 52
Figure S54. Calculated vibrational modes of: (a-h) **bimbz** and (i, j) **bimpy** linkers that show significant changes upon loading of CO_2. The frequency positions are determined from IR spectra of activated **X-dia-5-Co** and **X-dia-4-Co**, respectively (see Figure 5). The yellow arrows represent the eigenvectors of the vibrational modes. Color scheme: Grey = C, blue = N, and white = H. 53
Figure S55. IR spectra of: (a) **X-dia-5-Co** and (b) **X-dia-4-Co** upon loading CO_2 as a function of pressure. The signal of gas phase CO_2 spectra including stretching (ν_{as}), bending (β), and combination ($\nu_{as} + \nu_s$, ν_{as}+β) bands is out of scale. All the spectra are referenced to blank KBr pellet. 53
Figure S56. High-pressure CO_2 isotherms collected at 298 K for **X-dia-4-Co** (a) and **X-dia-5-Co** (b), expressed in molecules of CO_2/unit cell as a function of pressure. 54
Figure S57. Visualization from different view angles of CO_2 binding site isosurfaces from CMC simulations with 8 adsorbates in **X-dia-4-Co-1st**, (a), (b), and (c) **X-dia-4-Co-1st** (V_0 = 2×1962.02 Å^3); (d), (e), and (f) **X-dia-4-Co-1st** (V_1 = 2×2036.28 Å^3); (g), (h), and (i) **X-dia-4-Co-1st** (V_2 = 2×2106.73 Å^3). Color codes: N, blue; Co, purple; H, white; C, grey; O, red..... 55
Figure S58. Visualization from different view angles of CO_2 binding site isosurfaces from CMC simulations with 8 adsorbates in **X-dia-4-Co-2nd**, (a), (b), and (c) **X-dia-4-Co-2nd** (V_0 = 2×1962.02 Å^3); (d), (e), and (f) **X-dia-4-Co-2nd** (V_1 = 2×2036.28 Å^3); (g), (h), and (i) **X-dia-4-Co-2nd** (V_2 = 2×2106.73 Å^3). Color codes: N, blue; Co, purple; H, white; C, grey; O, red.... 56
Figure S59. Visualization from different view angles of CO_2 binding site isosurfaces from CMC simulations with 8 adsorbates in **X-dia-4-Co-3rd**, (a), (b), and (c) **X-dia-4-Co-3rd** (V_0 = 2×1962.02 Å^3); (d), (e), and (f) **X-dia-4-Co-3rd** (V_1 = 2×2036.28 Å^3); (g), (h), and (i) **X-dia-4-Co-3rd** (V_2 = 2×2106.73 Å^3). Color codes: N, blue; Co, purple; H, white; C, grey; O, red. ... 57
Figure S60. Visualization from different view angles of CO_2 binding site isosurfaces from CMC simulations with 8 adsorbates in **X-dia-4-Co-4th**, (a), (b), and (c) **X-dia-4-Co-4th** (V_0 = 2×1962.02 Å^3); (d), (e), and (f) **X-dia-4-Co-4th** (V_1 = 2×2036.28 Å^3); (g), (h), and (i) **X-dia-4-Co-4th** (V_2 = 2×2106.73 Å^3). Color codes: N, blue; Co, purple; H, white; C, grey; O, red. ... 58
Figure S61. Visualization from different view angles of CO_2 binding site isosurfaces from CMC simulations in **X-dia-5-Co**, (a), (b), and (c) **X-dia-5-Co** (V_2 = 2×2072.40 Å^3), there are 2 CO_2 adsorption positions per unit cell; (d), (e), and (f) **X-dia-5-Co** (V_3 = 2×2149.89 Å^3), there are 3 CO_2 adsorption positions per unit cell; (g), (h), and (i) **X-dia-5-Co** (V_4 = 2×2223.40 Å^3), there are 4 CO_2 adsorption positions per unit cell. Color codes: N, blue; Co, purple; H, white; C, grey; O, red. 59
Figure S62. Visualization from different view angles of CO_2 binding site isosurfaces from CMC simulations with 8 adsorbate molecules in **X-dia-5-Co**, (a), (b), and (c) **X-dia-5-Co** (V_5 = 2×2292.98 Å^3); (d), (e), and (f) **X-dia-5-Co** (V_6 = 2×2351.19 Å^3). Color codes: N, blue; Co, purple; H, white; C, grey; O, red. 60
Figure S63. Visualization of CO_2 binding site isosurfaces from CMC simulations together with CO_2 coordinates from the DFT-optimized structures for **X-dia-4-Co-1st** (V_1 = 2×2036.27 Å^3) framework. Color codes: N, blue; Co, purple; H, white; C, grey; O, red. CO_2 molecules are presented in yellow for clarification. 61

Figure S64. Visualization of CO_2 binding site isosurfaces from CMC simulations together with CO_2 coordinates from the DFT-optimized structures for **X-dia-4-Co-2nd** (V_1 = 2×2036.27 Å^3) framework. Color codes: N, blue; Co, purple; H, white; C, grey; O, red. CO_2 molecules are presented in yellow for clarification. 61
Figure S65. Visualization of CO_2 binding site isosurfaces from CMC simulations together with CO_2 coordinates from the DFT-optimized structures for **X-dia-4-Co-3rd** (V_1 = 2×2036.27 Å^3) framework. Color codes: N, blue; Co, purple; H, white; C, grey; O, red. CO_2 molecules are presented in yellow for clarification. 62
Figure S66. Visualization of CO_2 binding site isosurfaces from CMC simulations together with CO_2 coordinates from the DFT-optimized structures for **X-dia-4-Co-4th** (V_1 = 2×2036.27 Å^3) framework. Color codes: N, blue; Co, purple; H, white; C, grey; O, red. CO_2 molecules are presented in yellow for clarification. 62
Figure S67. Visualization of CO_2 binding site isosurfaces (blue) from CMC simulations together with CO_2 coordinates from the DFT-optimizations (yellow, ball and stick) for **X-dia-5-Co** (a) V_2 = 2×2072.40 Å^3, (b) V_3 = 2×2149.89 Å^3, (c) V_4 = 2×2223.40 Å^3 and (d) V_5 = 2×2292.98 Å^3 frameworks. Color codes: N, blue; Co, purple; H, white; C, grey; O, red. CO_2 molecules are presented in yellow for clarification. 63
Figure S68. PXRD patterns for the calculated DFT-optimized CO_2-loaded **X-dia-4-Co-β** for V_1 = 2036.27 Å^3 (black) and experimental CO_2-loaded **X-dia-4-Co-β** at 10 bar (red). 64
Figure S69. PXRD patterns for the calculated DFT-optimized CO_2-loaded **X-dia-5-Co-β** for V_5 = 2292.98 Å^3 (black) and experimental CO_2-loaded **X-dia-5-Co-β** at 10 bar (red). 64
Figure S70. Binding sites of CO_2 in **X-dia-5-Co** for V_2 = 2072.40 Å^3 (a), V_3 = 2149.89 Å^3 (b), V_4 = 2223.40 Å^3 (c) and V_5 = 2292.98 Å^3 (d) obtained with DFT calculations. Selected close contact distances are shown in black dashed lines, while the shortest close contact distance in each framework is listed (in Å). 65
Figure S71. DSC analysis of **X-dia-4-Co-β** and **X-dia-5-Co-β** upon sorption of CO_2 at 198 K. (A) Adsorption and desorption peaks for three different physical samples for each compound. (B) Magnified adsorption peaks. 68

Table of Tables

Table S1. Crystallographic data and refinement parameters for **X-dia-4-Co** and **X-dia-5-Co**. 21
Table S2. List of angles around the Co^{2+} center in **X-dia-4-Co** and **X-dia-5-Co**. 29
Table S3. List of distances around the Co^{2+} center in **X-dia-4-Co** and **X-dia-5-Co**. 29
Table S4. Comparison of unit cell parameters of **X-dia-5-Co-β** obtained by SCXRD and Pawley profile fit of the experimental PXRD pattern. 30
Table S5. Langmuir fitting for **X-dia-4-Co** and **X-dia-5-Co**. 34
Table S6. Unit cell parameters of **X-dia-4-Co-β** obtained from Pawley profile fits of in situ CO_2-loaded PXRD patterns at 298 K in the low-pressure region (top; 0 to 1 bar) and high-pressure region (bottom; 0 to 10 bar). 46
Table S7. Unit cell parameters of **X-dia-5-Co-β** obtained from Pawley profile fits of in situ CO_2-loaded PXRD patterns at 298 K in the high pressure region (0 to 35 bar). 46
Table S8. Summary of selected phonon modes of **X-dia-5-Co** and **X-dia-4-Co**. Notations and acronyms: **ν**, stretch; **δ**, in plane deformation; **γ**, out of plane deformation; **β**, bend; **ph**, phenyl; **az**, azole; **s**, symmetric; and **as**, asymmetric. 52
Table S9. Relative energies (kJ/mol per mol unit cell) of four of the different possible structures of **X-dia-4-Co** obtained with DFT calculations at the experimental cell parameters (**Table S1**). For the N atom positions see Figures S3-S5. 54
Table S10. Adsorption enthalpy and Gibbs free energy for CO_2 in **X-dia-4-Co** compared to images with relevant volumes for **X-dia-5-Co.** Cell parameters were kept fixed during optimization of all structures while, atomic positions of empty host and empty host + CO_2 completely relaxed. For all **X-dia-4-Co** structures, the cell volume is 2036.27 Å^3 and cell parameters are: a=14.120 Å, b=17.765 Å, c=8.118 Å, α=β=γ=90º. compare to the optimized the second, third and fourth NEB image for X-dia-5-Co ($V_{2,\mathbf{Xdia\text{-}5}}$ = 2072.40 Å^3, a=14.923 Å, b=8.367 Å, c=16.598 Å, α=β=γ=90º; $V_{3,\mathbf{Xdia\text{-}5}}$ = 2149.89 Å^3, a=14.820 Å, b=8.842 Å, c=16.406 Å, α=β=γ=90º; $V_{4,\mathbf{Xdia\text{-}5}}$ = 2223.40 Å^3, a=14.717 Å, b=8.318 Å, c=16.213 Å, α=β=γ=90º; $V_{5,\mathbf{Xdia\text{-}5}}$ = 2292.98 Å3, a=14.6131 Å, b=9.79434 Å, c=16.0208 Å, α=β=γ=90º). 54
Table S11. Lennard-Jones (LJ) parameters representing framework atoms. The interaction with the sorbate molecules was calculated using Lorentz-Berthelot mixing rules. 55
Table S12. Point charges for CO_2. 55
Table S13. Short contact distances between the framework and the adsorbed CO_2 molecule in the open phases of **X-dia-4-Co-1st, X-dia-4-Co-2nd, X-dia-4-Co-3rd and X-dia-4-Co-4th** at V_1 = 2036.27 Å^3 and **X-dia-5-Co** at V_2 = 2072.40 Å^3, V_3 = 2149.89 Å^3, V_4 = 2223.40 Å^3 and V_5 = 2292.98 Å^3, optimized by DFT. 67

Methods

S1. Materials and Synthesis

The linkers 1,4-bis-(1H-imidazol-1-yl)benzene (**bimbz**) and 2,5-bis(1H-imidazol-1-yl)pyridine (**bimpy**) were synthesized with modified reported procedures.[1, 2] Other reagents and solvents were commercially available and were used without further purification.

Synthesis of bimbz. 1,4-dibromobenzene (5.0 g, 21.2 mmol, 1.0 eq), CuI (805 mg, 20 mol%), imidazole (4.33 g, 63.6 mmol, 3.0 eq) and K_2CO_3 (8.78 g, 63.6 mmol, 3.0 eq) were all added to anhydrous DMF (50 ml) under N_2. The resulting reaction mixture was then heated to 150 °C for 48 h under an atmosphere of N_2. After cooling to room temperature, the mixture was diluted with DCM (250 ml) and filtered. The filtered organic layer was transferred to a large separating funnel and washed twice with H_2O (2 × 500 ml). After drying over $MgSO_4$, the organic layer was concentrated under reduced pressure. Final purification was achieved by rapid trituration of the compound from a DCM/hexane mixture, affording **bimbz** as a white solid (4,10 g, 92%). All characterization data matches well with literature reported values.[1]

Synthesis of bimpy. 2,5-dibromopyridine (5.0 g, 21.1 mmol, 1.0 eq), CuI (801 mg, 20 mol%), imidazole (4.30 g, 63.3 mmol, 3.0 eq) and K_2CO_3 (8.73 g, 63.3 mmol, 3.0 eq) were all added to anhydrous DMF (50 ml) under N_2. The resulting reaction mixture was then heated to 150 °C for 48 h under an atmosphere of N_2. After cooling to room temperature, the mixture was diluted with DCM (250 ml) and filtered. The filtered organic layer was transferred to a large separating funnel and washed twice with H_2O (2 × 500 ml). After drying over $MgSO_4$, the organic layer was concentrated under reduced pressure. Final purification was achieved by rapid trituration of the compound from a DCM/hexane mixture, affording **bimpy** as a white solid (3.34 g, 75%). All characterization data matches well with literature reported values.[2]

Synthesis of X-dia-4-Co-α ([Co(bdc)(bimpy)]·0.5DMA). A mixture of $Co(NO_3)_2·6H_2O$ (15 mg, 0.05 mmol), H_2bdc (17 mg, 0.1 mmol), bimpy (11 mg, 0.05 mmol) and DMA (8 mL) was added to a 28-mL glass vial. The vial was capped tightly, ultrasonicated for 5 minutes and then placed in an oven at 105 °C. After 24 hours, the vial was removed from the oven and allowed to cool to room temperature. Purple block shaped crystals were harvested by filtration and washed with DMA. Yield: 55%.

Synthesis of X-dia-4-Co-β ([Co(bdc)(bimpy)]). The closed phase **X-dia-4-Co-β** was obtained by heating the **X-dia-4-Co-α** phase at 85 °C under vacuum for 24 h. IR (cm^{-1}): 3131(m), 3054(w), 1592(s), 1499(s), 1340(s), 1305(s), 1241(m), 1142(w), 1114(w), 1072(s), 1015(w), 964(s), 938(m), 891(w), 824(s), 748(s).

Synthesis of X-dia-5-Co-α ([Co(bdc)(bimbz)]·0.5DMA). Single crystals suitable for X-ray analysis were obtained by a similar method as described for **X-dia-4-Co-α**, by using **bimbz** (11 mg, 0.05 mmol) instead of **bimpy**. Yield: 55%.

Synthesis of X-dia-5-Co-β ([Co(bdc)(bimpy)]). The closed phase **X-dia-5-Co-β** was obtained by heating the **X-dia-4-Co-α** phase at 85 °C under vacuum for 24 h. IR (cm^{-1}): 3126(m), 3048(w), 1578(s), 1527(s), 1500(m), 1341(s), 1304(s), 1243(m), 1129(m), 1104(w), 1068(s), 1014(w), 961(s), 943(m), 890(w), 826(s), 751(s).

Bulk synthesis. Bulk synthesis of **X-dia-4-Co-α** and **X-dia-5-Co-**α was performed by scaling up the crystallization reagents and solvent by 10 times. The large batches were used for activation, SEM analysis, sorption measurements, and miscellaneous characterization.

S2. Single-crystal X-ray Diffraction Measurements

S2A. General Procedure

Suitable single crystals of **X-dia-4-Co** and **X-dia-5-Co** were chosen for single-crystal X-ray diffraction measurements. Diffraction data for **X-dia-4-Co-α, X-dia-4-Co-β** and **X-dia-5-Co-α** were collected at 100 K on a Bruker D8 Quest diffractometer equipped with a CuKα microfocus source (λ = 1.5406 Å) and a Photon 100 detector. Diffraction data for **X-dia-5-Co-β** were collected at 150 K on a Bruker D8 Quest diffractometer equipped with a MoKα microfocus source (λ = 0.71073 Å) and a Photon 100 detector. All attempts to recollect data for **X-dia-5-Co-β** at 100 K on a CuKα source have failed, due to poor quality of the single crystals, caused by the extreme structural transformation from the open phase to the closed phase. Therefore, cell parameters cannot be directly compared, but differences in angles, distances and guest accessible space should remain comparable in the margin of 50 K. In all cases, data was indexed, integrated and scaled in APEX4.[3] An absorption correction was performed using the multi-scan method within SADABS.[4] Space group determination was performed simultaneously with structure solution using SHELXT intrinsic phasing methods and the solution was refined on F2 using SHELXL non-linear least squares implemented in Olex2 v1.2.10.[5] Anisotropic thermal parameters were applied to all non-hydrogen atoms. All the hydrogen atoms were generated geometrically. X-ray experimental data and refinement parameters are given in Table S1. The final crystal structures have been deposited in the Cambridge Crystallographic Data Centre (CCDC 2181241-2181244).

S2B. Specific Refinement Details

The structures reported in this manuscript are all metal-organic framework structures whose crystals are weakly diffracting, are prone to showing twinned reflections and have significant diffuse scattering from guests in the pore network. Due to the flexible nature of the frameworks, all the structures suffer from some degree of positional disorder of the imidazoles of the **bimbz** and **bimpy** linkers. Furthermore, in the case of **X-dia-4-Co**, the asymmetric linker **bimpy** generates substitutional disorder in the central ring of the linker. These factors create challenges for the structural determination of the crystals and are shown by a handful of B level alerts.

Additionally, the as-synthesized crystals undergo significant transformations from open to closed phases, which leads to significant decrease of crystal quality and triggers additional alerts in the guest-free phases (A level alerts in the case of **X-dia-5-Co-β**). Therefore, standard measures of refinement, such as R1, wR2 and GooF can be higher than typical crystals of close-packed inorganic structures. All A and B level alerts relate to issues with the data collection, data quality or structural models, and are all addressed in the following section.

X-dia-4-Co-a. This sample suffers from positional disorder around the **bimpy** linker and carboxylate moiety atoms, causing several B level alerts for the related atoms. The positional disorder around imidazole rings was successfully modelled, but the disorder on the carboxylate moiety could not be modelled with satisfactory refinement. The position of the nitrogen atom in the central ring of the **bimpy** linker could not be accurately determined from the electron density values. Therefore, the two configurations of the **bimpy** linker were modelled to have two nitrogen atoms in opposite positions in the central ring to account for the substitutional disorder, and these two configurations were linked through a free variable.

Alert level A

PLAT601_ALERT_2_A Unit Cell Contains Solvent Accessible VOIDS of . 323 Ang**3

Response: X-dia-4-Co-α is a porous phase with rectangular channels.

Alert level B

PLAT241_ALERT_2_B High 'MainMol' Ueq as Compared to Neighbors of O1 Check
PLAT241_ALERT_2_B High 'MainMol' Ueq as Compared to Neighbors of O2 Check
PLAT242_ALERT_2_B Low 'MainMol' Ueq as Compared to Neighbors of Co1 Check
PLAT341_ALERT_3_B Low Bond Precision on C-C Bonds .... 0.0155 Ang.
PLAT973_ALERT_2_B Check Calcd Positive Resid. Density on Co1 1.77 eA-3

Response: Owing to the highly porous nature of **X-dia-4-Co-α** coupled with the reduced crystal quality of the as-synthesized phase, the authors acknowledge B level alerts associated with residual electron density, increased thermal motion and substitutional disorder. Despite numerous attempts, a second position of disorder for the **bdc**$^{2-}$ linker could not be determined, although we were able to model a second position for the **bimpy** linker. We ascribe the reduced crystal quality to a combination of high degree of porosity and flexibility.

X-dia-4-Co-β. This sample was activated using heat and vacuum. In this case, diffraction data is good enough to avoid level A and B alerts. This is in agreement with the retention of the same space group with **X-dia-4-Co-α**, which suggests a milder transformation and, thus, less mechanical stress on the crystals and reasonable diffraction data. Like the open phase, **X-dia-4-Co-β** shows substitutional disorder. Positional disorder is not present in this case, so there is only one configuration of the **bimpy** linker. The two possible positions in the central ring were split between carbon and nitrogen. Refinement with free occupancies was possible, resulting in occupancies of 0.500 and 0.500 for atoms C9A and N3A, respectively, for position A, and atoms C9B and N3B, for position B.

X-dia-5-Co-a. This sample suffers from positional disorder around the **bimbz** linker, causing several B level alerts. The positional disorder around imidazole rings was successfully modelled.

Alert level A

PLAT601_ALERT_2_A Unit Cell Contains Solvent Accessible VOIDS of .345 Ang**3

Response: X-dia-5-Co-α is a porous phase with rectangular channels.

Alert level B

PLAT084_ALERT_3_B High wR2 Value (i.e. > 0.25) .... 0.39 Report
PLAT215_ALERT_3_B Disordered C16 has ADP max/min Ratio ..... 4.6 Note
PLAT934_ALERT_3_B Number of (Iobs-Icalc)/Sigma(W) > 10 Outliers .. 2 Check
PLAT973_ALERT_2_B Check Calcd Positive Resid. Density on Co1 1.64 eA-3

Response: As with **X-dia-4-Co-α**, **X-dia-5-Co-α** exhibits B level alerts associated with reduced crystal quality (which we believe to arise from the presence of large voids and high degree of flexibility) and increased thermal motion.

X-dia-5-Co-β. This sample was activated using heat and vacuum, and, as a result, the crystal quality has been significantly impacted. The measures of data quality (intensity, R_{int},

completeness to standard theta limits) are impacted. Unlike the case of **X-dia-4-Co**, the structural transformation from **X-dia-5-Co-α** to **X-dia-5-Co-β** is accompanied by a change in space group, which leads to a larger change in lattice parameters and reflects on the quality of diffraction data. This triggers several A level alerts for atoms involved in the imidazole and phenyl rings. As discussed, A and B level alerts associated with Flack parameter and R_{int} value can be attributed to the poor data quality. Despite these issues, the bulk phase of the material is in agreement this crystallographic model, as confirmed by experimental PXRD patterns (Section S3). PXRD indexing was also performed as an additional technique to confirm the integrity of the crystallographic model (Section S4), which showcased a sufficient calculated match to the experimental dataset.

Alert level A

PLAT026_ALERT_3_A Ratio Observed / Unique Reflections (too) Low .. 24% Check
PLAT031_ALERT_4_A Refined Extinction Parameter Within Range of ... 0.500 Sigma
PLAT084_ALERT_3_A High wR2 Value (i.e. > 0.25) .... 0.49 Report
PLAT234_ALERT_4_A Large Hirshfeld Difference N1 --C9 . 0.32 Ang.
PLAT234_ALERT_4_A Large Hirshfeld Difference N2 --C12 . 0.33 Ang.
PLAT234_ALERT_4_A Large Hirshfeld Difference C2 --C7 . 0.39 Ang.
PLAT234_ALERT_4_A Large Hirshfeld Difference C5 --C6 . 0.35 Ang.
PLAT234_ALERT_4_A Large Hirshfeld Difference C6 --C7 . 0.41 Ang.
PLAT234_ALERT_4_A Large Hirshfeld Difference C10 --C11 . 0.39 Ang.
PLAT234_ALERT_4_A Large Hirshfeld Difference C12 --C13 . 0.37 Ang.

Alert level B

RINTA01_ALERT_3_B The value of Rint is greater than 0.18 Rint given 0.186
PLAT020_ALERT_3_B The Value of Rint is Greater Than 0.12 .... 0.186 Report
PLAT234_ALERT_4_B Large Hirshfeld Difference N1 --C10 . 0.26 Ang.
PLAT234_ALERT_4_B Large Hirshfeld Difference N2 --C9 . 0.28 Ang.
PLAT234_ALERT_4_B Large Hirshfeld Difference C16 --C17 . 0.26 Ang.
PLAT241_ALERT_2_B High 'MainMol' Ueq as Compared to Neighbors of O2 Check
PLAT242_ALERT_2_B Low 'MainMol' Ueq as Compared to Neighbors of Co1 Check
PLAT341_ALERT_3_B Low Bond Precision on C-C Bonds .... 0.03438 Ang.
PLAT987_ALERT_1_B The Flack x is >> 0 - Do a BASF/TWIN Refinement Please Check

Response: The authors acknowledge the presence of A and B level alerts associated with reduced crystal quality arising from diffuse electron scattering. Numerous attempts at recollecting better quality data were performed, however owing to the significant structural transformation that occurs between **X-dia-5-Co-α** and **X-dia-5-Co-β** (in addition to the contraction, **X-dia-5-Co-β** experiences a change in symmetry, where it converts from *Pnna* to $Pna2_1$) no better dataset could be obtained.

S3. Powder X-ray Diffraction (PXRD) Measurements

Powder X-ray diffraction data was collected on crushed microcrystalline samples. Diffractograms were recorded using a PANalytical Empyrean™ diffractometer equipped with a PIXcel3D detector, operating in scanning line detector mode with an active length of 4 utilizing 255 channels, in the Continuous Scanning mode with the goniometer in the theta-theta orientation. The diffractometer is fitted with an Empyrean Cu LFF (long fine-focus) HR (9430 033 7310x) tube operated at 40 kV and 40 mA, and CuKα radiation (λα = 1.540598 Å) was used for diffraction experiments. Incident beam optics included the Fixed Divergences slit with anti-scatter slit PreFIX module, with a 1/8° divergence slit and a 1/4° anti-scatter slit, as well as a 10 mm fixed incident beam mask and a Soller slit (0.04 rad). Divergent beam optics included a P7.5 anti-scatter slit, a Soller slit (0.04 rad), and a Ni-β filter. The data was collected from 5°-40° (2θ) with a step-size of 0.016413° and a varied scan time of 30-200 seconds per step.

S4. Pawley Fits for Powder X-ray Diffraction

Unit cell parameters of **X-dia-5-Co-β** were determined from powder X-ray diffraction pattern collected at 298 K using a PANalytical Empyrean™ diffractometer equipped with a PIXcel3D detector. Initial unit cell parameters were determined from positions of 19 observed peaks using indexing via DICVOL[6] implemented in DASH.[7] The found unit cell parameters were then refined via Pawley profile fit of the powder X-ray diffraction pattern in the 10-40˚ 2θ range using GSAS-II[8] (Figure S18). A "chebyschev-1" background function with 2 terms was used. The refined unit cell parameters are: SG: $Pna2_1$, a = 15.4796(11) Å, b = 7.55079(17) Å, c = 16.2363(12) Å, α = β = γ = 90°, V = 1897.75(10) $Å^3$, wR = 2.86 %.

Pawley profile fitting for the CO_2-loaded PXRD patterns was performed in the same way as described above. Despite all efforts, indexing of the PXRD pattern for **X-dia-4-Co-β** at 35 bar and 298 K was not successful. Results for **X-dia-4-Co** and **X-dia-5-Co** are summarized in Tables S6 and S7, respectively.

S5. Variable-Temperature Powder X-ray Diffraction (VT-PXRD) Measurements

Diffractograms at different temperatures were recorded using a PANalytical X'Pert Pro-MPD diffractometer equipped with a PIXcel3D detector, operating in scanning line detector mode with an active length of 4 utilizing 255 channels. Anton Paar TTK 450 stage coupled with the Anton Paar TCU 110 Temperature Control Unit was used to record the variable-temperature diffractograms. The diffractometer was outfitted with an Empyrean Cu LFF (long fine-focus) HR (9430 033 7300x) tube operated at 40 kV and 40 mA and CuKα radiation (λα = 1.54056 Å). Continuous scanning mode with the goniometer in the theta-theta orientation was used to collect the data. Incident beam optics included a 1/4° divergence slit and a Soller slit (0.04 rad). Divergent beam optics included a P7.5 anti-scatter slit, a Soller slit (0.04 rad) and a Ni-β filter. In a typical experiment, ~ 20 mg of sample was crushed to microcrystalline powder and was loaded on a zero background sample holder made for Anton Paar TTK 450 chamber. Each sample was heated up to 200 ˚C under N_2 atmosphere and then cooled back to room temperature. The data was collected from 5°-40° (2θ) with a step-size of 0.016413° and a scan time of 200 seconds per step.

S6. *In situ* Powder X-ray Diffraction

High-pressure experiments (from 0 to 35 bar) were carried out on a PANalytical X'Pert PRO instrument with Debye-Scherrer geometry. Intensity data were recorded using an X'Celerator detector, and 2θ scans in the range of 3−35° were performed with a step size of 0.016° at the scan speed of 0.02 (°/s). During the experiment the powdered sample was exposed to Cu Kα radiation (λ = 1.5418 Å). The activated samples of **X-dia-4-Co-β** and **X-dia-5-Co-β** were individually sealed within a glass capillary (environmental gas cell) and evacuated *ex situ*. Each capillary was then progressively loaded with CO_2 *ex situ* (the pressures selected correspond to events observed in the gas sorption isotherms collected at the same temperature) and variable-pressure PXRD patterns were measured at a constant temperature of 298 K.

Low-pressure experiments (from 0 to 1 bar) were recorded using a PANalytical X'Pert Pro-MPD diffractometer equipped with a PIXcel3D detector. Anton Paar TTK 450 stage coupled with the Anton Paar TCU 110 Temperature Control Unit was used to record the gas loading experiments at a set temperature. The diffractometer is outfitted with an Empyrean Cu LFF (long fine-focus) HR (9430 033 7300x) tube operated at 40 kV and 40 mA and CuKα radiation (λα = 1.54056 Å). Continuous scanning mode with the goniometer in the theta-theta orientation was used to collect the data. Incident beam optics included a 1/4° divergence slit and a Soller slit (0.04 rad). Divergent beam optics included a P7.5 anti-scatter slit, a Soller slit (0.04 rad) and a Ni-β filter. In a typical experiment, ~ 20 mg of sample was crushed to microcrystalline powder and was loaded on a zero background sample holder made for Anton Paar TTK 450 chamber. The sample was subsequently loaded with CO_2 gas and the pressure was monitored using a CG16K capsule dial gauge. Variable-pressure PXRD patterns were measured at a constant temperature of 298 K.

S7. Thermogravimetric Analyses (TGA) and Differential Scanning Calorimetry (DSC) Analyses

Thermogravimetric analysis (TGA) was performed using a TA Instruments Q50 system. Samples were loaded into aluminum sample pans and heated at 10 °C/min from room temperature to 550 °C under N_2 flow. Differential scanning calorimetry (DSC) analysis was performed on a Q2000 TA Instruments system.

For Figures S23 and S24, samples of **X-dia-4-Co-α** and **X-dia-5-Co-α** were loaded into aluminum sample pans with a pinhole on the lids. The experiments were performed at heating rate of 5 °C/min from room temperature to 250 °C under N_2 atmosphere. For Figure S71, samples of **X-dia-4-Co-β** and **X-dia-5-Co-β** were loaded into aluminium sample pans with no lid and activated *in situ* at 85 ˚C. The system was left to equilibrate at 198 K under N_2 flow. The adsorption was performed under CO_2 flow of 10 ml/min, followed by the desorption performed under N_2 flow of 10 ml/min.

S8. Scanning Electron Microscopy (SEM) and Particle Size Distributions

Scanning electron microscopy measurements were carried out for the activated samples **X-dia-4-Co-β** and **X-dia-5-Co-β** to ensure particle size uniformity. The images were collected on a Hitachi SU-70 instrument, using 3 kV acceleration voltage and a working distance of 15 mm. Before the measurement, the samples were dispersed on carbon tape attached to SEM stubs, and were gold-coated for 50 seconds to enhance surface conductivity. SEM images of the crystals were analyzed by ImageJ software and the corresponding crystal size distributions were

calculated manually in the same software (ImajeJ, 2021).[9] For irregular particles, the longest edge was considered as crystal length, and the crystal width was then determined as the width at half length. A normal distribution fitting was performed in each case.

S9. Sorption Studies

For gas sorption experiments, high-purity gases were used as received from BOC Gases Ireland: CO_2 (99.995%), N_2 (99.9995%%) CH_4 (99.9995%), C_2H_2 (98.5%), C_2H_4 (99.92%), C_2H_6 (99.0%). A Micromeritics 3Flex surface area and pore size analyzer 3500 was used for collecting the low-pressure sorption isotherms for CO_2 and N_2. The temperature at 77 K was maintained using a 4 L Dewar filled with liquid nitrogen. The temperature at 195 K was maintained using a 4 L Dewar filled with a dry ice-acetone mixture. Bath temperatures of 273 and 298 K were precisely controlled with a Julabo ME (v.2) recirculating control system containing a mixture of ethylene glycol and water. A Hiden Isochema XEMIS-001 gravimetric sorption analyzer was used for collecting the high-pressure sorption isotherms for CO_2 and CH_4. Prior to experiments, all samples were activated on a SmartVacPrep™ using dynamic vacuum and heating overnight at 358 K.

S9A. Low-pressure Gas Sorption Measurements

The low-pressure sorption isotherms for N_2 at 77 K and CO_2 at 195 K were measured using a Micromeritics 3Flex instrument. Before each gas sorption experiment, the freshly prepared samples of **X-dia-4-Co-α** and **X-dia-5-Co-α** were placed in quartz tubes and activated under high vacuum at 85 ˚C on a Micromeritics SmartVacPrep for 12 hrs, to remove the solvent molecules prior to measurements. This process generated the respective closed phases **X-dia-4-Co-β** and **X-dia-5-Co-β**, as confirmed by PXRD measurements (Section S3). All low-pressure gas sorption studies were performed on the activated phases **X-dia-4-Co-β** and **X-dia-5-Co-β**. Cycling experiments were conducted using the sample in the same tube. Where desorption was not complete (**X-dia-4-Co-β**), the sample was reactivated on the SmartVacPrep system at room temperature for 2 hours.

S9B. Langmuir Surface Areas

The surface areas of **X-dia-4-Co** and **X-dia-5-Co** were determined from the CO_2 adsorption isotherms of **X-dia-4-Co-β** and **X-dia-5-Co-β** (in the range of 0.01 to 1 bar and 0.17 to 1 bar, respectively) collected at 195 K, by applying the Langmuir models. Brunauer-Emmett-Teller (BET) surface areas cannot be accurately determined for either one of the frameworks, because of the flexible/switching behavior towards CO_2.

S9C. High-pressure Gas Sorption Measurements

High-pressure sorption isotherms were recorded on a Hiden Isochema XEMIS-001 gravimetric sorption analyzer at 273 K and 298 K for CO_2, and at 298 K for CH_4. Before running high-pressure isotherm measurements, the samples of **X-dia-4-Co-α** and **X-dia-5-Co-α** were activated under dynamic vacuum on a Micromeritics SmartVacPrep system at 85 ˚C for 12 hrs. The activated samples, **X-dia-4-Co-β** and **X-dia-5-Co-β**, were transferred to the XEMIS-001 instrument and subsequently re-evacuated *in situ* at room temperature. All high-pressure gas sorption studies were performed on the activated phases **X-dia-4-Co-β** and **X-dia-5-Co-β**.

Buoyancy correction was applied using the calculated crystallographic density of the respective open phase **X-dia-4-Co-α** and **X-dia-5-Co-α**.

S10. *In situ* Infrared (IR) spectroscopy

In situ Infrared (IR) spectroscopy methodology*: In situ* IR measurements were performed on a Nicolet 6700 FTIR spectrometer using a liquid N_2-cooled mercury cadmium telluride (MCT-A) detector. The spectrometer is equipped with a vacuum cell that is placed in the main compartment with the sample at the focal point of the infrared beam. To avoid the direct pressing of the **X-dia-4-Co** and **X-dia-5-Co** samples that may cause the damage to the crystalline structures, each sample (~5 mg) was made into slurry form by mixing with a small amount of dimethylacetamide (DMA) and pasted onto the KBr pellet. The powder sample was dried and directly attached unto KBr pellet by heating in the oven at ~60 °C for 5 min, and then transferred into the vacuum cell for activation. The sample was activated by heating up to 85 ºC under vacuum, and then cooled back to 25 °C for the CO_2 adsorption measurements, which were carried out at a steady temperature of 25 °C and variable CO_2 pressure.

DFT methodology for frequency calculation: Density functional theory (DFT) calculations were carried out using the Jaguar electronic structure program in the Schrödinger software suite (Release 2022-2).[10, 11] Structural geometry relaxations were carried out with perturbation to ensure convergence at minimum energy structure, and each was followed by single point vibrational frequency calculation to obtain the IR vibrational spectrum shown in Figure S53. All calculations were performed using the B3LYP hybrid functional[12, 13] and 6-31G** basis set.[14]

S11. CMC Simulations and Binding Site Modelling

The asymmetric nature of the **bimpy** linker creates complexity regarding modelling studies in **X-dia-4-Co**. There are four possible positions for the nitrogen atom in the central aromatic ring. However, due to the symmetry of the crystal structure, there are essentially only two different positions (Figure S3), which generate four possible pore chemistry environments (Figures S4-S5). These four structures were considered for modelling CO_2 binding sites. It is worth noting that these combinations are only valid for a perfectly periodic structure. A much larger number of possible pore environments is possible, due to defects and disorder phenomena. The positional disorder from experimental crystallographic models (CCDC 2181241-2181244) was omitted, and the linker positions were taken as an average of the two possible positions as a starting point for the calculations.

Computational methodology

S11A. Periodic Density Functional Theory (DFT) Calculations

Periodic Density Functional Theory (DFT) calculations were performed using the projected augmented wave (PAW) formalism[15] as implemented in the Vienna Ab Initio Simulation Package (VASP 5.4.4),[16, 17] employing the BEEF-vdW exchange-correlation functional.[18] The atomic positions in open (**α**) and closed (**β**) structures of **X-dia-4-Co** (4 different structures or polymorphs) and **X-dia-5-Co** were optimized at their experimentally refined cell parameters (Table S1), using the conjugate gradient algorithm with force and electronic convergence criteria of 0.01 eV/Å and 10^{-6} eV, a Gaussian smearing of 0.02 eV, an energy cutoff of 550 eV, and Monkhorst-Pack k-point meshes of 2x2x3 for **X-dia-4-Co** 2x3x2 for **X-dia-5-Co**,

respectively.[19] Spin-polarized calculations were performed, and these identified a ferromagnetic coupling between the unpaired electrons of the Co atoms (three unpaired electrons per Co).

Using the Nudged Elastic Band (NEB) as implemented in VASP5.4.4, NEB runs were performed between all closed and open phases with eight images until the atomic forces were below 33 meV/Å for **X-dia-4-Co** and below 48 meV/Å for **X-dia-5-Co** (see Figure 6 in the main document). In the following, the volumes of the different images are labeled V_0 for closed (**β**) and V_9 for open (**α**) structures. The unit cell parameters for eight intermediate images with volumes V_i, $i \subset \{1, 2, \ldots, 8\}$ were found *via* linear interpolation between closed (**β**) and open (**α**) structures and were kept fixed during the NEB runs, for which spring constants of 5 eV/Å^2 (**X-dia-4-Co**) and 0.5 eV/Å^2 (**X-dia-5-Co**) were employed.

Subsequently, canonical Monte Carlo (CMC) simulations were performed to study the CO_2 adsorption on different NEB-optimized images, on the basis of which we identified an initial estimation of the preferential CO_2 binding locations within the optimized closed (**β**) phases and NEB images up to relevant volumes based on our experimental observations (Figures S37 and S44), *i.e.*, below 2110 Å^3 for **X-dia-Co-4** and below 2300 Å^3 for **X-dia-Co-5**; see Section S11B for detailed CMC computational methodology.

Finally, the CO_2 binding sites were optimized using the DFT methodology as described above to qualify the CO_2 adsorption enthalpies and Gibbs free energies. In particular, CO_2 was optimized in the first NEB image for **X-dia-4-Co** ($V_{1,\mathbf{X\text{-}dia\text{-}4}}$ = 2036.27 Å^3, a=14.120 Å, b=17.765 Å, c=8.118 Å, α=β=γ=90º). The CO_2 binding sites were also optimized for the second, third, fourth and fifth NEB image for **X-dia-5-Co** ($V_{2,\mathbf{X\text{-}dia\text{-}5}}$ = 2072.40 Å^3, a=14.923 Å, b=8.367 Å, c=16.598 Å, α=β=γ=90º; $V_{3,\mathbf{X\text{-}dia\text{-}5}}$ = 2149.89 Å^3, a=14.820 Å, b=8.842 Å, c=16.406 Å, α=β=γ=90º; $V_{4,\mathbf{X\text{-}dia\text{-}5}}$ = 2223.40 Å^3, a=14.717 Å, b=8.318 Å, c=16.213 Å, α=β=γ=90º; $V_{5,\mathbf{X\text{-}dia\text{-}5}}$ = 2292.98 Å^3, a=14.6131 Å, b=9.79434 Å, c=16.0208 Å). A partial Hessian vibrational analysis was performed numerically by displacing the atomic coordinates of CO_2 in x, y, and z-directions with ±0.01 Å to verify the binding site as a local-minima on the potential energy surface. Furthermore, the adsorption enthalpies (at 298 K) and zero-point corrected adsorption energies (at 0 K) and adsorption Gibbs free energies (at 298 K and adsorbate pressure of 1 bar) were calculated from the partition functions, using the post-processing python-toolkit TAMkin.[20]

S11B. Canonical Monte Carlo (CMC) Simulations

Canonical Monte Carlo (CMC) simulations were performed to achieve more insight into the CO_2 main binding site locations at 298.15 K in (1x1x2) and (1x2x1) supercells of **X-dia-4-Co** and **X-dia-5-Co**, respectively. Different unit cell volumes were studied, using the framework images with optimized atomic positions from the converged NEB-runs (see Section S11A for DFT methodology). For the CMC simulations, the unit cells and framework atoms were kept fixed at their DFT-optimized positions at a loading of eight CO_2 molecules per supercell (four CO_2 molecules per unit cell), which corresponds to the CO_2 uptake at 10 bar for **X-dia-4-Co** (see Figure S56). This pressure value was chosen in order to compare the two frameworks, since experimental unit cell parameters were available only up to 10 bar for both frameworks (see Tables S6 and S7 and Section S4 for discussion on why unit cell parameters are not available for **X-dia-4-Co** at 35 bar).

For all framework atoms, point charges were determined via the extended charge equilibration (EQeq) method, [22] and the Lennard-Jones (LJ) pair coefficients were taken from the Universal Force Field (UFF)/GenericMOFs using forcefield data available in RASPA ((RASPA-2.0 release, subfolder forcefield/GenericMOFs) (see Table S11). First, the point charges were calculated via the extended charge equilibration (EQeq) method,[22] using RASPA package,[23, 24] followed by CMC simulations in Material Studio.[21] The point charges are supplied in Supplementary CIF-files (**X_dia_4_Co_1st_V1_CO2**.cif, **X_dia_4_Co_2nd_V1_CO2**.cif, **X_dia_4_Co_3rd_V1_CO2**.cif, **X_dia_4_Co_4th_V1_CO2**.cif and **X_dia_5_Co_V5_CO2**.cif). For CO_2, the point charges [e] were taken from the TraPPE force field (see Table S12).

The summary of the CMC algorithm is as follows: the first stage involves loading of the empty framework with the specified number of CO_2 molecules (here 4 molecules per unit cell). This is accomplished by a random series of insertion steps and equilibration moves (only moves that do not change the loading are permitted) until the specified loading has been reached. The main simulation has an equilibration stage and a production stage. In these stages, each step starts with selecting a move type (*e.g.*, translation, rotation, or torsion). In the canonical ensemble, the Metropolis sampling method considered different moves, such as translation (corresponds to translation of the center-of-mass of the selected adsorbate molecule), rotation (rotating the selected adsorbate molecule), regrowth (removing a selected adsorbate molecule from the system and reintroducing it at a random position with random orientation), and conformer (collecting multiple sorbate conformations), with relative probabilities of 1, 1, 0.1 and 1, respectively. After this stage, a random CO_2 molecule is selected and a randomly chosen move is applied. The move is then accepted or rejected, as is typical in a Monte Carlo procedure.

We applied a cutoff distance of 7 Å and truncated the potential using a cubic spline with a spline width of 1 Å. The cutoff value of 7 Å is smaller than half of the shortest simulation box dimension (14.073 to 14.166 for **X-dia-4-Co** and 14.5062 to 14.920 for **X-dia-5-Co**), ensuring that there are no self-interactions between CO_2 molecules. Each CMC simulation included $2{\times}10^6$ loading steps (this specifies the maximum number of steps used when trying to insert all CO_2 molecules into the framework), followed by $2{\times}10^5$ equilibration steps, and finally, 2×10^6 production steps to ensure reasonable ensemble averages.[21] The output of the CMC simulations can be visualized as an isosurface, encompassing the mass-middle points of all successful adsorbate MC moves.

Figures and Tables

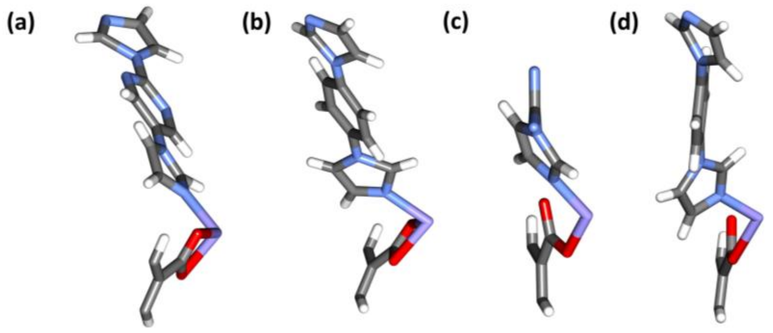

Figure S1. Asymmetric unit for: (a) **X-dia-4-Co-α**, (b) **X-dia-5-Co-α**, (b) **X-dia-4-Co-β** and (d) **X-dia-5-Co-β**.

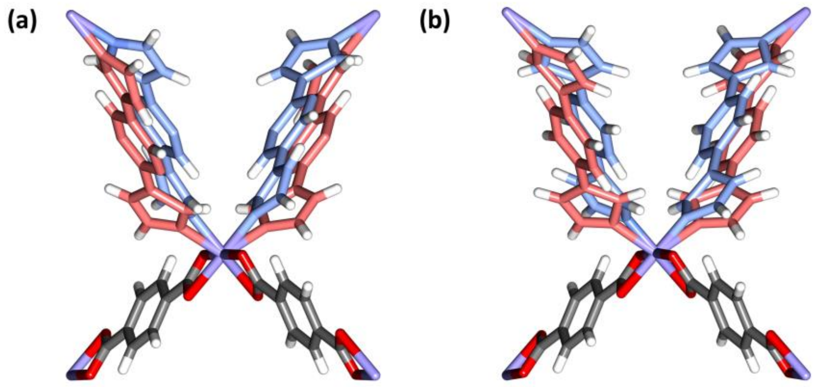

Figure S2. Two-fold positional disorder for: (a) **bimpy** linker in **X-dia-4-Co-α** and (b) **bimbz** linker in **X-dia-5-Co-α**.

As discussed in Section S2, the crystal structures of **X-dia-4-Co-α** and **X-dia-5-Co-α** show positional disorder. Figure S2 portrays the 2-fold disorder of the **bimpy** and **bimbz** linkers. Since the two positions of the linkers are related through a center of inversion, only one configuration is shown in the rest of the Figures. Additionally, **X-dia-4-Co-α** and **X-dia-4-Co-β** show substitutional disorder (Section S2). In the case of **X-dia-4-Co-α** the linker is found in two positions, so each position is modelled to contain two nitrogen and two carbon atoms in the central ring, to account for substitutional disorder. In the case of **X-dia-4-Co-β** the linker does not show positional disorder, so all four positions of the central pyridine ring are occupied by both carbon and nitrogen atoms. Therefore, no hydrogen atoms are shown in any of the graphics for the central ring in this structure.

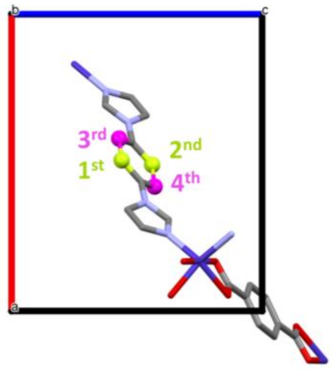

Figure S3. Possible positions for the nitrogen atom in the central pyridine ring in **X-dia-4-Co**.

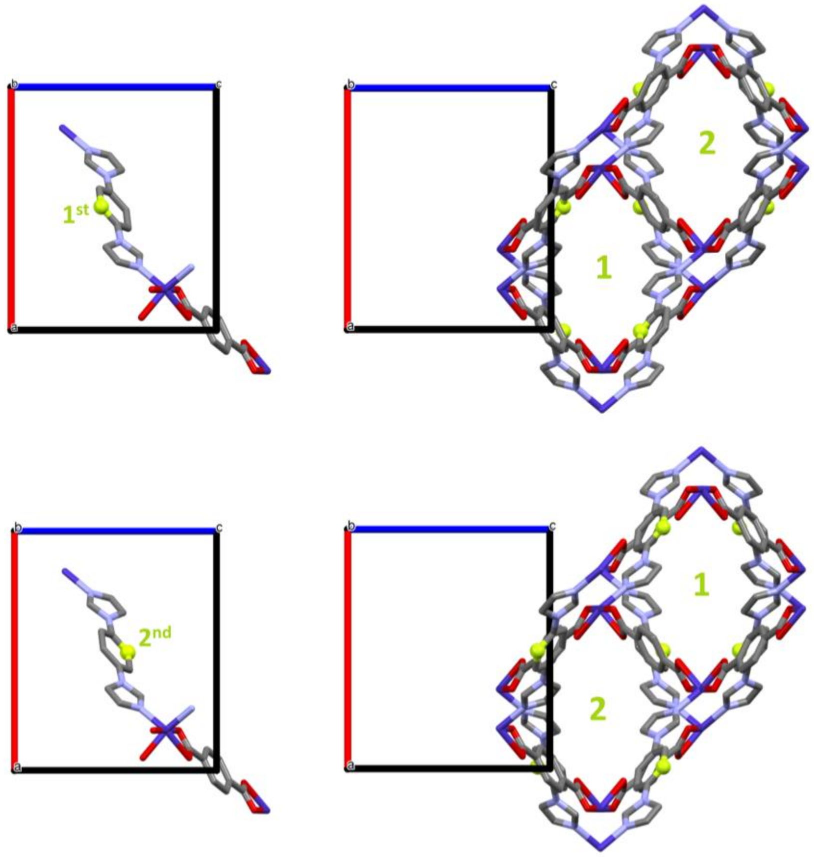

Figure S4. Positions **X-dia-4-Co-1st** and **X-dia-4-Co-2nd** for the nitrogen atom of the **bimpy** linker in **X-dia-4-Co**. Positions **X-dia-4-Co-1st** and **X-dia-4-Co-2nd** generate the same combination of two different pore chemistry environments (environments 1 and 2).

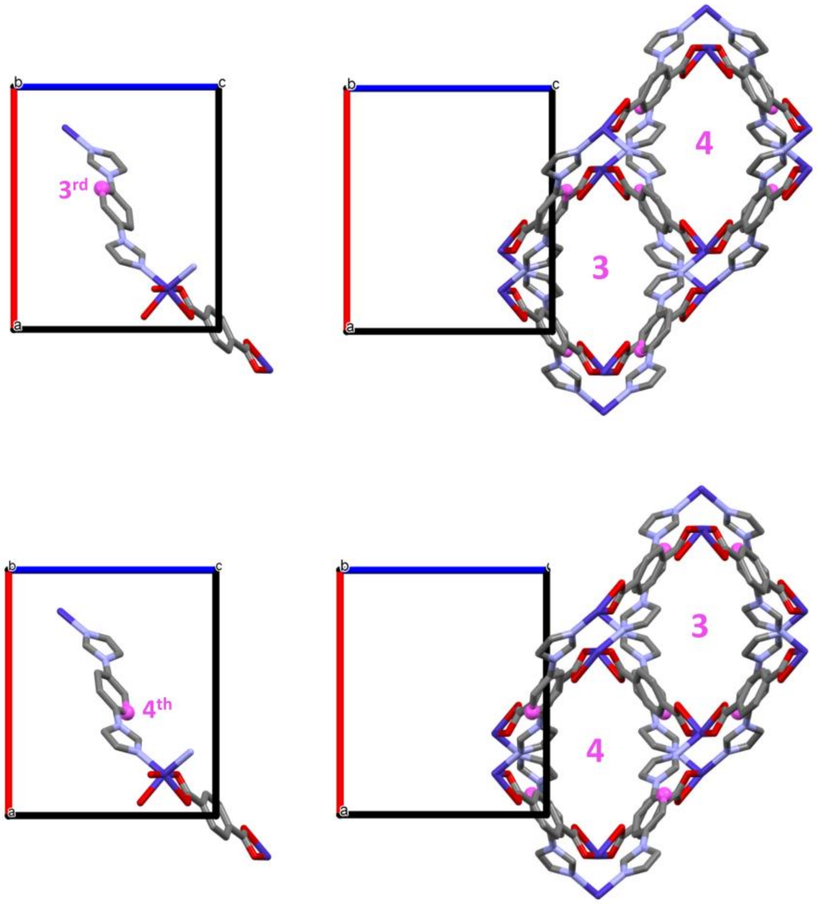

Figure S5. Positions **X-dia-4-Co-3rd** and **X-dia-4-Co-4th** for the nitrogen atom of the **bimpy** linker in **X-dia-4-Co**. Positions **X-dia-4-Co-3rd** and **X-dia-4-Co-4th** generate the same combination of two different pore chemistry environments (environments 3 and 4).

Table S1. Crystallographic data and refinement parameters for **X-dia-4-Co** and **X-dia-5-Co**.

**Compound**	**X-dia-4-Co-α**	**X-dia-4-Co-β**	**X-dia-5-Co-α**	**X-dia-5-Co-β**
Formula	$C_{18}H_{12}CoN_6O_4$	$C_{18}H_{10}CoN_6O_4$	$C_{20}H_{14}CoN_4O_4$	$C_{20}H_{14}CoN_4O_4$
Formula weight	435.27	433.25	433.28	433.28
Temperature (K)	100(2)	100(2)	100(2)	150(2)
Crystal system	Orthorhombic	Orthorhombic	Orthorhombic	Orthorhombic
Space group	*Pnna*	*Pnna*	*Pnna*	$Pna2_1$
a (Å)	13.7467(4)	14.1665(11)	14.1993(6)	15.1303(18)
b (Å)	15.6394(5)	18.0309(16)	15.2509(6)	7.4147(9)
c (Å)	11.6109(4)	7.6811(8)	11.6980(5)	16.983(2)
α (°)	90	90	90	90
β (°)	90	90	90	90
γ (°)	90	90	90	90
V ($Å^3$)	2496.23(14)	1962.0(3)	2533.23(18)	1905.3(4)
Z	4	4	4	4
D_c ($g{\cdot}cm^{-3}$)	1.158	1.467	1.136	1.510
μ (mm^{-1})	5.653	7.192	5.545	0.936
R_{int}	0.1602	0.1750	0.1234	0.1862
GOF	1.250	1.064	1.540	1.119
R_1 [I > 2σ (I)]	0.1320	0.1071	0.1420	0.1340
WR_2 [all data]	0.3476	0.3294	0.3936	0.4865
Diff peak / hole (e $Å^{-3}$)	1.469 / -0.577	0.414 / -0.712	1.516 / -0.602	0.841 / -0.589
No. CCDC	2181241	2181242	2181244	2181243

$R_1 = \sum||F_o| - |F_c||/\sum|F_o|$. $wR_2 = [\sum w(F_o^2 - F_c^2)^2 / \sum w(F_o^2)^2]^{1/2}$

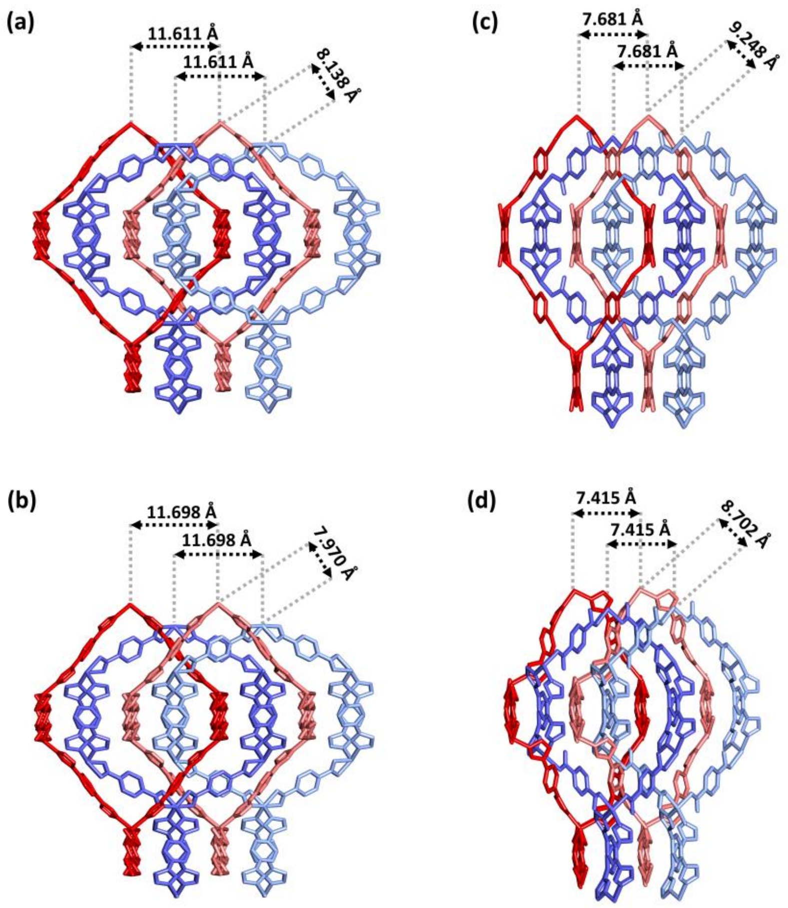

Figure S6. Comparative analysis of the 4-fold interpenetrated diamondoid nets in: (a) **X-dia-4-Co-α**, (b) **X-dia-5-Co-α**, (c) **X-dia-4-Co-β** and (d) **X-dia-5-Co-β.**

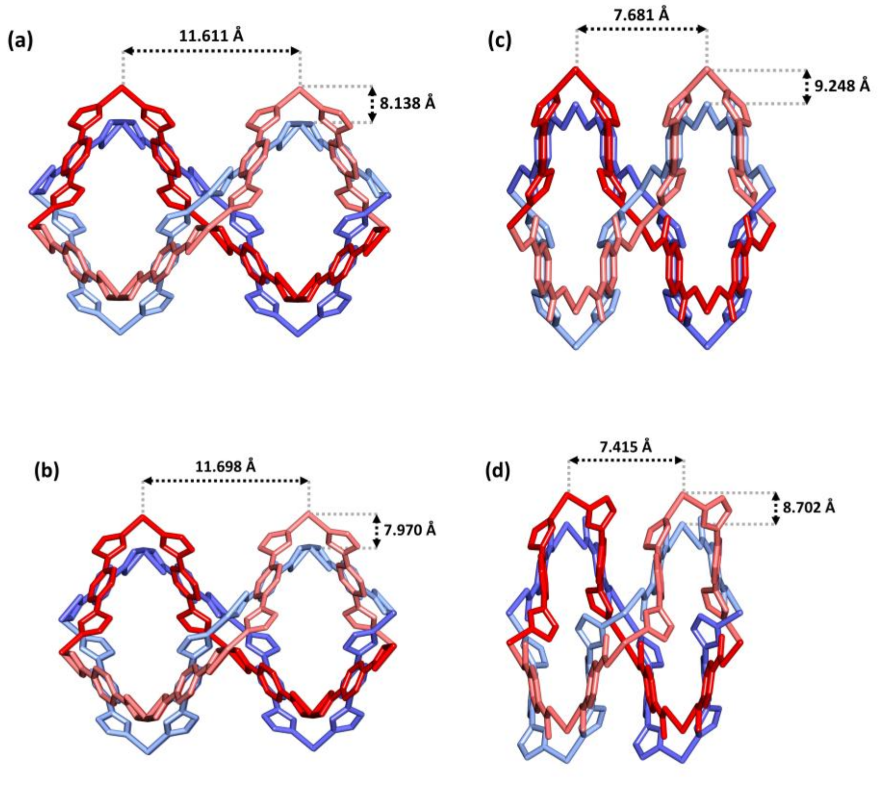

Figure S7. Comparative analysis of the 4-fold interpenetrated diamondoid nets in: (a) **X-dia-4-Co-α**, (b) **X-dia-5-Co-α**, (c) **X-dia-4-Co-β** and (d) **X-dia-5-Co-β.**

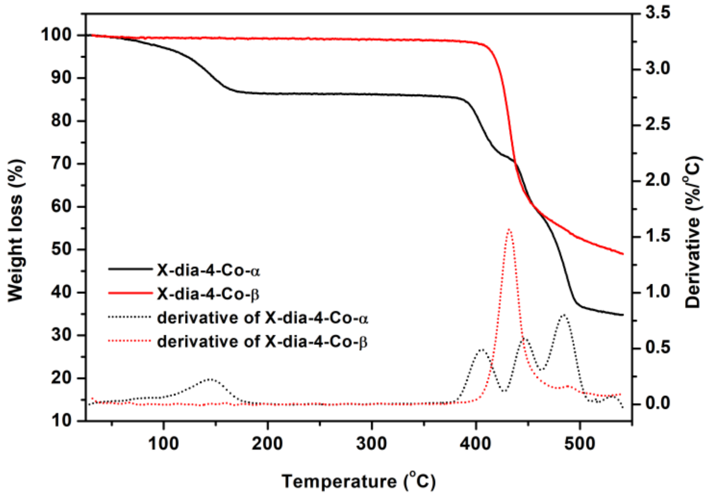

Figure S8. TG curves of **X-dia-4-Co-α** (black) and **X-dia-4-Co-β** (red) under N_2 environment.

As shown in Figure S8, **X-dia-4-Co-α** displayed a weight loss of 15.0% completed at 170 °C, corresponding to the release of one DMA molecule per formula unit (calc. 16.7%). **X-dia-4-Co-β** showed no weight loss up to 400 °C, indicating that no guest molecules were found in this phase.

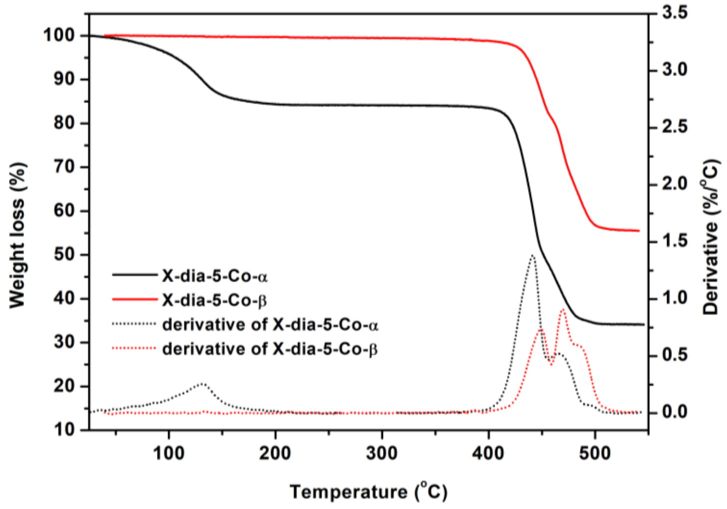

Figure S9. TG curves of **X-dia-5-Co-α** (black) and **X-dia-5-Co-β** (red) under N_2 environment.

As shown in Figure S9, **X-dia-5-Co-α** displayed a weight loss of 16.0% completed at 150 °C, corresponding to the release of one DMA molecule per formula unit (calc. 16.7%). **X-dia-5-Co-β** showed no weight loss up to 400 °C, indicating that no guest molecules were found in this phase.

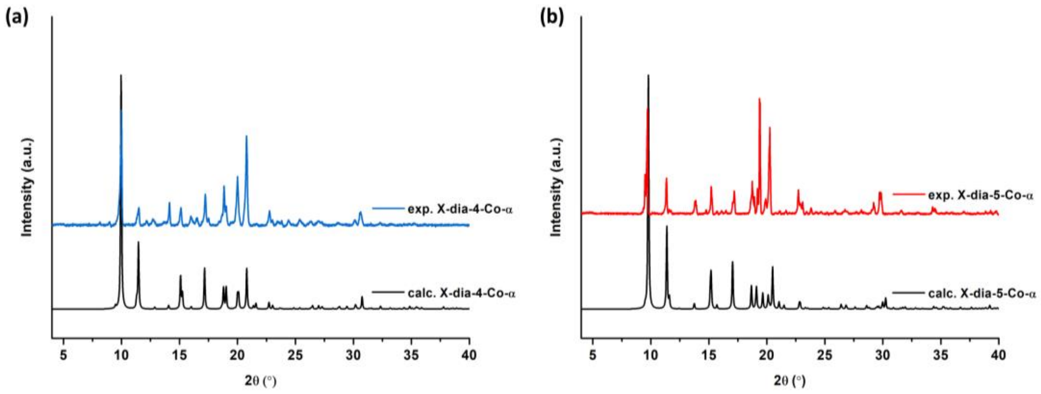

Figure S10. PXRD patterns for the open phases: (a) **X-dia-4-Co-α** and (b) **X-dia-5-Co-α**.

Figure S10 demonstrated a match between calculated and experimental powder patterns for **X-dia-4-Co-α** and **X-dia-5-Co-α**, which confirmed phase purity.

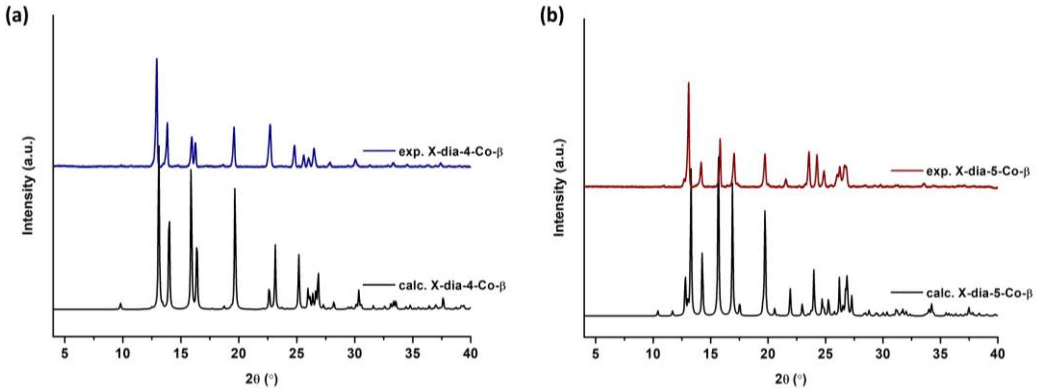

Figure S11. PXRD patterns for the closed phases: (a) **X-dia-4-Co-β** and (b) **X-dia-5-Co-β**.

Figure S11 demonstrated that calculated and experimental PXRD patterns were in good agreement. Distinctive peaks for the two compounds highlighted that the two closed phases are structurally different.

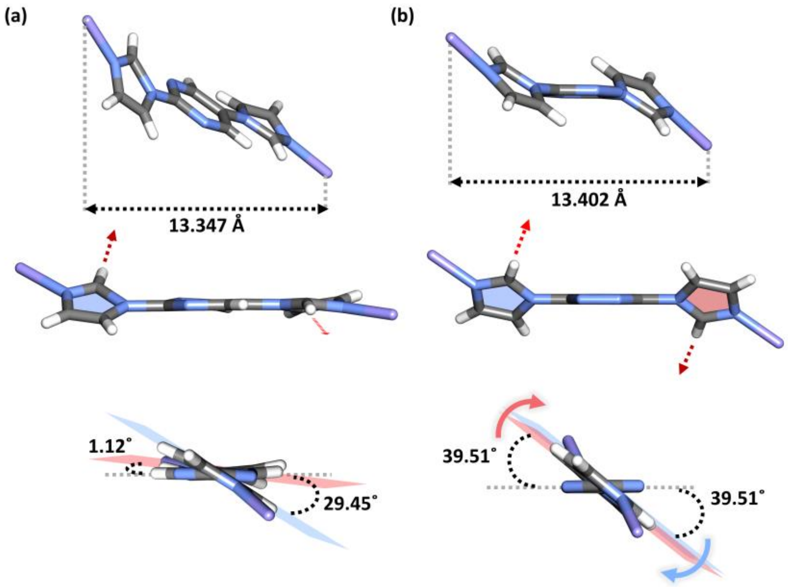

Figure S12. Comparative analysis of the **bimpy** linker conformations along different directions, in the two isolated phases of **X-dia-4-Co**: (a) **X-dia-4-Co-α** and (b) **X-dia-4-Co-β**.

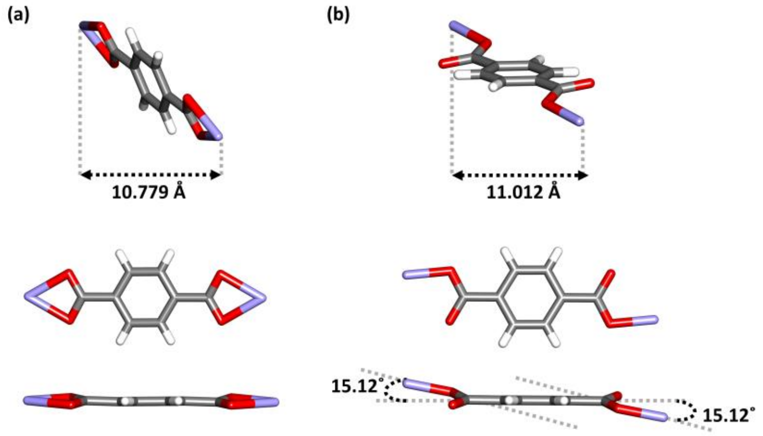

Figure S13. Comparative analysis of the **bdc^{2-}** linker conformations along different directions, in the two isolated phases of **X-dia-4-Co**: (a) **X-dia-4-Co-α** and (b) **X-dia-4-Co-β**.

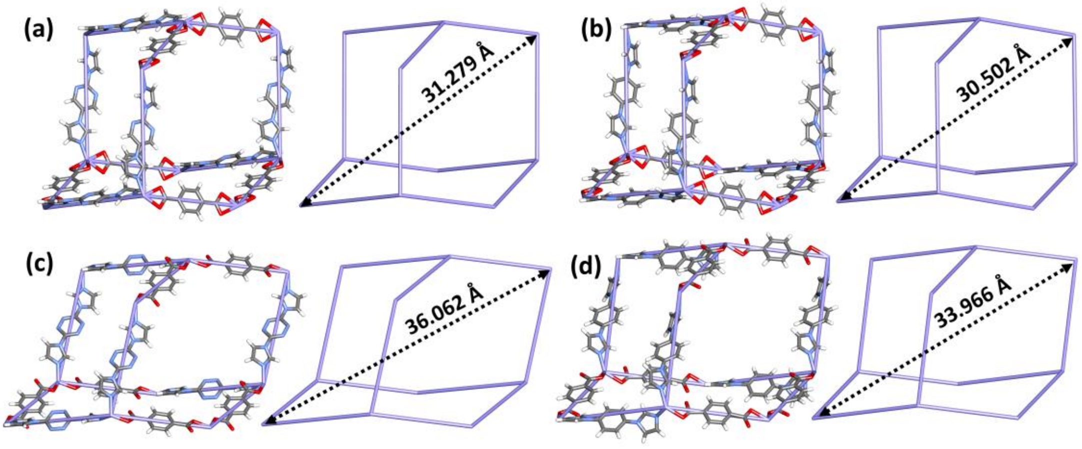

Figure S14. Single adamantoid cage representations in: (a) **X-dia-4-Co-α**, (b) **X-dia-5-Co-α**, (c) **X-dia-4-Co-β** and (d) **X-dia-5-Co-β**.

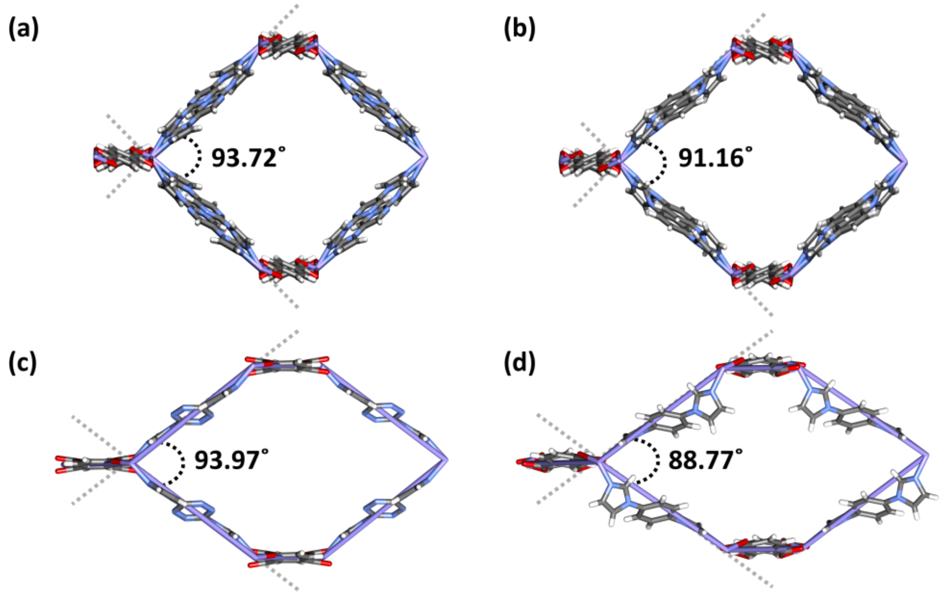

Figure S15. Single adamantoid cage representations in: (a) **X-dia-4-Co-α** (disordered), (b) **X-dia-5-Co-α** (disordered), (c) **X-dia-4-Co-β** and (d) **X-dia-5-Co-β**.

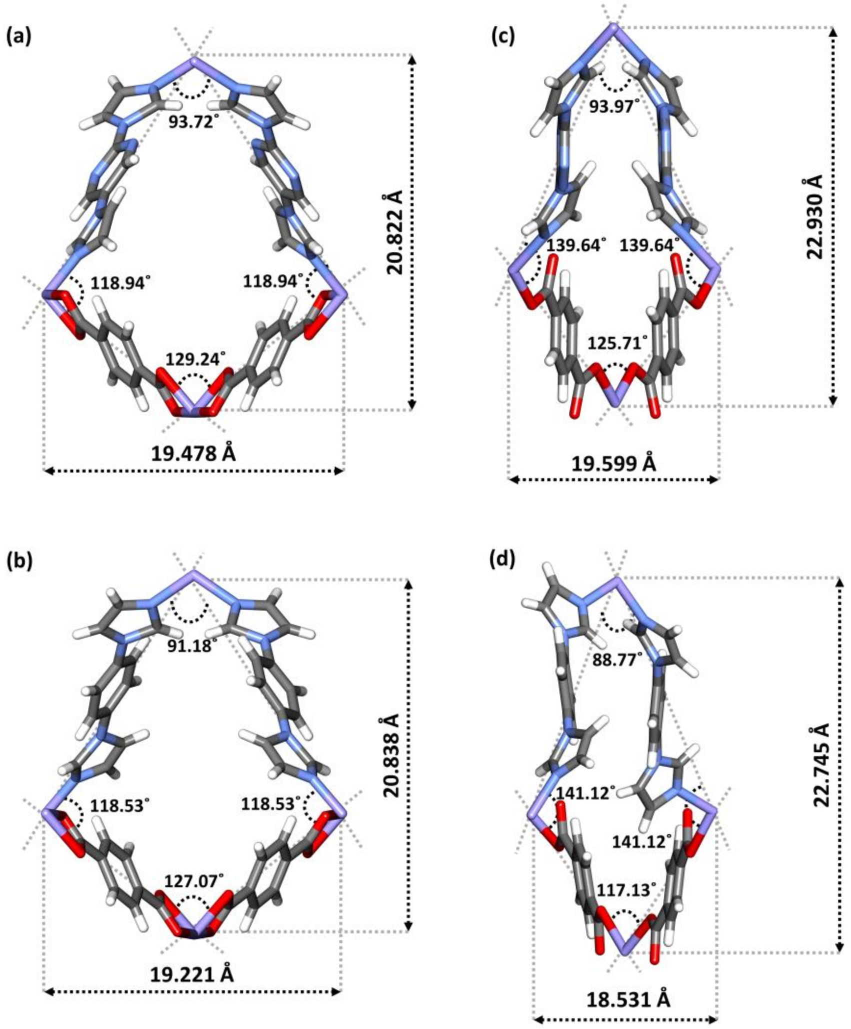

Figure S16. Single net representations in: (a) **X-dia-4-Co-α**, (b) **X-dia-5-Co-α**, (c) **X-dia-4-Co-β**, and (d) **X-dia-5-Co-β**.

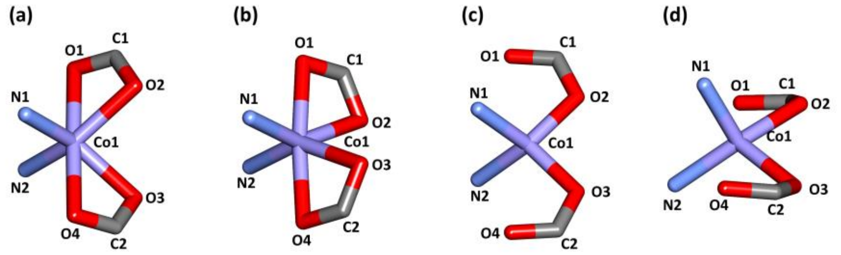

Figure S17. Coordination environment around the Co^{2+} center in: (a) **X-dia-4-Co-α**, (b) **X-dia-5-Co-α**, (c) **X-dia-4-Co-β** and (d) **X-dia-5-Co-β**.

Table S2. List of angles around the Co^{2+} center in **X-dia-4-Co** and **X-dia-5-Co**.

Angle	X-dia-4-Co-α (˚)	X-dia-5-Co-α (˚)	X-dia-4-Co-β (˚)	X-dia-5-Co-β (˚)
∠ N1-Co1-N2	82.76	85.58	98.49	100.95
∠ N1-Co1-O1	93.94*	95.21*	87.52*	99.48
∠ N2-Co1-O4				86.49
∠ N1-Co1-O2	105.38*	101.96*	121.25*	102.58
∠ N2-Co1-O3				113.65
∠ O1-Co1-O2	59.93*	60.92*	51.29*	51.66
∠ O3-Co1-O4				54.02
∠ O2-Co1-O3	86.10	86.73	95.11	87.16

Table S2 shows that some angles in **X-dia-4-Co-α**, **X-dia-5-Co-α** and **X-dia-4-Co-β** are symmetry-related (marked with an asterisk), and, therefore, identical, due to the *Pnna* space group. However, all angles in **X-dia-5-Co-β** are unique, which accounts for the $Pna2_1$ space group.

Table S3. List of distances around the Co^{2+} center in **X-dia-4-Co** and **X-dia-5-Co**.

Distance	X-dia-4-Co-α (Å)	X-dia-5-Co-α (Å)	X-dia-4-Co-β (Å)	X-dia-5-Co-β (Å)
N1-Co1	2.020	1.870	2.012	1.945
N2-Co1				2.042
O1-Co1	2.179	2.145	2.823	2.503
O4-Co1				2.719
O2-Co1	2.173	2.162	1.958	2.094
O3-Co1				2.092
C1-Co1	2.497	2.478	2.729	2.544
C2-Co1				2.660

Table S3 shows that all distances in **X-dia-5-Co-β** are unique due to the $Pna2_1$ space group, while the distances in the rest of the phases are identical in sets of two, due to the *Pnna* space group.

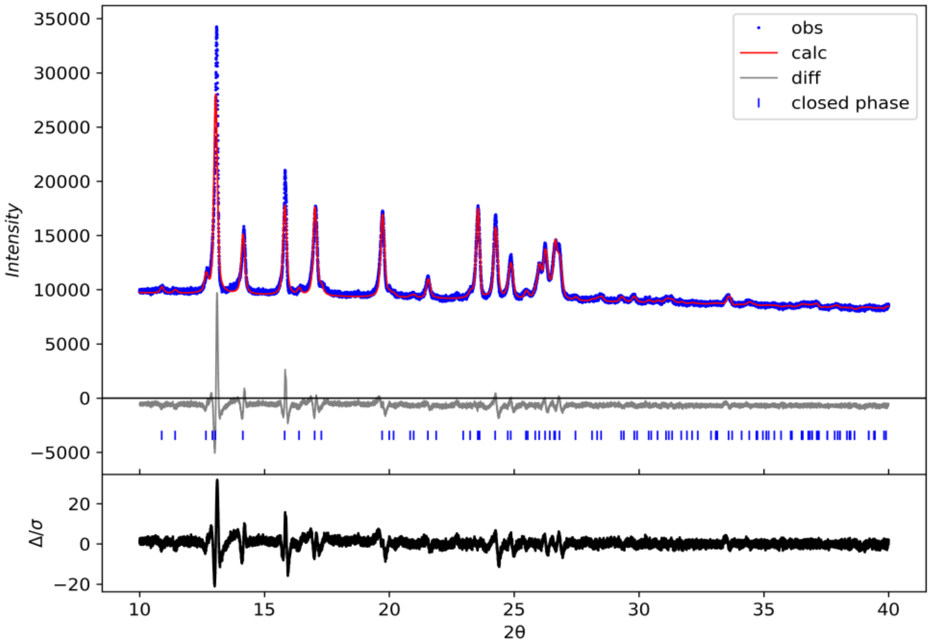

Figure S18. Pawley profile fit of PXRD pattern for **X-dia-5-Co-β**.

Table S4. Comparison of unit cell parameters of **X-dia-5-Co-β** obtained by SCXRD and Pawley profile fit of the experimental PXRD pattern.

	**Space group**	**a (Å)**	**b (Å)**	**c (Å)**	**α = β = γ (°)**	**V (Å^3)**
Crystal structure of **X-dia-5-Co-β** (100 K)	*Pna*2$_1$	15.1303(18)	7.4147(9)	16.983(2)	90	1905.3(4)
Pawley profile fit of PXRD pattern (298 K)	*Pna*2$_1$	15.4796(11)	7.55079(17)	16.2363(12)	90	1897.75(10)

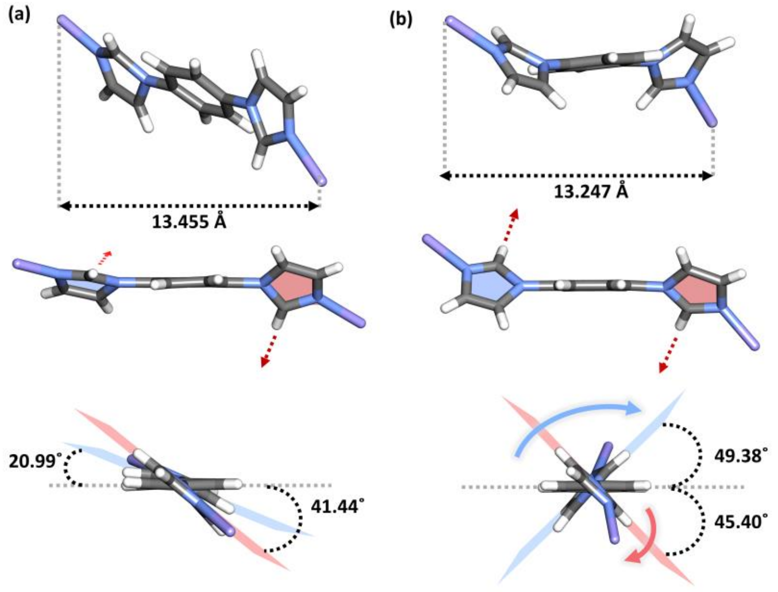

Figure S19. Comparative analysis of the **bimbz** linker conformations along different directions, in the two isolated phases of **X-dia-5-Co**: (a) **X-dia-5-Co-α** and (b) **X-dia-5-Co-β**.

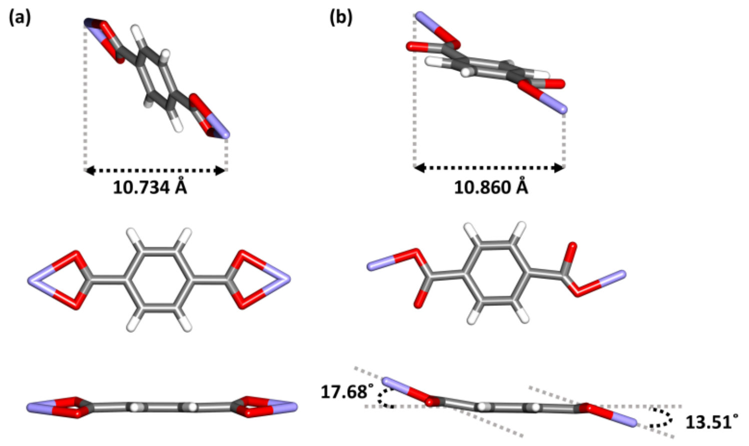

Figure S20. Comparative analysis of the **bdc**$^{2-}$ linker conformations along different directions, in the two isolated phases of **X-dia-5-Co**: (a) **X-dia-5-Co-α** and (b) **X-dia-5-Co-β**.

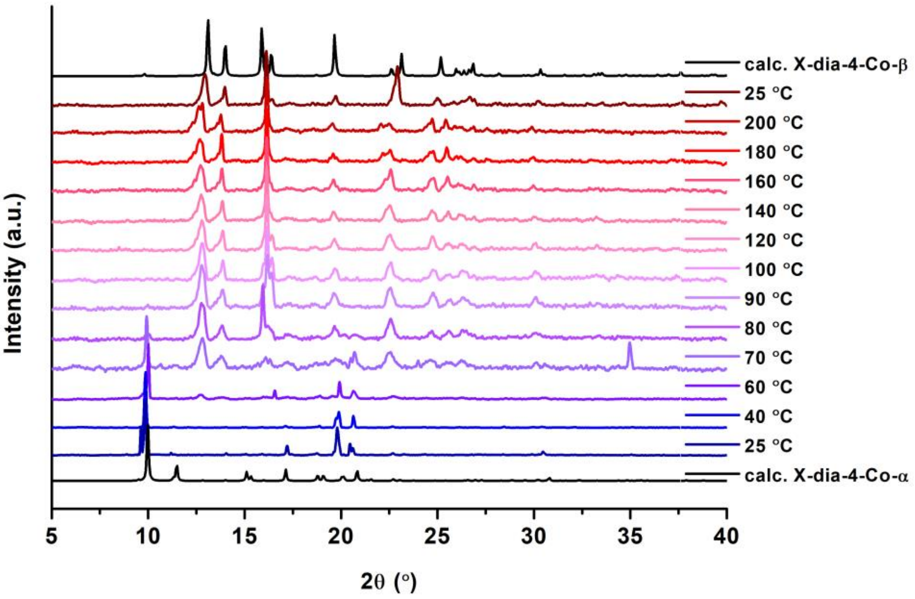

Figure S21. *In situ* variable-temperature PXRD patterns of **X-dia-4-Co-α**.

According to Figure S21, **X-dia-4-Co-α** started to undergo a phase transformation at 70 °C. The phase change to **X-dia-4-Co-β** was completed at 90 °C. This is in agreement with DSC data (Figure S23).

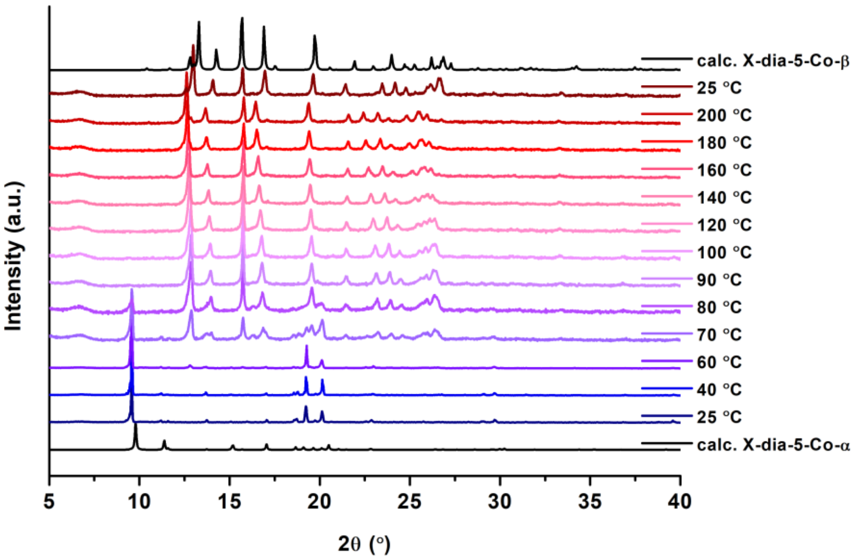

Figure S22. *In situ* variable-temperature PXRD patterns of **X-dia-5-Co-α.**

According to Figure S22, **X-dia-5-Co-α** started to undergo a phase transformation at 70 °C. The phase change to **X-dia-5-Co-β** was completed at 90 °C. This is in agreement with DSC data (Figure S24).

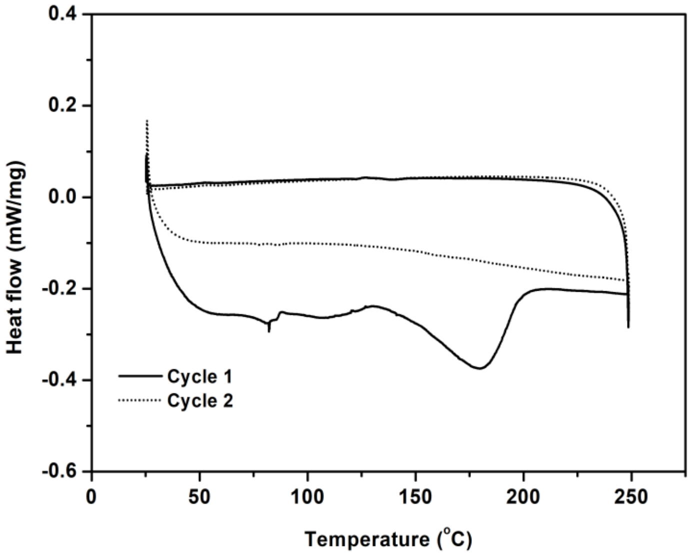

Figure S23. DSC profile of **X-dia-4-Co-α** for two consecutive cycles.

The first DSC cycle in Figure S23 (solid black line) indicated that the phase change occurred around 80 °C, which is consistent with the weight loss from the TG curve and VT-PXRD data (Figures S8 and S21). The absence of the phase change peak in the second cycle (dotted black line) showed that the resulting **X-dia-4-Co-β** remained stable after conversion.

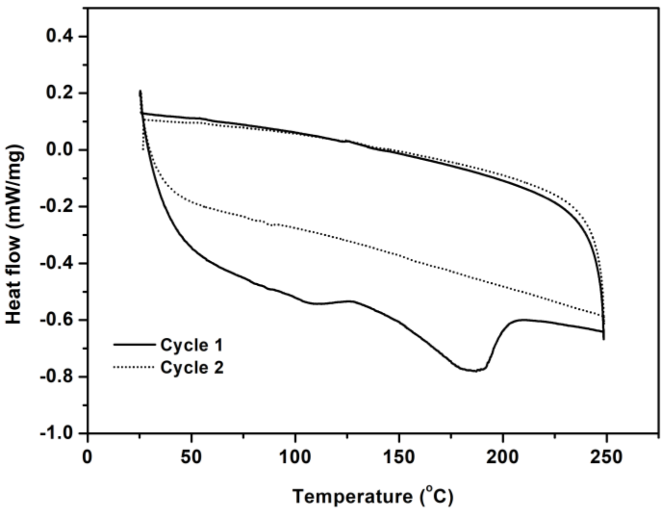

Figure S24. DSC profile of **X-dia-5-Co-α** for two consecutive cycles.

The first DSC cycle in Figure S24 (solid black line) indicated that the phase change occurred around 100 °C, which is consistent with the weight loss from the TG curve and VT-PXRD data (Figures S9 and S22). The absence of the phase change peak in the second cycle (dotted black line) showed that the resulting **X-dia-5-Co-β** remained stable after conversion.

Table S5. Langmuir fitting for **X-dia-4-Co** and **X-dia-5-Co**.

	X-dia-4-Co	X-dia-5-Co
**Langmuir surface area (m²/g)**	499.6260 ± 0.3953	482.5427 ± 0.4546
**Slope (g/cm³ STP)**	0.009142 ± 0.000007	0.009466 ± 0.000009
**Y-intercept (g/cm³ STP·mmHg)**	0.097 ± 0.003	0.032 ± 0.003
**b (1/mmHg)**	0.094002	0.292423
**Qm (cm³/g STP)**	109.3867	105.6465
**Correlation coefficient**	0.999994	0.999987
**Molecular cross-sectional area (nm²)**	0.1700	0.1700

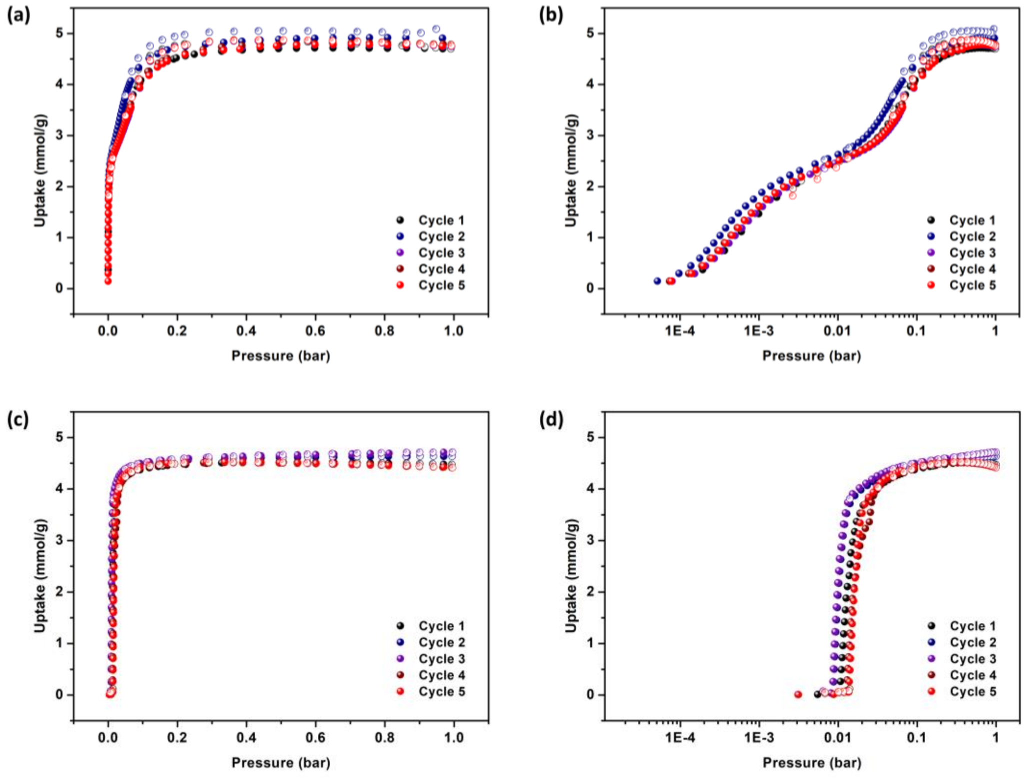

Figure S25. Cycling experiments for CO_2 at 195 K. Five consecutive cycles for **X-dia-4-Co** (a) and **X-dia-5-Co** (c). Log plots for five consecutive cycles for **X-dia-4-Co** (b) and **X-dia-5-Co** (d). Adsorption: closed sphere; desorption: open sphere.

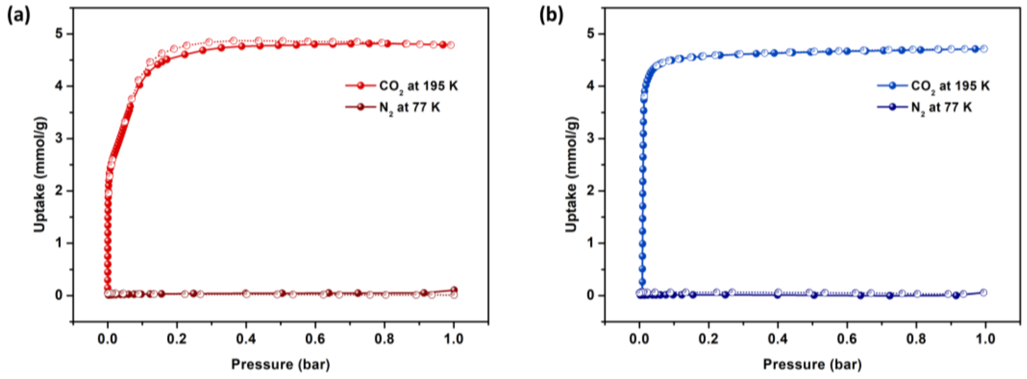

Figure S26. Low-pressure CO_2 and N_2 sorption isotherms for: (a) **X-dia-4-Co** and (b) **X-dia-5-Co**. Adsorption: closed sphere; desorption: open sphere.

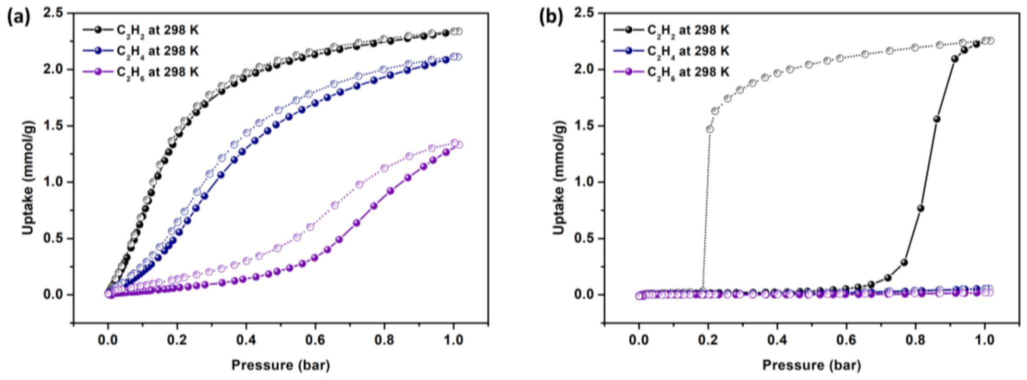

Figure S27. Low-pressure C_2H_2, C_2H_4 and C_2H_6 at 298 K sorption isotherms for: (a) **X-dia-4-Co** and (b) **X-dia-5-Co**. Adsorption: closed sphere; desorption: open sphere.

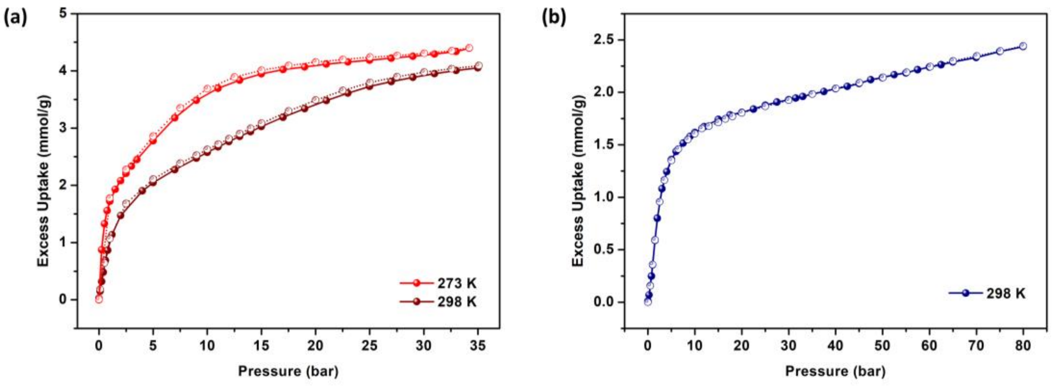

Figure S28. (a) High-pressure CO_2 sorption isotherms for **X-dia-4-Co** at 273 K (red) and 298 K (maroon). (b) High-pressure CH_4 sorption isotherm for **X-dia-4-Co** at 298 K. Adsorption: closed sphere; desorption: open sphere.

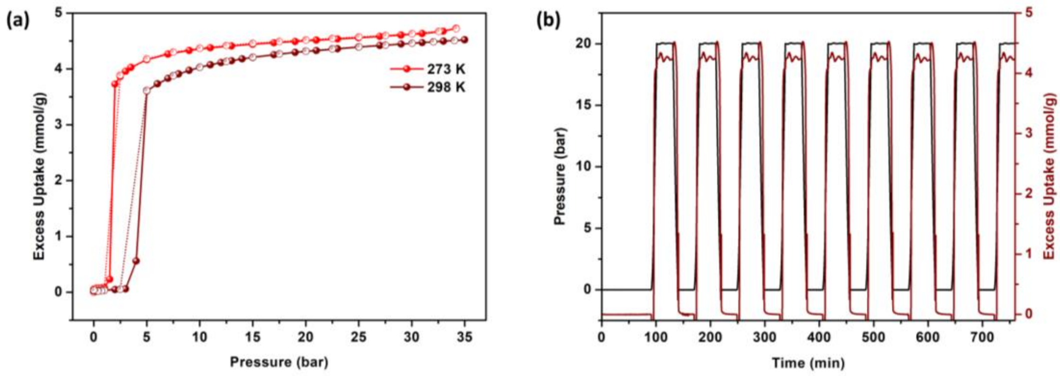

Figure S29. (a) High-pressure CO_2 sorption isotherms for **X-dia-5-Co** at 273 K (red) and 298 K (maroon). Adsorption: closed sphere; desorption: open sphere. (b) Cycling experiment for **X-dia-5-Co** under CO_2 at 298 K between 0 and 20 bar.

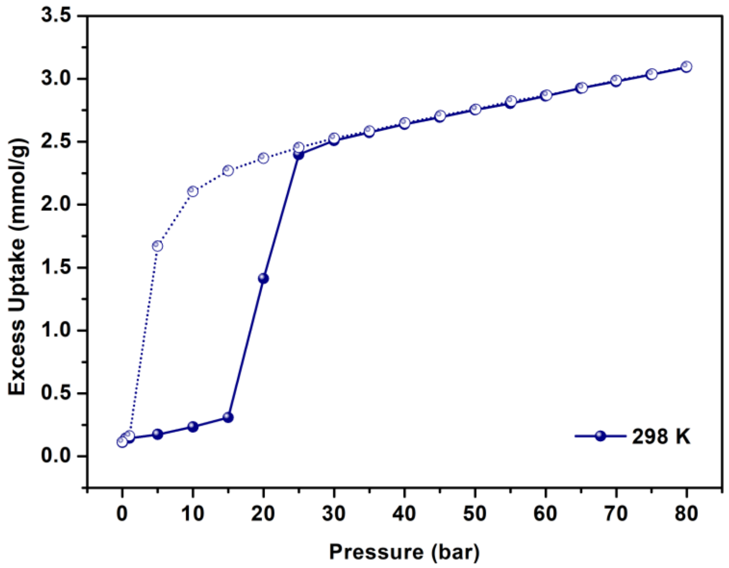

Figure S30. High-pressure CH_4 sorption isotherm for **X-dia-5-Co** at 298 K. Adsorption: closed sphere; desorption: open sphere.

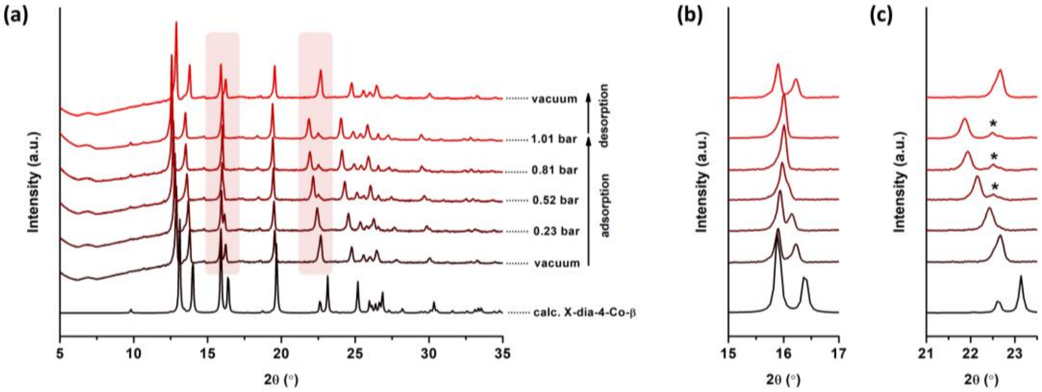

Figure S31. (a) *In situ* PXRD patterns for **X-dia-4-Co** in the presence of CO_2, collected at 298 K in the pressure region of 0 to 1 bar. Red rectangles highlight the regions with major peak changes. Magnified *in situ* PXRD patterns in the 2θ range: (b) 15° to 17° and (c) 21° to 23.5°.

As shown in Figure S31, PXRD patterns of **X-dia-4-Co-β** changed upon exposure to CO_2 at 298 K. Figure S31b shows that two peaks at 2θ ≈16° merged into one with subsequent CO_2 loading. In addition, Figure S31c shows that the peak at 2θ ≈ 22.5° shifted to lower 2θ values, while an additional peak appeared at 0.52 bar (marked with an asterisk).

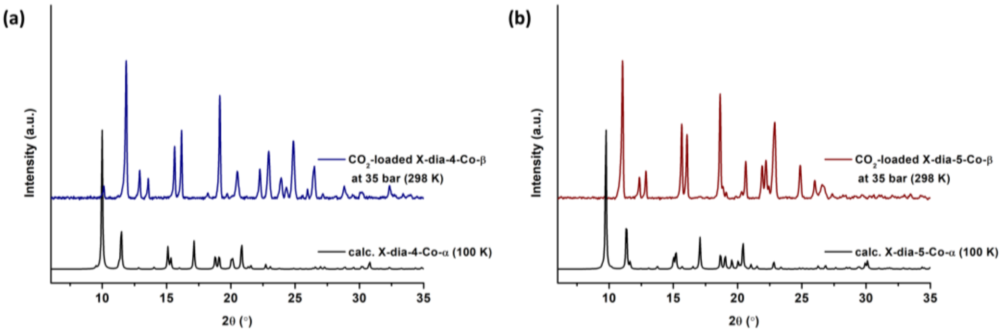

Figure S32. The *in situ* CO_2-loaded PXRD pattern of **β** phases at 35 bar and 298 K differ from the calculated PXRD pattern of the as-synthesised **α** phases: (a) **X-dia-4-Co** and (b) **X-dia-5-Co**.

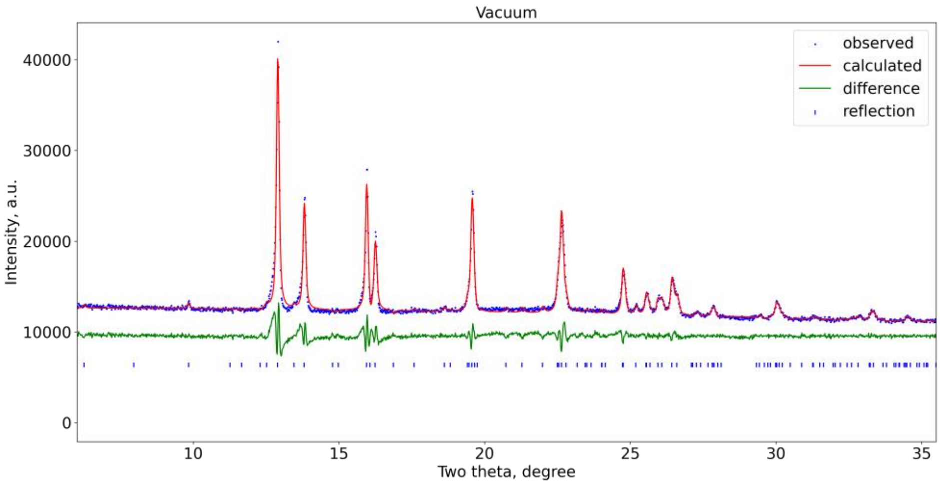

Figure S33. Profile fit of *in situ* PXRD pattern of **X-dia-4-Co-β** at 0 bar and 298 K.

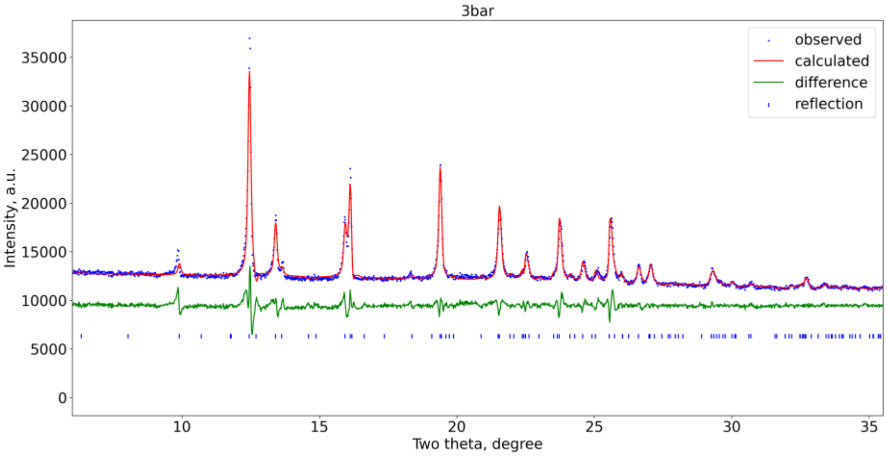

Figure S34. Profile fit of *in situ* CO_2-loaded PXRD pattern of **X-dia-4-Co-β** at 3 bar and 298 K.

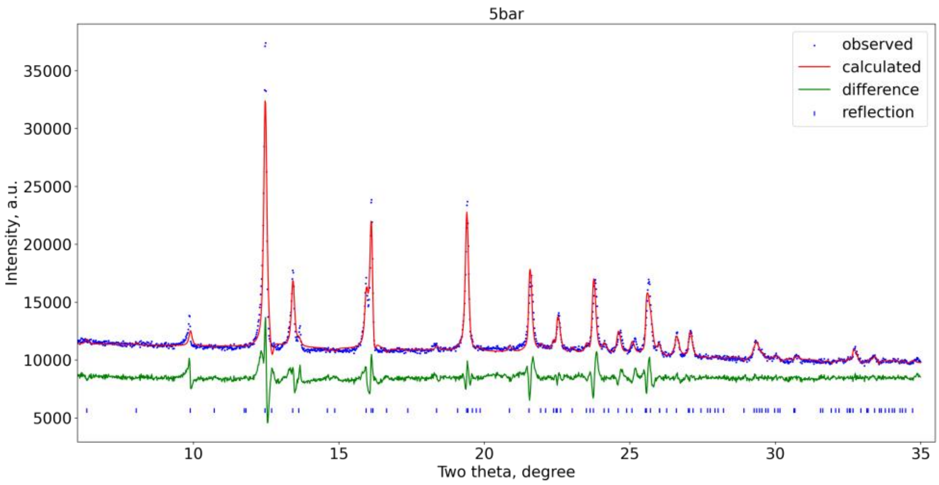

Figure S35. Profile fit of *in situ* CO_2-loaded PXRD pattern of **X-dia-4-Co-β** at 5 bar and 298 K.

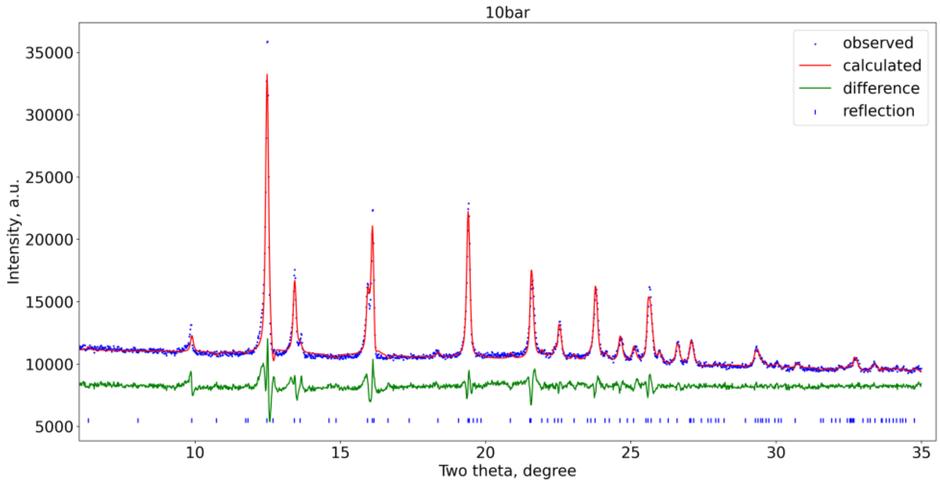

Figure S36. Profile fit of *in situ* CO_2-loaded PXRD pattern of **X-dia-4-Co-β** at 10 bar and 298 K.

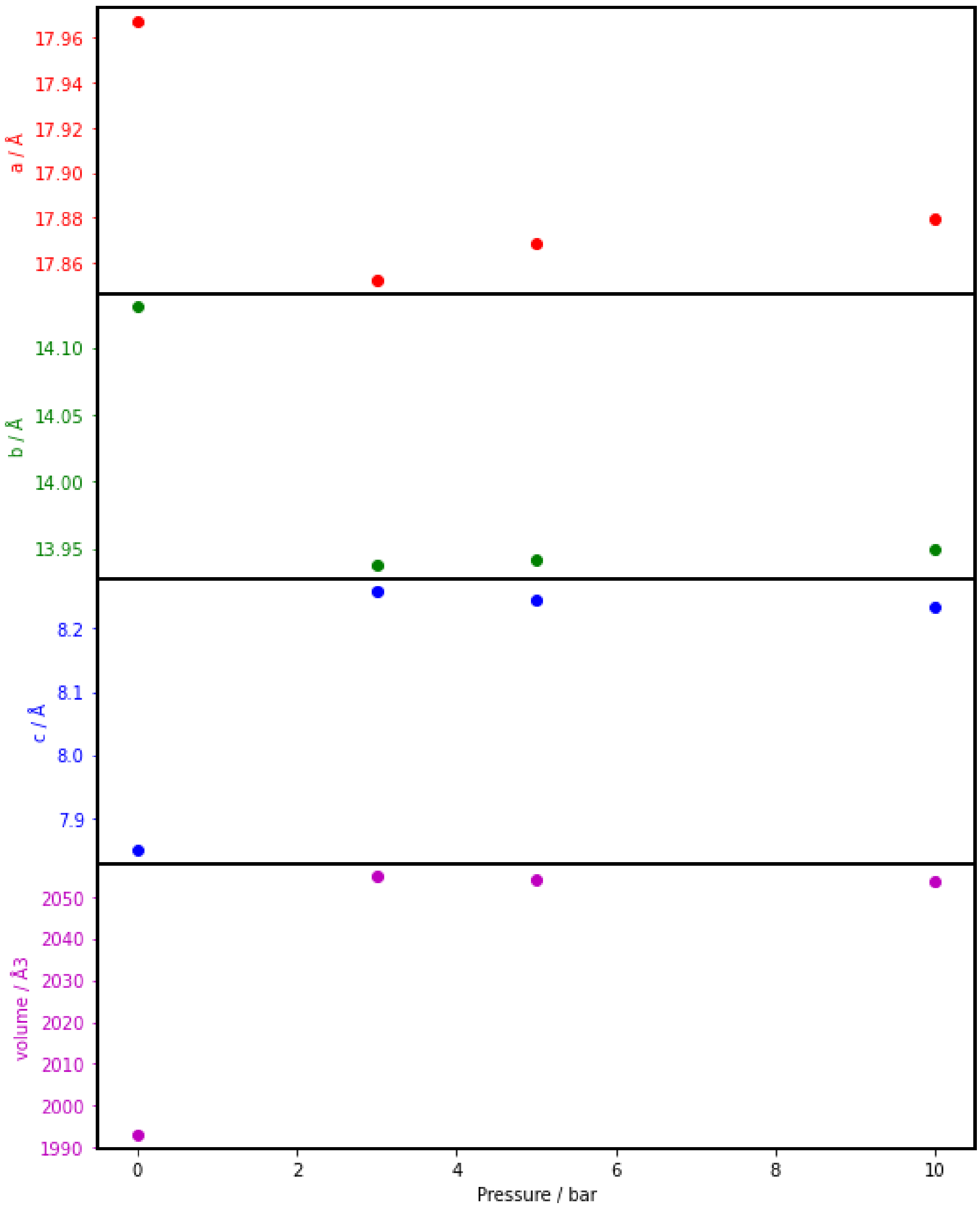

Figure S37. Unit cell parameters of **X-dia-4-Co-β** obtained from Pawley profile fits of high-pressure *in situ* CO_2-loaded PXRD patterns at 298 K.

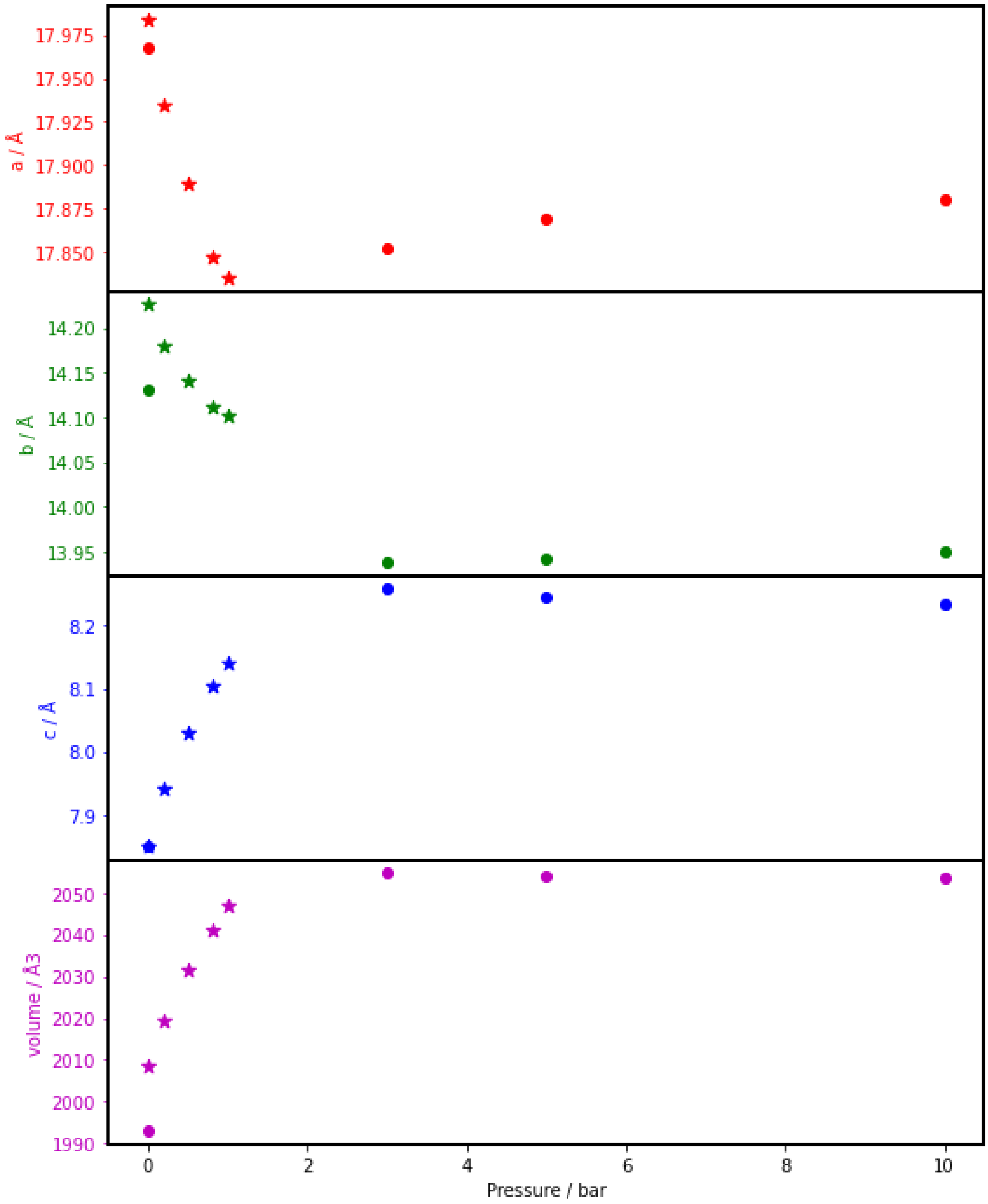

Figure S38. Unit cell parameters of **X-dia-4-Co-β** obtained from Pawley profile fits of low- and high-pressure *in situ* CO_2-loaded PXRD patterns at 298 K (stars: obtained from low-pressure region of 0 to 1 bar; spheres: obtained from high-pressure region of 1 to 10 bar – see Section S6 for experimental details).

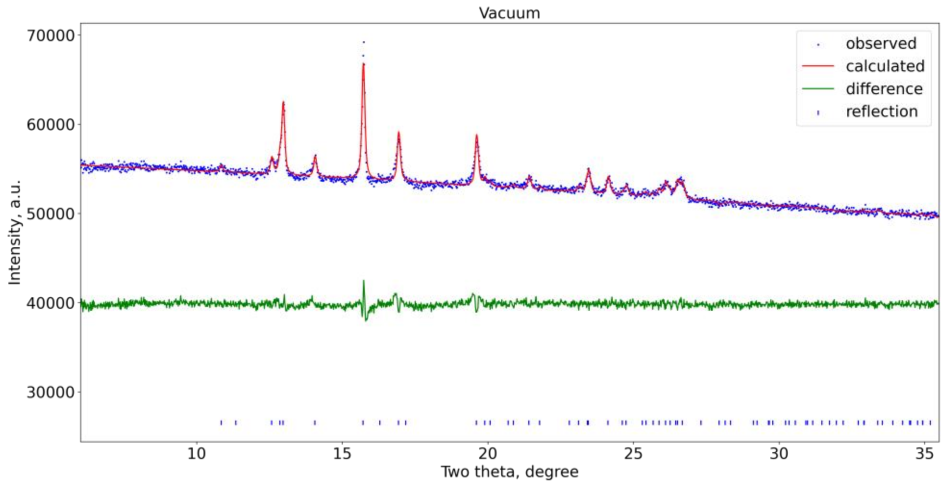

Figure S39. Profile fit of *in situ* PXRD pattern of **X-dia-5-Co-β** at 0 bar and 298 K.

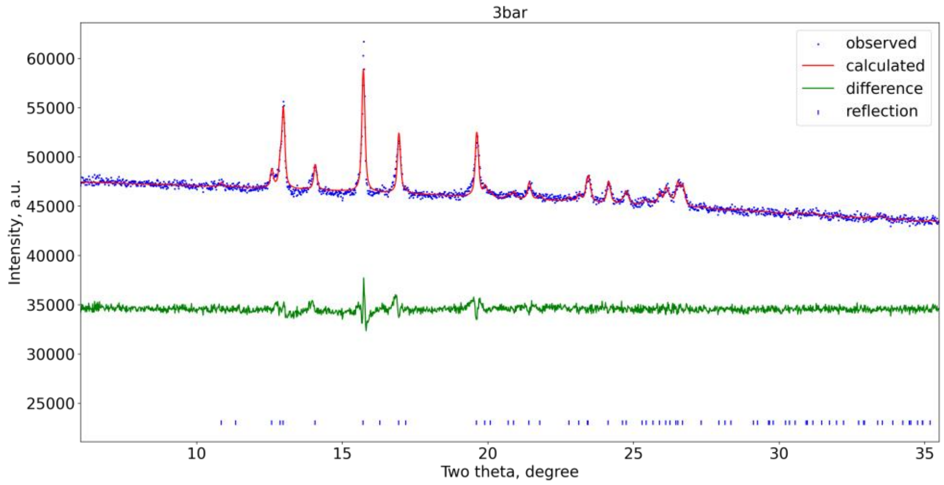

Figure S40. Profile fit of *in situ* CO_2-loaded PXRD pattern of **X-dia-5-Co-β** at 3 bar and 298 K.

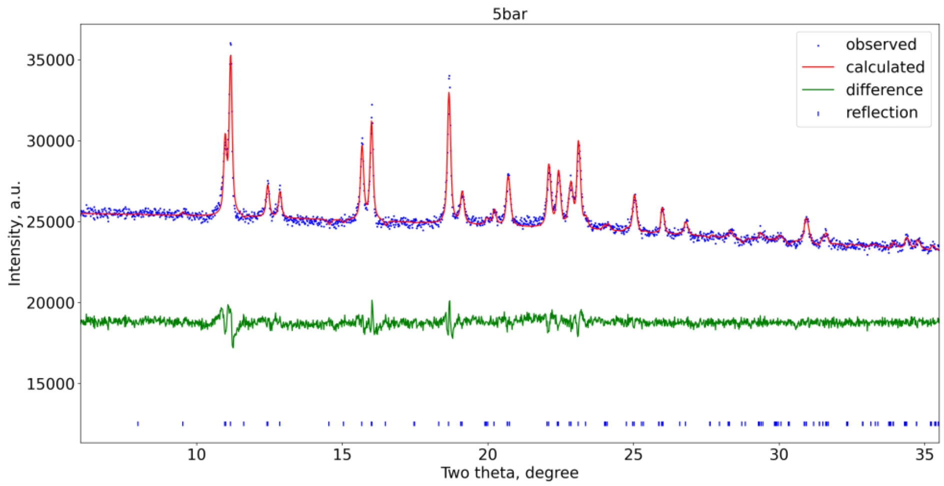

Figure S41. Profile fit of *in situ* CO_2-loaded PXRD pattern of **X-dia-5-Co-β** at 5 bar and 298 K.

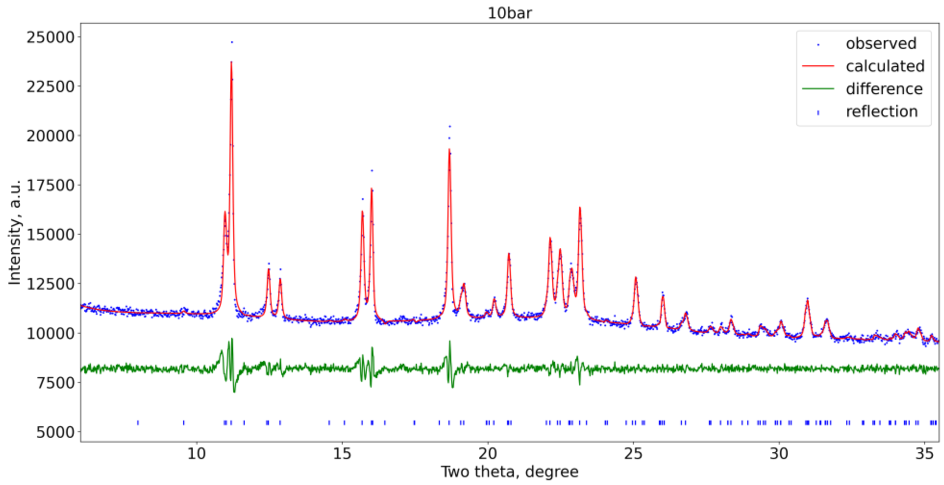

Figure S42. Profile fit of *in situ* CO_2-loaded PXRD pattern of **X-dia-5-Co-β** at 10 bar and 298 K.

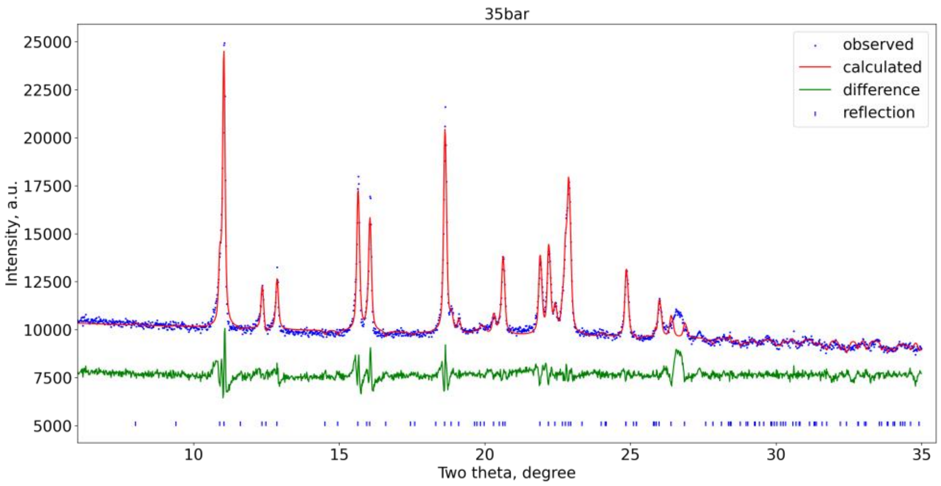

Figure S43. Profile fit of in situ CO_2-loaded PXRD pattern of **X-dia-5-Co-β** at 35 bar and 298 K.

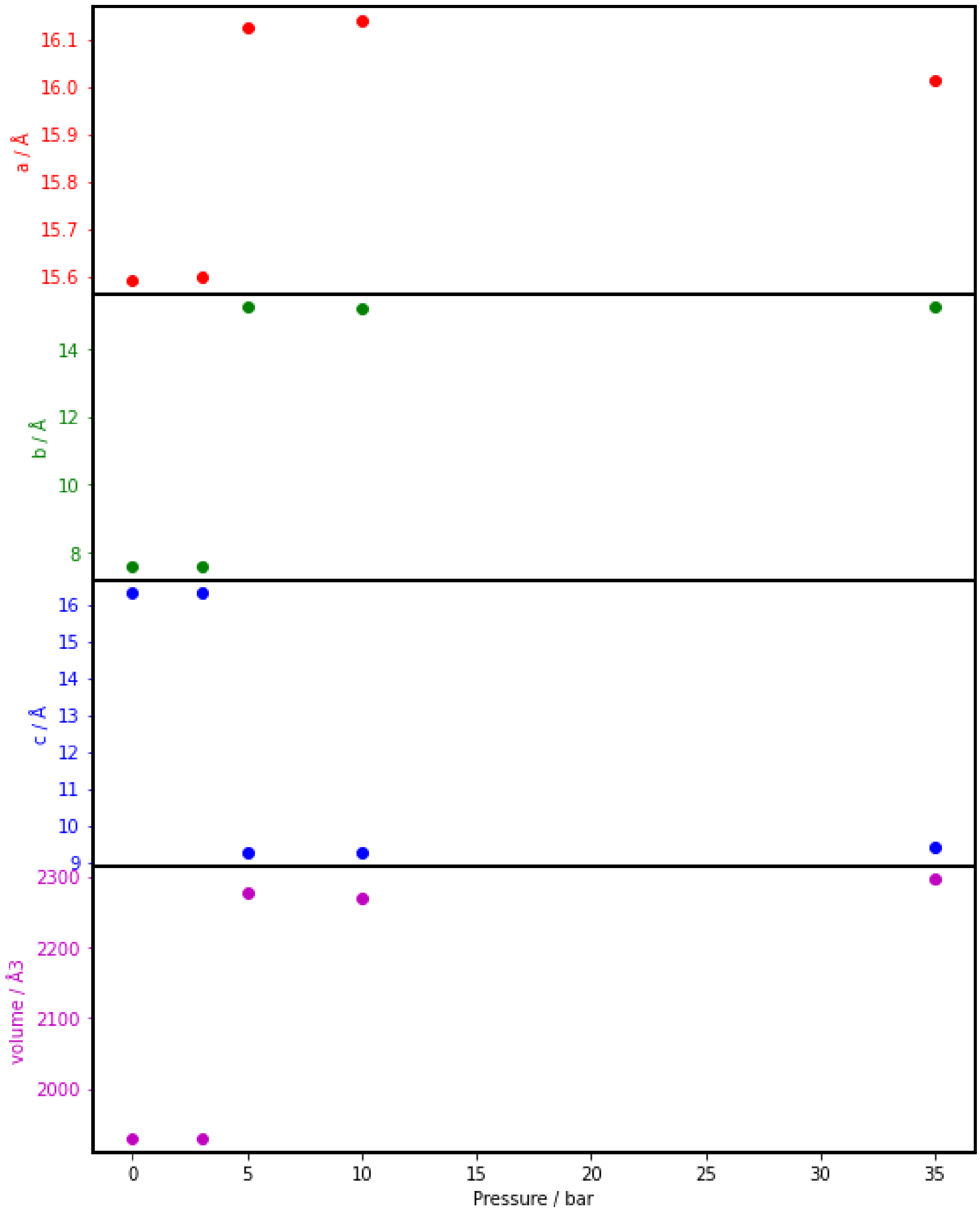

Figure S44. Unit cell parameters of **X-dia-5-Co-β** obtained from Pawley profile fits of high-pressure *in situ* CO_2-loaded PXRD patterns at 298 K.

Table S6. Unit cell parameters of **X-dia-4-Co-β** obtained from Pawley profile fits of *in situ* CO_2-loaded PXRD patterns at 298 K in the low-pressure region (top; 0 to 1 bar) and high-pressure region (bottom; 0 to 10 bar).

Pressure (bar)	Space Group	a (Å)	b (Å)	c (Å)	α = β = γ (°)	V (Å^3)
0	*Pnna*	17.9838	14.2268	7.8510	90	2008.69
0.23	*Pnna*	17.9349	14.1790	7.9405	90	2019.25
0.52	*Pnna*	17.8894	14.1416	8.0298	90	2031.40
0.81	*Pnna*	17.8472	14.1114	8.1038	90	2040.93
1.01	*Pnna*	17.8349	14.1018	8.1392	90	2047.04
0	P*222*	17.9674	14.1309	7.8492	90	1992.89
3	P*222*	17.8522	13.9375	8.2587	90	2054.88
5	P*222*	17.8690	13.9412	8.2454	90	2054.05
10	P*222*	17.8798	13.9496	8.2348	90	2053.89

Table S7. Unit cell parameters of **X-dia-5-Co-β** obtained from Pawley profile fits of *in situ* CO_2-loaded PXRD patterns at 298 K in the high pressure region (0 to 35 bar).

Pressure (bar)	Space Group	a (Å)	b (Å)	c (Å)	α = β = γ (°)	V (Å^3)
0	$Pna2_1$	15.5903	7.5870	16.3093	90	1929.13
3	$Pna2_1$	15.5971	7.5844	16.3043	90	1928.70
5	P*222*	16.1253	15.2220	9.2786	90	2277.50
10	P*222*	16.1397	15.2032	9.2514	90	2270.05
35	P*222*	16.0134	15.2347	9.4117	90	2296.07

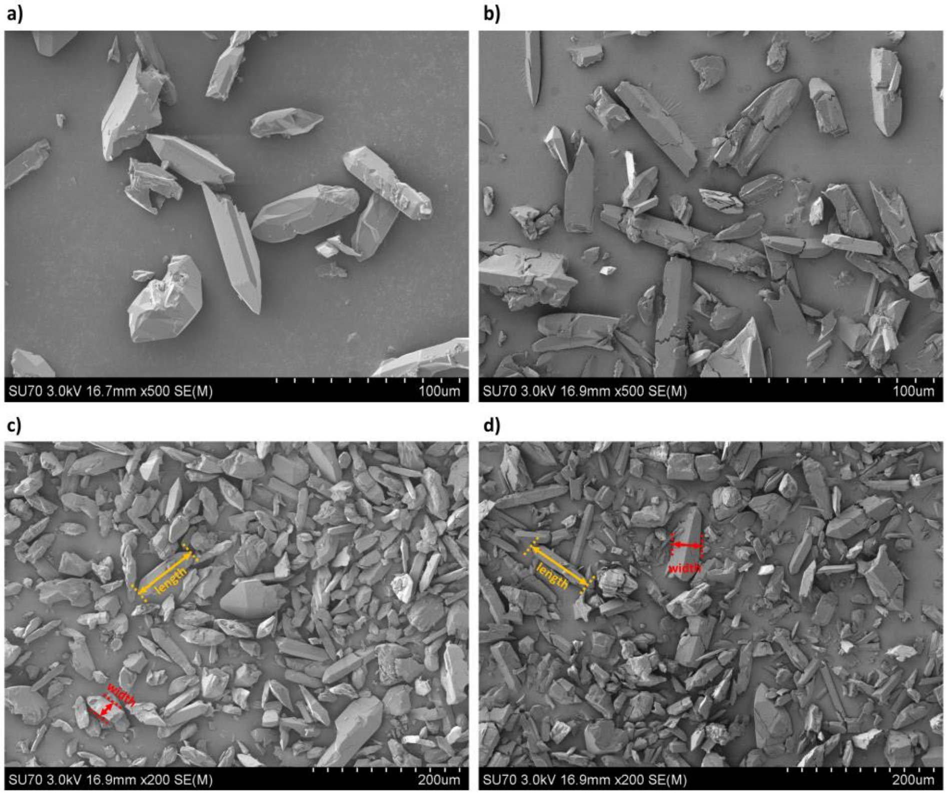

Figure S45. SEM images. Crystal morphology of **X-dia-4-Co-β** (a) and **X-dia-5-Co-β** (b). Representative images for the bulk phase of **X-dia-4-Co-β** (c) and **X-dia-5-Co-β** (d). Examples of crystal length (orange) and crystal width (red) are marked.

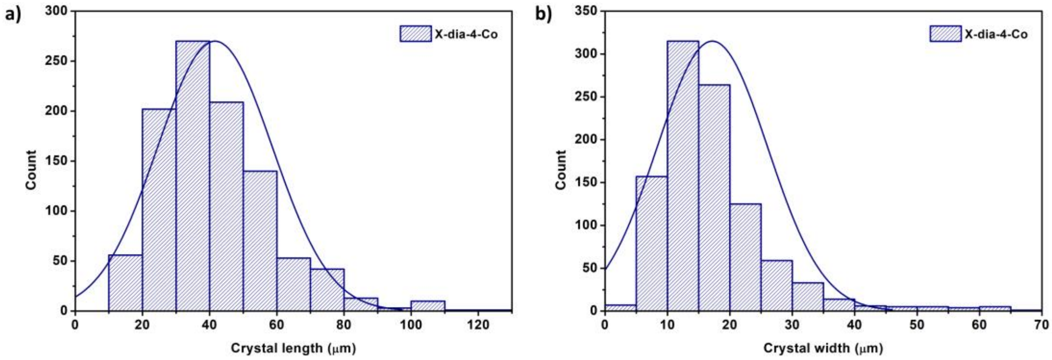

Figure S46. Crystal size distributions for **X-dia-4-Co-β**: (a) crystal length and (b) crystal width.

As shown in Figure S46, the average crystal size of **X-dia-4-Co-β** was found to be 41.6 x 17.2 μm.

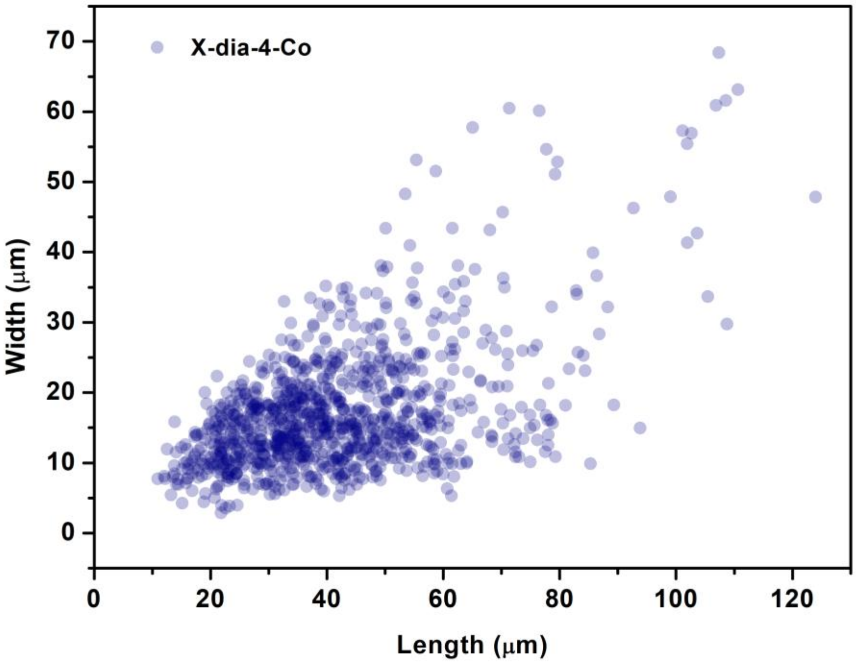

Figure S47. Crystal width plotted against crystal length for **X-dia-4-Co-β**.

Figure S47 shows the elongated shape of the crystals, with most particles having a larger crystal length than crystal width.

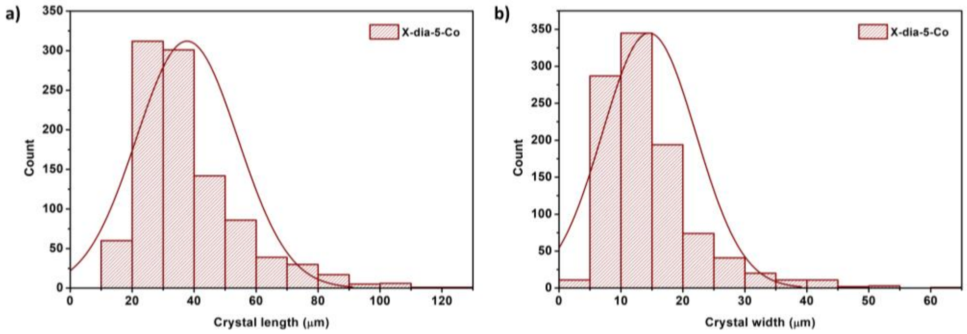

Figure S48. Crystal size distributions for **X-dia-5-Co-β**: (a) crystal length and (b) crystal width.

As shown in Figure S48, the average crystal size of **X-dia-5-Co-β** was found to be 37.7 x 14.5 μm.

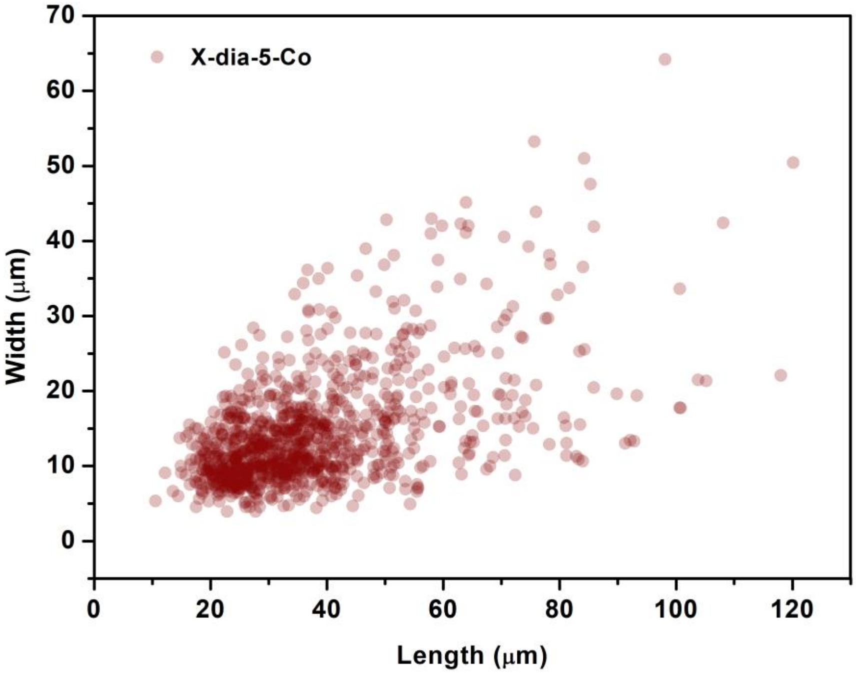

Figure S49. Crystal width plotted against crystal length for **X-dia-5-Co-β**.

Figure S49 shows the elongated shape of the crystals, with most particles having a larger crystal length than crystal width.

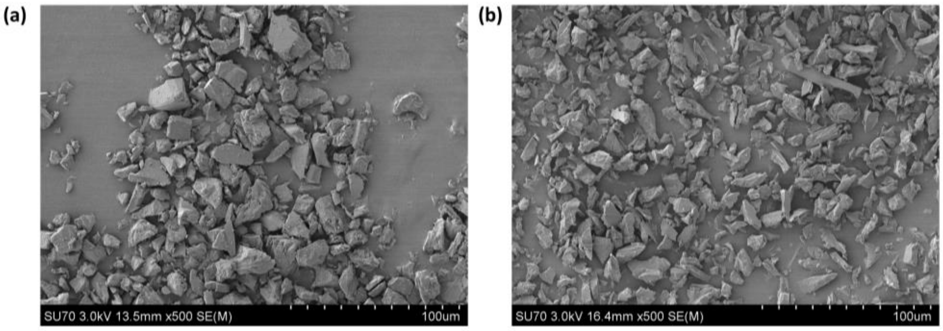

Figure S50. SEM images post CO_2 sorption for: (a) **X-dia-4-Co** and (b) **X-dia-5-Co**.

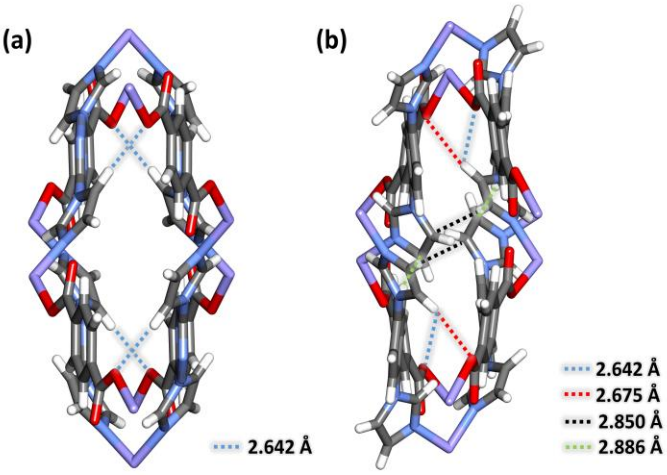

Figure S51. Comparison of hydrogen bonding in the two closed phases: (a) **X-dia-4-Co-β** and (b) **X-dia-5-Co-β**.

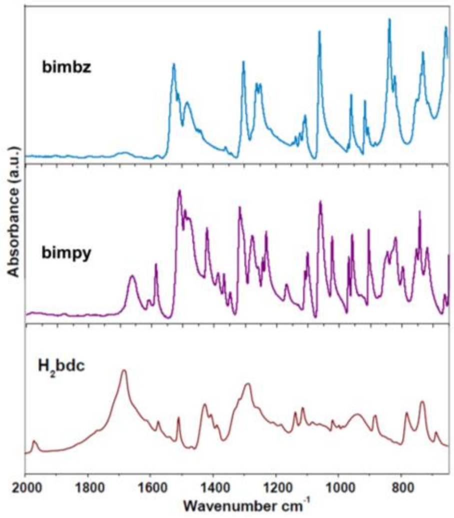

Figure S52. IR spectra of **bimbz**, **bimpy** and **H_2bdc** ligands. All the spectra are referenced to blank KBr pellet.

By inspecting the spectra of the free linkers (Figure S52), bands associated with the **bdc^{2-}** linkers were identified, including the asymmetric ($\nu_{as}(COO^-)$) and symmetric ($\nu_s(COO^-)$) stretching and deformation ($\beta_{as}(COO^-)$) modes of the carboxylate groups, and as well as the phenyl ring modes, including ν(C=C), δ(CH) and γ(CH) bands. The vibrational modes associated with **bdc^{2-}** linker upon coordination with metal ions have been extensively investigated and assigned in detail in former studies of various MOFs that were made of **H_2bdc** linker,[25-32] on the basis of which, the intense band observed at 1570-1600 cm^{-1} in **X-dia-5-Co** and **X-dia-4-Co** yet absent in the spectra of the free linkers was attributed to asymmetric stretching mode ν_{as} of the COO^- group (see Figure S52). The stretching band $\nu_s(COO^-)$ is usually separated from $\nu_{as}(COO^-)$ by less than 200 cm^{-1} when carboxylate coordinates with metal ion through bidentate mode,[33] and exhibits a lower intensity compared with $\nu_{as.}$[28, 30] It is thus expected that $\nu_s(COO^-)$ mode of **X-dia-5-Co** and **X-dia-4-Co** appears at lower frequency, around 1420-1370 cm^{-1}. A distinct band that was observed at 770 and 774 cm^{-1} in **X-dia-5-Co** and **X-dia-4-Co**, respectively, but lacking in the spectra of free organic linkers can be assigned to the deformation mode β of COO^- group.[32, 34] In addition, the phenyl ring modes of **bdc^{2-}** linker including ν(C=C), δ(CH) and γ(CH) were also observed and summarized in Table S8.

Table S8. Summary of selected phonon modes of **X-dia-5-Co** and **X-dia-4-Co**. Notations and acronyms: **ν**, stretch; **δ**, in plane deformation; **γ**, out of plane deformation; **β**, bend; **ph**, phenyl; **az**, azole; **s**, symmetric; and **as**, asymmetric.

**Assignment**	**Frequency Position** (cm^{-1})			
	**X-dia-5-Co** pristine	**X-dia-5-Co** CO_2 at 10/35 bar	**X-dia-4-Co** pristine	**X-dia-4-Co** CO_2 at 35 bar
**bdc^{2-} linker**				
ν_{as}(COO)	1574	1557	1600-1550	1600-1550
ν_{s}(COO)	1420-1370	1420-1370	1420-1350	1420-1350
β_{as}(COO)	770	<770	774	<774
$\nu(CC)_{ph}$	1360	1360	1366	1366
δ(CH)	1016	1016	1011	1011
γ(CH)	889	889	891	891
**bimbz/bimpy linker**				
$\nu(CC)_{ph,\ \mathbf{bimbz}}$	1340	1328	-	-
$\nu(CC)_{ph,\ \mathbf{bimpy}}$	-	-	1603	<1600
$\delta(CH)_{az}$, next to phenyl ring	1283<br>1242<br>1071	1267<br>1237<br>1067	1272<br>1242<br>1072	1275<br>1245<br>1072
mixed $\delta(CH)_{az}$	1102	1102	1113	1108
ν(CN), near to Co-N	1127	1132	1140	1142
$\gamma(CH)_{ph}$	828	834	826	828

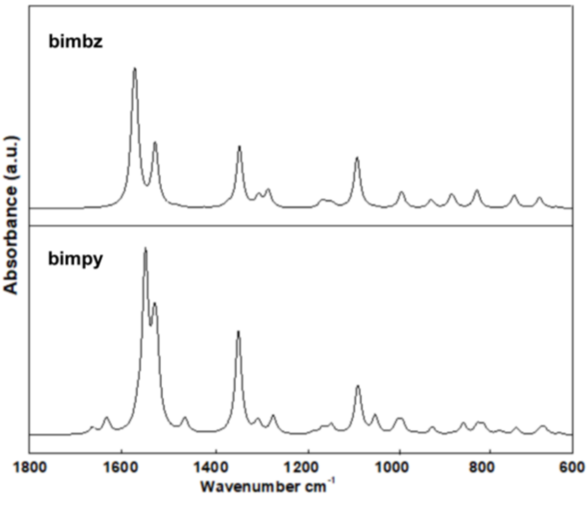

Figure S53. Calculated spectra of **bimbz** and **bimpy** ligands.

Figure S53 shows the calculated vibrational modes of the **bimbz** and **bimpy** linkers using DFT method, which was performed in order to assign the bands pertinent to their vibrations. The spectra of **bimbz** and **bimpy** are quite similar in most of regions, except that **bimpy** displays a series of new bands that are not present in **bimbz**, such as the features around 1600 and 1400 cm^{-1}. These new features arise from the insertion of N atom into the phenyl ring, which not only

produces new vibrations but also breaks the molecular symmetry, thus making some modes IR-active.[35]

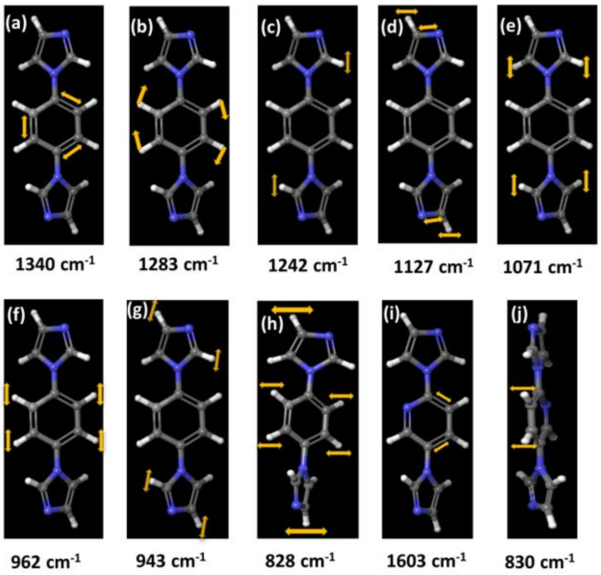

Figure S54. Calculated vibrational modes of: (a-h) **bimbz** and (i, j) **bimpy** linkers that show significant changes upon loading of CO_2. The frequency positions are determined from IR spectra of activated **X-dia-5-Co** and **X-dia-4-Co**, respectively (see Figure 5 in the main document). The yellow arrows represent the eigenvectors of the vibrational modes. Color scheme: Grey = C, blue = N, and white = H.

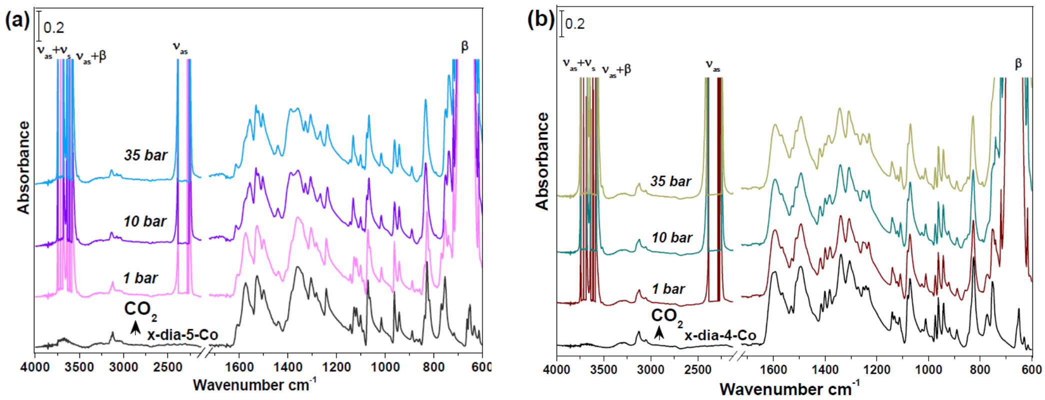

Figure S55. IR spectra of: (a) **X-dia-5-Co** and (b) **X-dia-4-Co** upon loading CO_2 as a function of pressure. The signal of gas phase CO_2 spectra including stretching (ν_{as}), bending (β), and combination ($\nu_{as} + \nu_s$, $\nu_{as}+\beta$) bands is out of scale. All the spectra are referenced to blank KBr pellet.

Table S9. Relative energies (kJ/mol per mol unit cell) of four of the different possible structures of **X-dia-4-Co** obtained with DFT calculations at the experimental cell parameters (**Table S1**). For the N atom positions see Figures S3-S5.

**X-dia-4-Co**	**X-dia-4-Co-1st**	**X-dia-4-Co-2nd**	**X-dia-4-Co-3rd**	**X-dia-4-Co-4th**
Relative energy **X-dia-4-Co-β**	0.0	0.0	30.3	30.3
Relative energy **X-dia-4-Co-α**	154.1	155.3	157.4	156.8

Table S10. Adsorption enthalpy and Gibbs free energy for CO_2 in **X-dia-4-Co** compared to images with relevant volumes for **X-dia-5-Co.** Cell parameters were kept fixed during optimization of all structures while, atomic positions of empty host and empty host + CO_2 completely relaxed. For all **X-dia-4-Co** structures, the cell volume is 2036.27 Å^3 and cell parameters are: a=14.120 Å, b=17.765 Å, c=8.118 Å, α=β=γ=90º. compare to the optimized the second, third and fourth NEB image for **X-dia-5-Co** ($V_{2,\mathbf{Xdia\text{-}5}}$ = 2072.40 Å^3, a=14.923 Å, b=8.367 Å, c=16.598 Å, α=β=γ=90º; $V_{3,\mathbf{Xdia\text{-}5}}$ = 2149.89 Å^3, a=14.820 Å, b=8.842 Å, c=16.406 Å, α=β=γ=90º; $V_{4,\mathbf{Xdia\text{-}5}}$ = 2223.40 Å^3, a=14.717 Å, b=8.318 Å, c=16.213 Å, α=β=γ=90º; $V_{5,\mathbf{Xdia\text{-}5}}$ = 2292.98 Å3, a=14.6131 Å, b=9.79434 Å, c=16.0208 Å, α=β=γ=90º).

**Structures**	$\Delta \boldsymbol{H_{ads}}$ **(kJ/mol)**	$\Delta \boldsymbol{G_{ads}}$ **(kJ/mol)**
**X-dia-4-Co-1st** (V_1= *2036.27 Å^3*)	-37.2	0.7
**X-dia-4-Co-2nd** (V_1= *2036.27 Å^3*)	-40.7	-2.0
**X-dia-4-Co-3rd** (V_1= *2036.27 Å^3*)	-37.2	3.1
**X-dia-4-Co-4th** (V_1= *2036.27 Å^3*)	-43.7	-6.1
**X-dia-5-Co** (V_2= *2072.40 Å^3*)	-2.2	36.8
**X-dia-5-Co** (V_3= *2149.89 Å^3*)	-16.0	23.6
**X-dia-5-Co** (V_4= *2223.40 Å^3*)	-29.5	8.5
**X-dia-5-Co** (V_5= *2292.98 Å^3*)	-37.3	-0.31

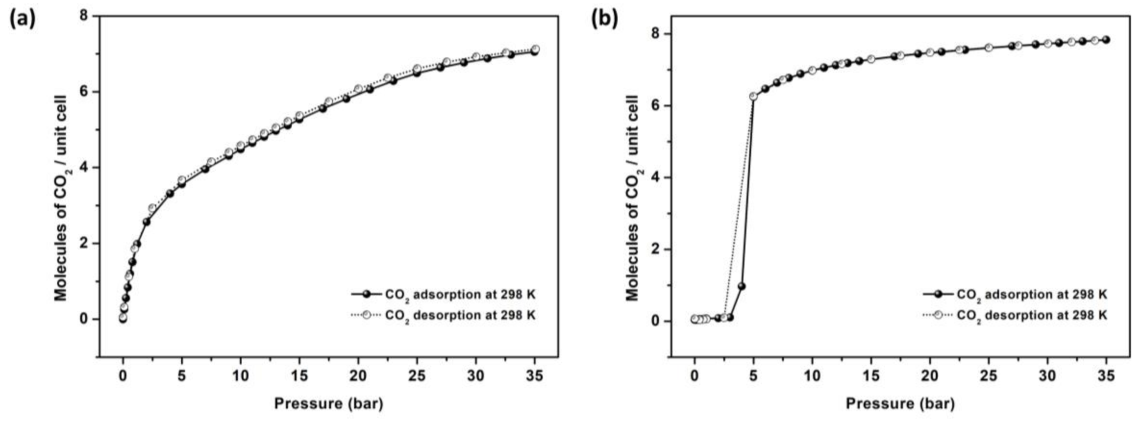

Figure S56. High-pressure CO_2 isotherms collected at 298 K for **X-dia-4-Co** (a) and **X-dia-5-Co** (b), expressed in molecules of CO_2/unit cell as a function of pressure.

Table S11. Lennard-Jones (LJ) parameters representing framework atoms. The interaction with the sorbate molecules was calculated using Lorentz-Berthelot mixing rules.

Atom	ε/k_B (K)	σ (Å)
Co	7.04507	2.55866
N	38.94920	3.26256
C	47.85620	3.47299
O	48.15810	3.03315
H	7.64893	2.84642

Table S12. Point charges for CO_2.

Molecule	Atom	Point charge (e)
CO_2	C	0.7
	O	-0.35

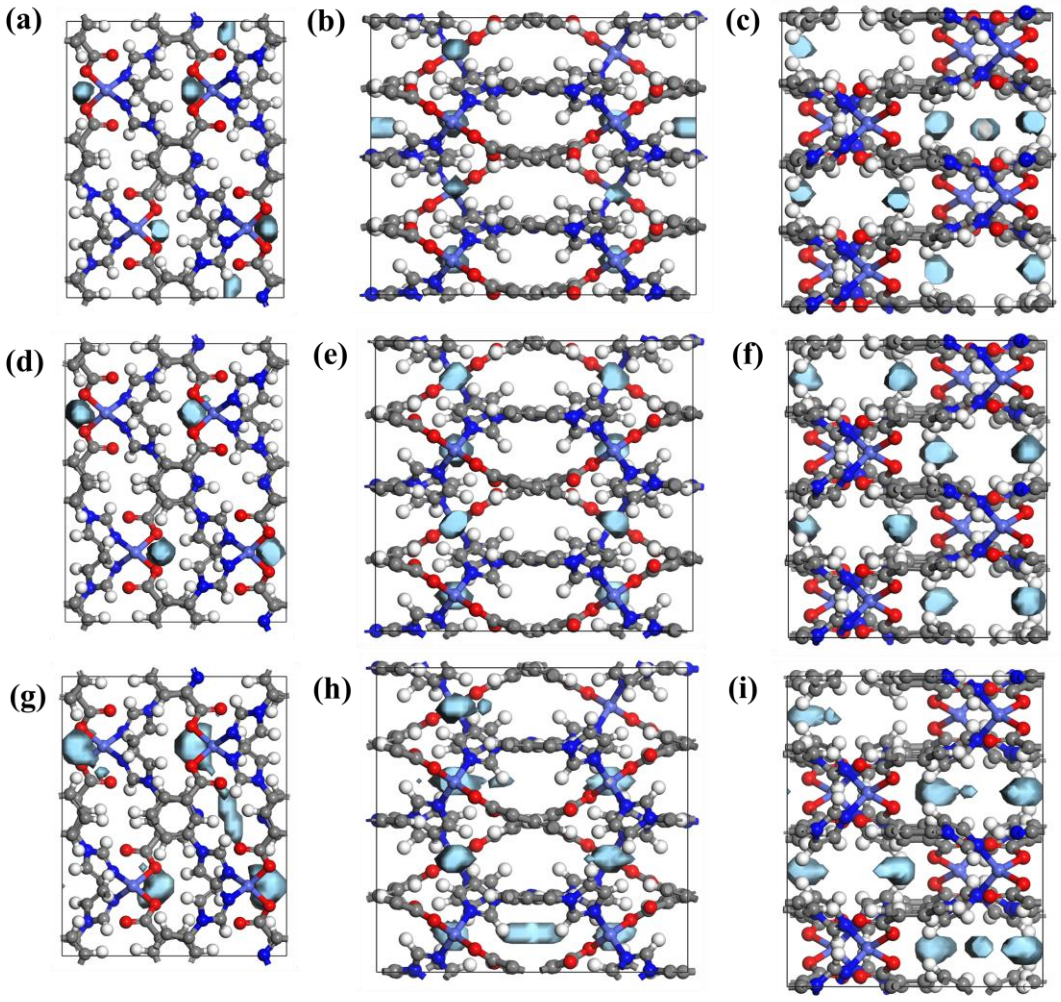

Figure S57. Visualization from different view angles of CO_2 binding site isosurfaces from CMC simulations with 8 adsorbates in **X-dia-4-Co-1st**, (a), (b), and (c) **X-dia-4-Co-1st** (V_0 = 2×1962.02 Å^3); (d), (e), and (f) **X-dia-4-Co-1st** (V_1 = 2×2036.28 Å^3); (g), (h), and (i) **X-dia-4-Co-1st** (V_2 = 2×2106.73 Å^3). Color codes: N, blue; Co, purple; H, white; C, grey; O, red.

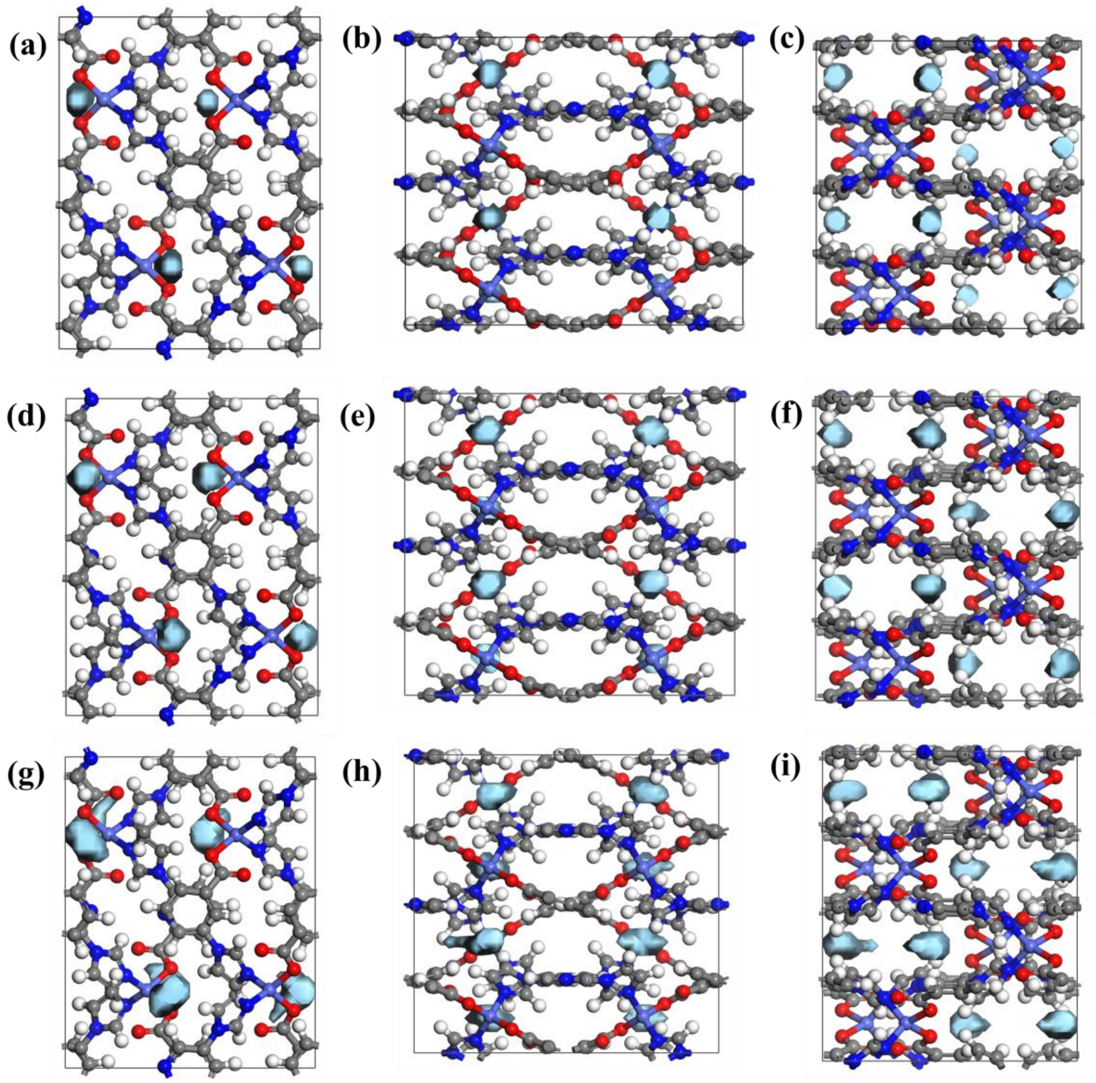

Figure S58. Visualization from different view angles of CO_2 binding site isosurfaces from CMC simulations with 8 adsorbates in **X-dia-4-Co-2nd**, (a), (b), and (c) **X-dia-4-Co-2nd** (V_0 = 2×1962.02 Å^3); (d), (e), and (f) **X-dia-4-Co-2nd** (V_1 = 2×2036.28 Å^3); (g), (h), and (i) **X-dia-4-Co-2nd** (V_2 = 2×2106.73 Å^3). Color codes: N, blue; Co, purple; H, white; C, grey; O, red.

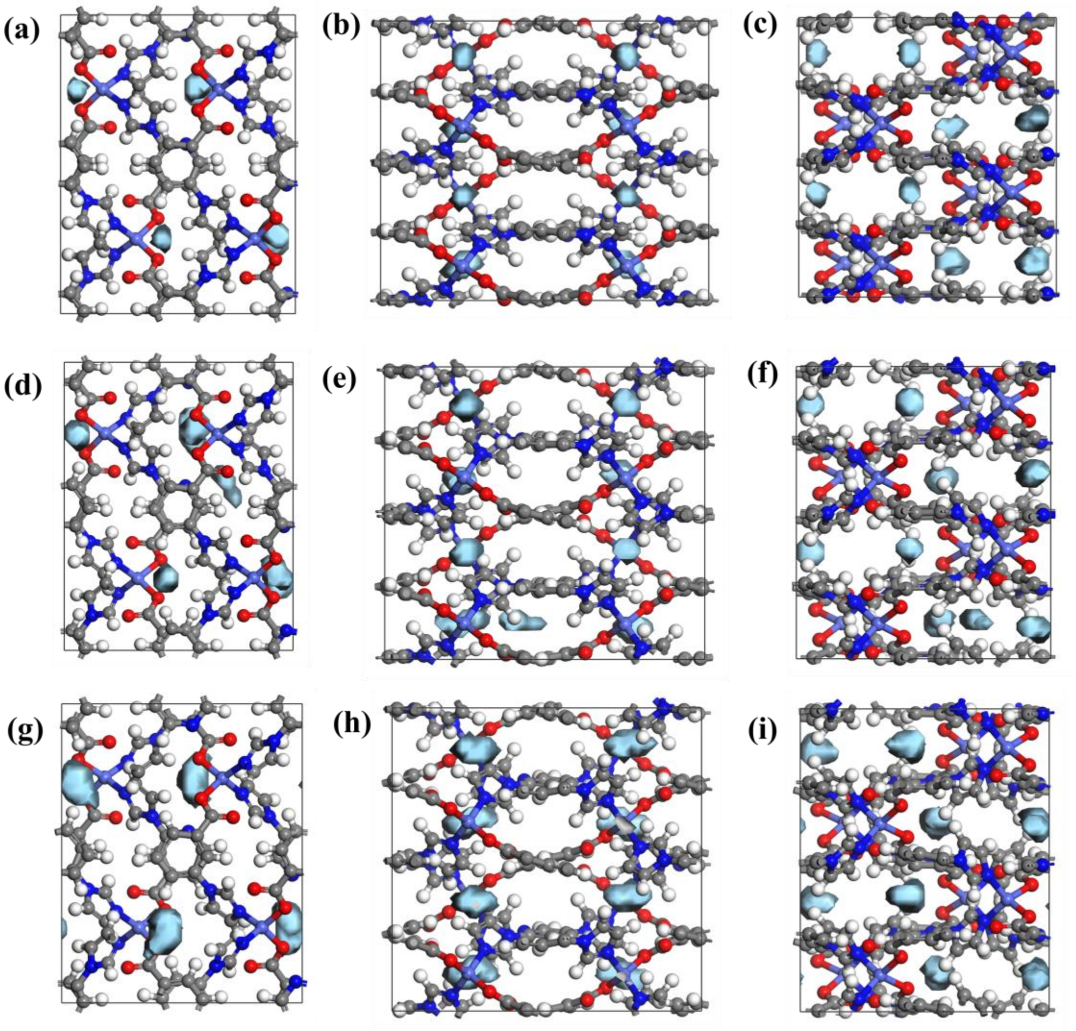

Figure S59. Visualization from different view angles of CO_2 binding site isosurfaces from CMC simulations with 8 adsorbates in **X-dia-4-Co-3rd**, (a), (b), and (c) **X-dia-4-Co-3rd** (V_0 = 2×1962.02 Å^3); (d), (e), and (f) **X-dia-4-Co-3rd** (V_1 = 2×2036.28 Å^3); (g), (h), and (i) **X-dia-4-Co-3rd** (V_2 = 2×2106.73 Å^3). Color codes: N, blue; Co, purple; H, white; C, grey; O, red.

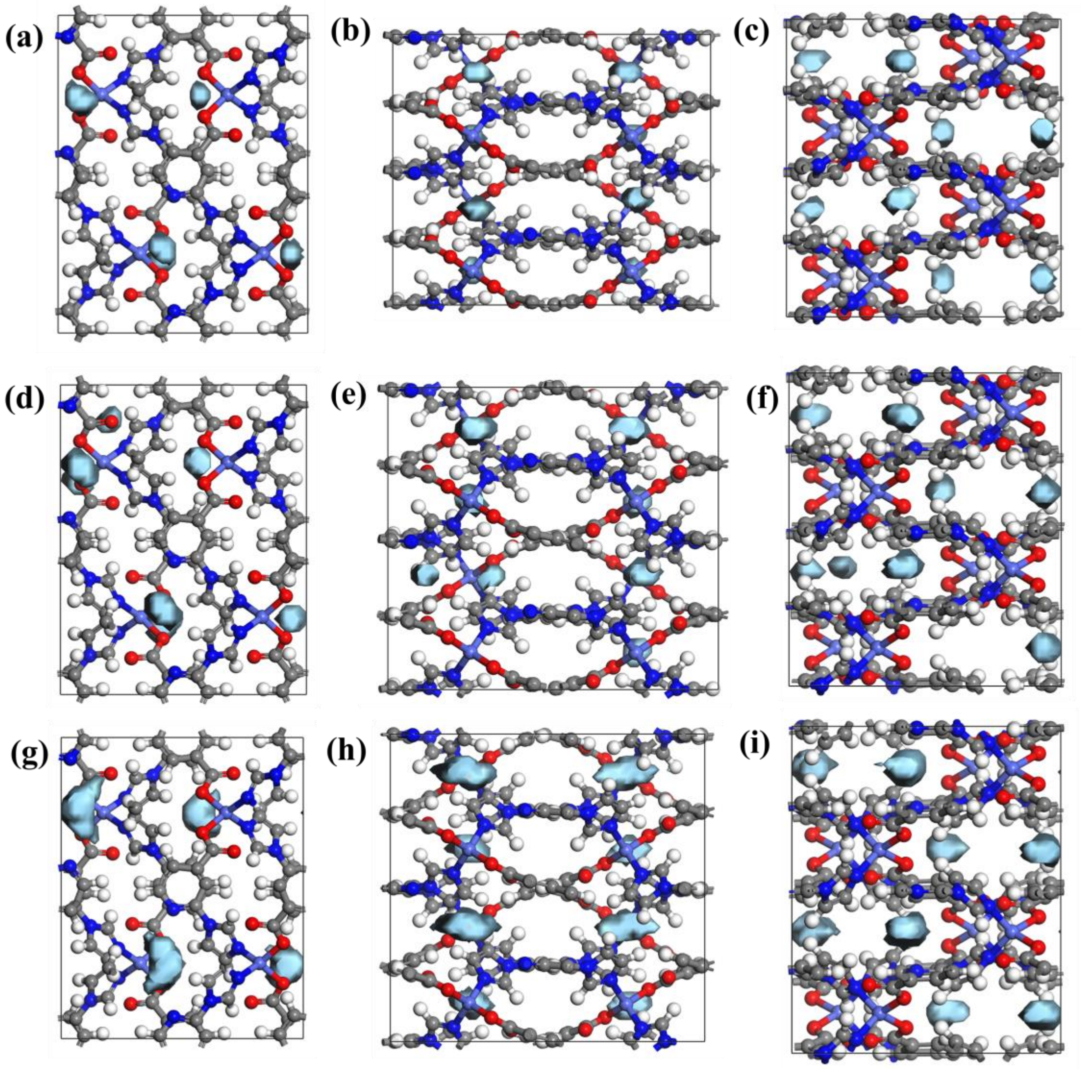

Figure S60. Visualization from different view angles of CO_2 binding site isosurfaces from CMC simulations with 8 adsorbates in **X-dia-4-Co-4th**, (a), (b), and (c) **X-dia-4-Co-4th** (V_0 = 2×1962.02 Å^3); (d), (e), and (f) **X-dia-4-Co-4th** (V_1 = 2×2036.28 Å^3); (g), (h), and (i) **X-dia-4-Co-4th** (V_2 = 2×2106.73 Å^3). Color codes: N, blue; Co, purple; H, white; C, grey; O, red.

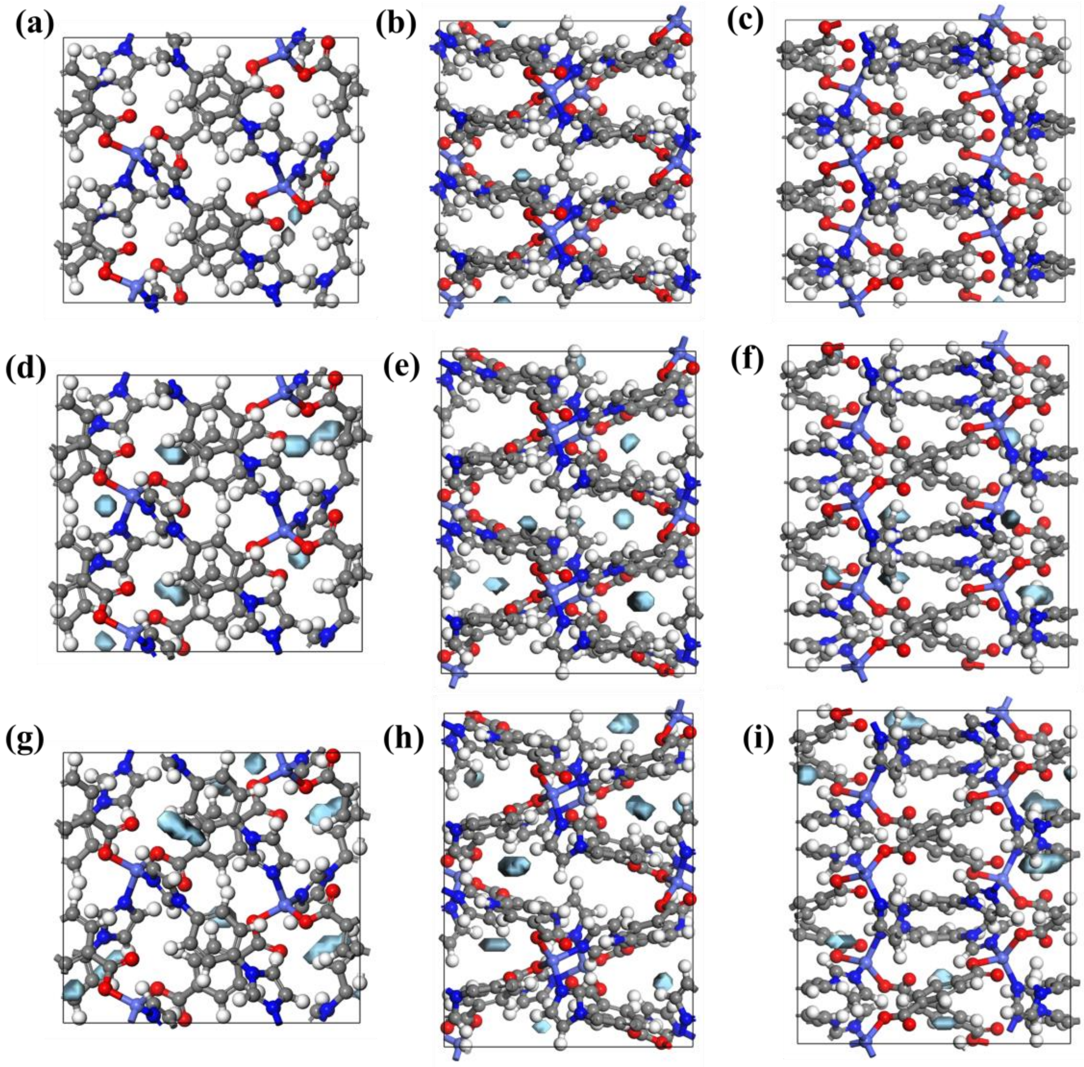

Figure S61. Visualization from different view angles of CO_2 binding site isosurfaces from CMC simulations in **X-dia-5-Co**, (a), (b), and (c) **X-dia-5-Co** (V_2 = 2×2072.40 Å^3), there are 2 CO_2 adsorption positions per unit cell; (d), (e), and (f) **X-dia-5-Co** (V_3= 2×2149.89 Å^3) there are 3 CO_2 adsorption positions per unit cell; (g), (h), and (i) **X-dia-5-Co** (V_4= 2×2223.40 Å^3**)** there are 4 CO_2 adsorption positions per unit cell. Color codes: N, blue; Co, purple; H, white; C, grey; O, red.

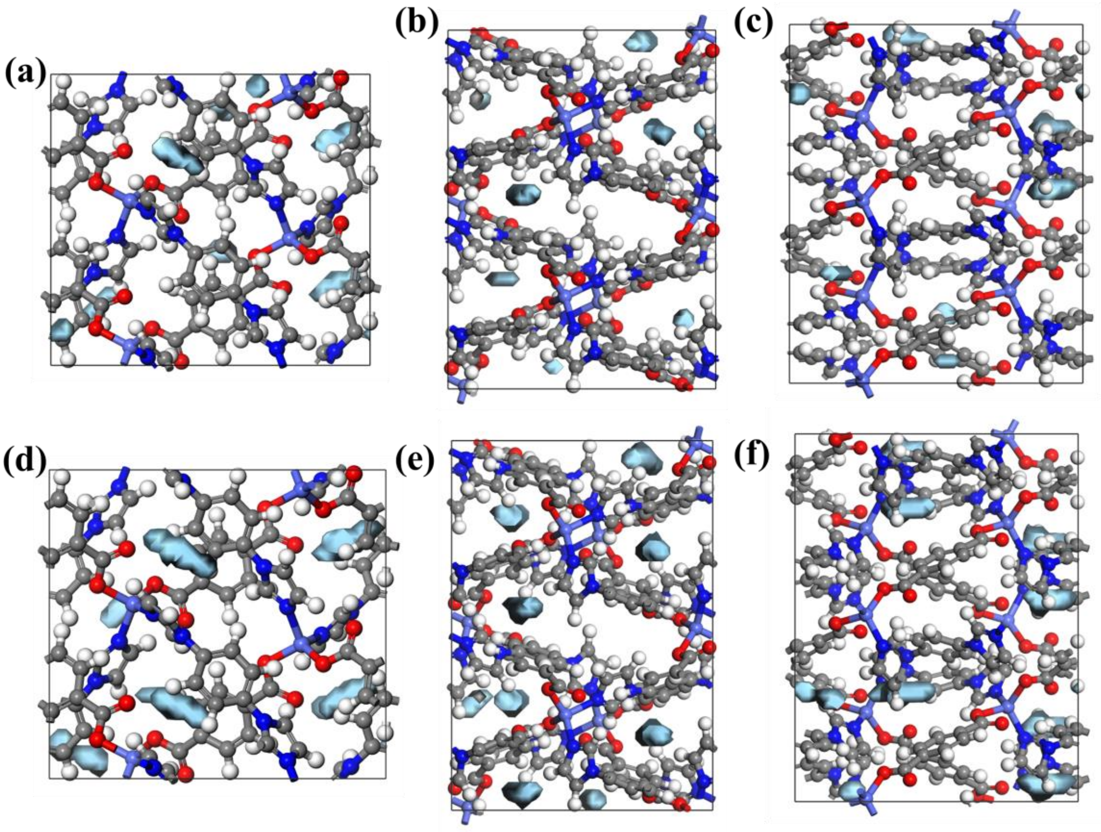

Figure S62. Visualization from different view angles of CO_2 binding site isosurfaces from CMC simulations with 8 adsorbate molecules in **X-dia-5-Co**, (a), (b), and (c) **X-dia-5-Co** (V_5 = 2×2292.98 Å^3); (d), (e), and (f) **X-dia-5-Co** (V_6 = 2×2351.19 Å^3. Color codes: N, blue; Co, purple; H, white; C, grey; O, red.

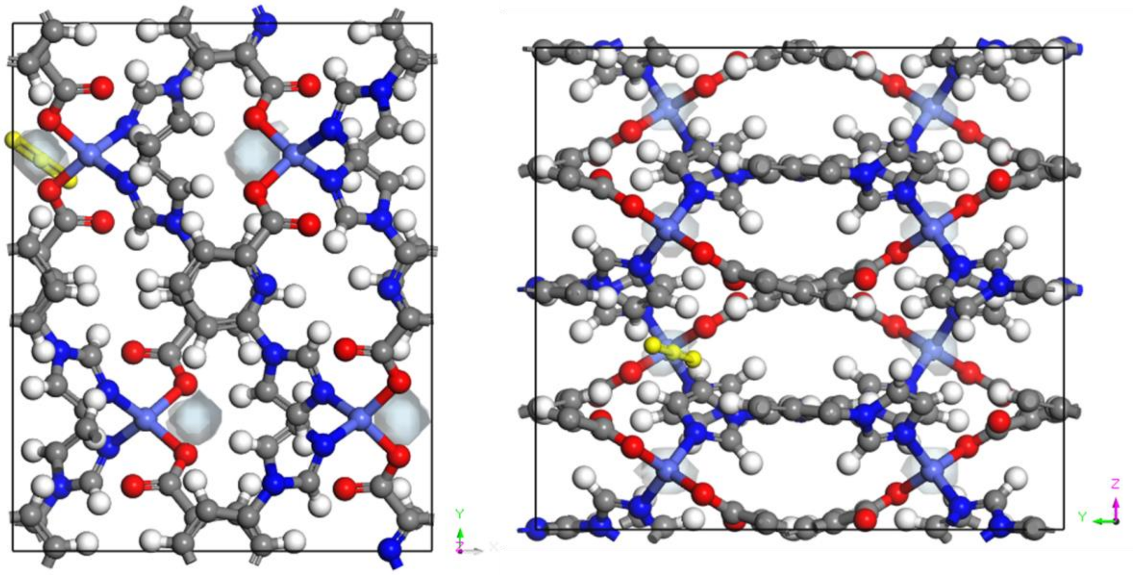

Figure S63. Visualization of CO_2 binding site isosurfaces from CMC simulations together with CO_2 coordinates from the DFT-optimized structures for **X-dia-4-Co-1st** (V_1= 2×2036.27 Å^3**)** framework. Color codes: N, blue; Co, purple; H, white; C, grey; O, red. CO_2 molecules are presented in yellow for clarification.

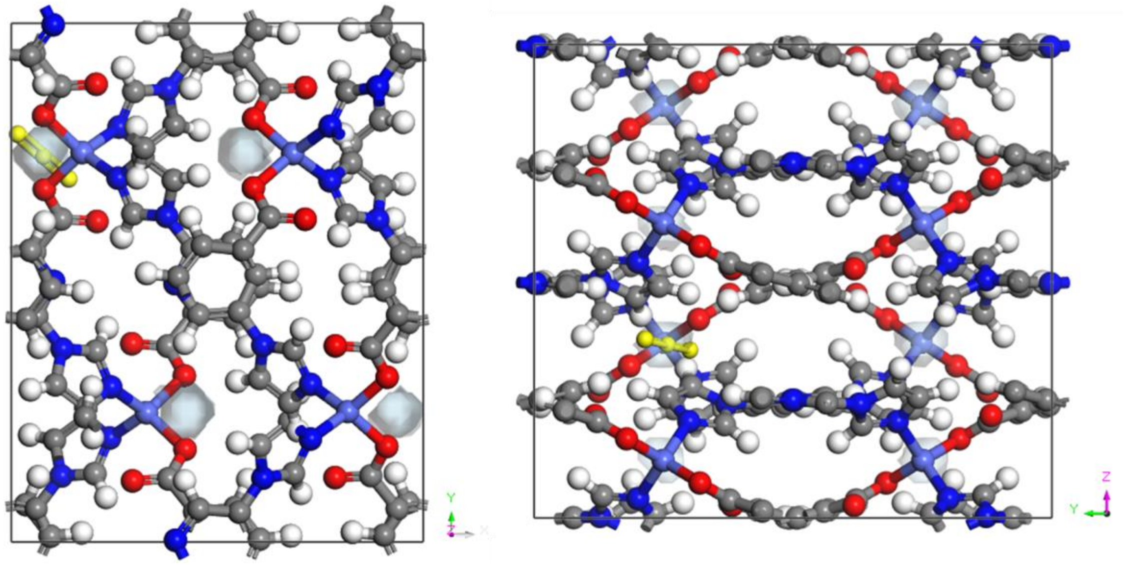

Figure S64. Visualization of CO_2 binding site isosurfaces (blue) from CMC simulations together with CO_2 coordinates from the DFT-optimizations (yellow, ball and stick) for **X-dia-4-Co-2nd (**V_1= 2×2036.27 Å^3**)** framework. Color codes: N, blue; Co, purple; H, white; C, grey; O, red. CO_2 molecules are presented in yellow for clarification.

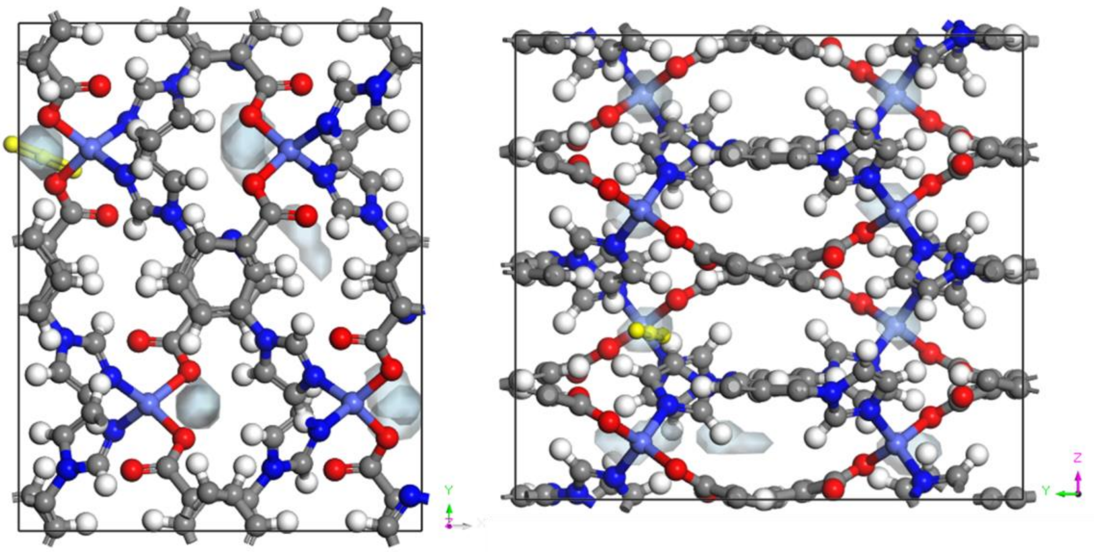

Figure S65. Visualization of CO_2 binding site isosurfaces (blue) from CMC simulations together with CO_2 coordinates from the DFT-optimizations (yellow, ball and stick) for **X-dia-4-Co-3rd** (V$_1$= 2×2036.27 Å^3**)** framework. Color codes: N, blue; Co, purple; H, white; C, grey; O, red. CO_2 molecules are presented in yellow for clarification.

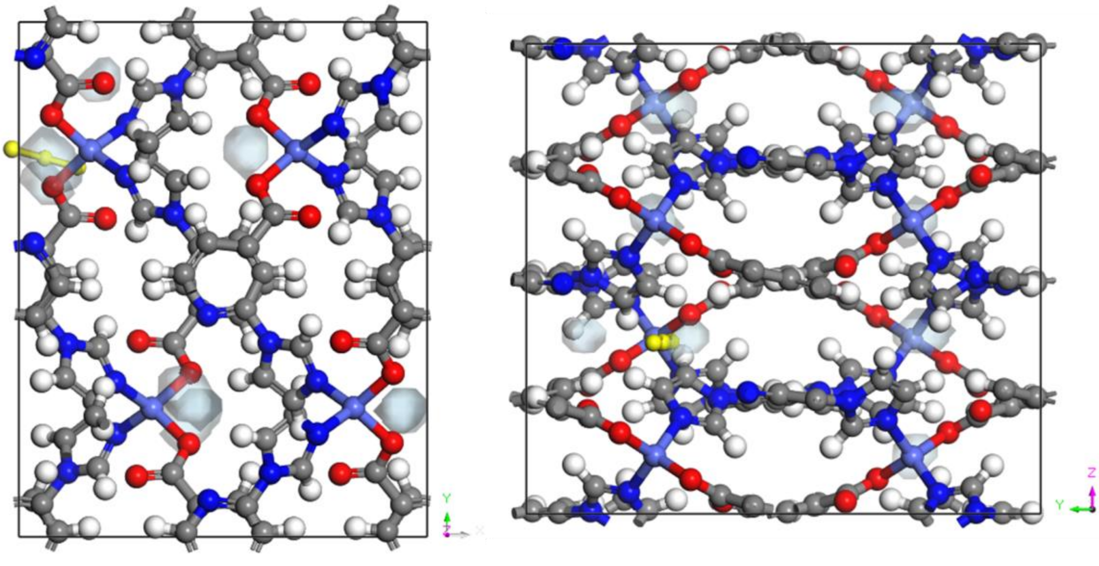

Figure S66. Visualization of CO_2 binding site isosurfaces (blue) from CMC simulations together with CO_2 coordinates from the DFT-optimizations (yellow, ball and stick) for **X-dia-4-Co-4th** (V$_1$= 2×2036.27 Å^3**)** framework. Color codes: N, blue; Co, purple; H, white; C, grey; O, red. CO_2 molecules are presented in yellow for clarification.

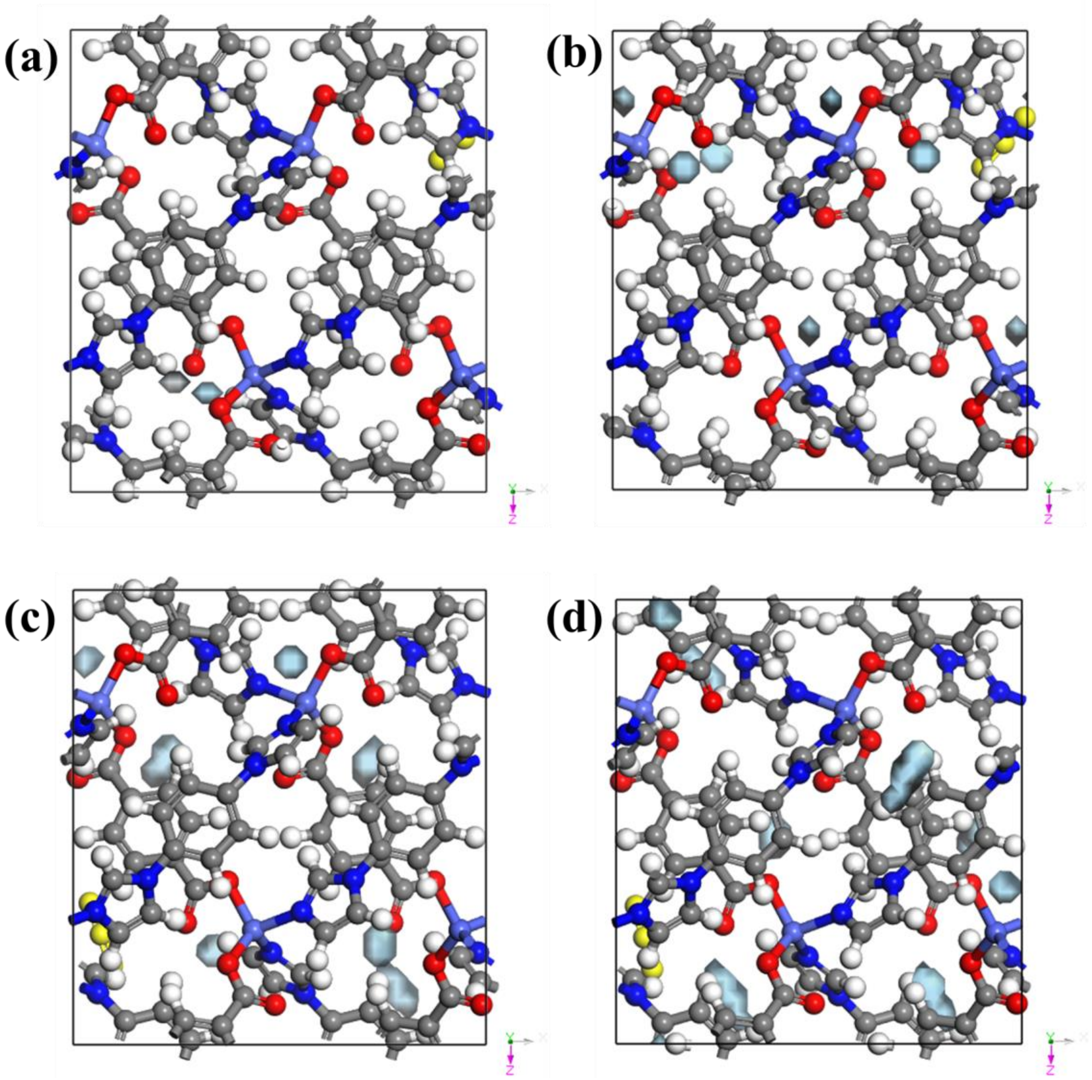

Figure S67. Visualization of CO_2 binding site isosurfaces (blue) from CMC simulations together with CO_2 coordinates from the DFT-optimizations (yellow, ball and stick) for **X-dia-5-Co** (a) V_2 = 2×2072.40 Å^3, (b) V_3 = 2×2149.89 Å^3, (c) V_4 = 2×2223.40 Å^3 and (d) V_5 = 2×2292.98 Å^3 frameworks. Color codes: N, blue; Co, purple; H, white; C, grey; O, red. CO_2 molecules are presented in yellow for clarification.

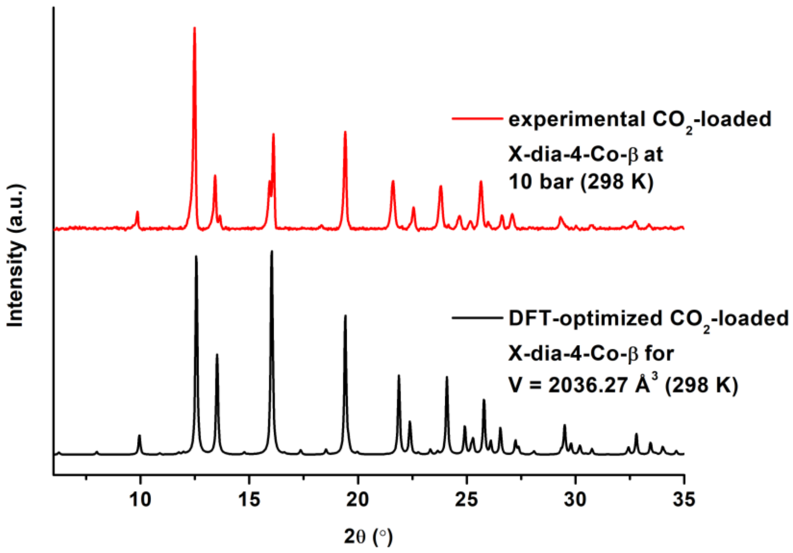

Figure S68. PXRD patterns for the calculated DFT-optimized CO_2-loaded **X-dia-4-Co-β** for V_1= 2036.27 Å^3 (black) and experimental CO_2-loaded **X-dia-4-Co-β** at 10 bar (red).

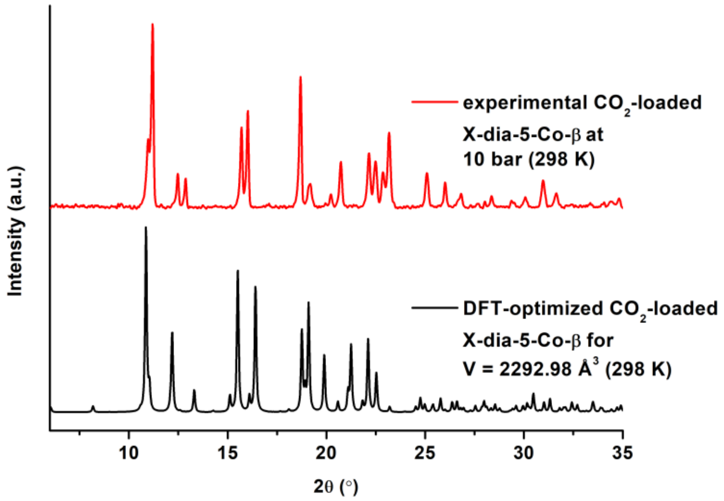

Figure S69. PXRD patterns for the calculated DFT-optimized CO_2-loaded **X-dia-5-Co-β** for V_5 = 2292.98 Å^3 (black) and experimental CO_2-loaded **X-dia-5-Co-β** at 10 bar (red).

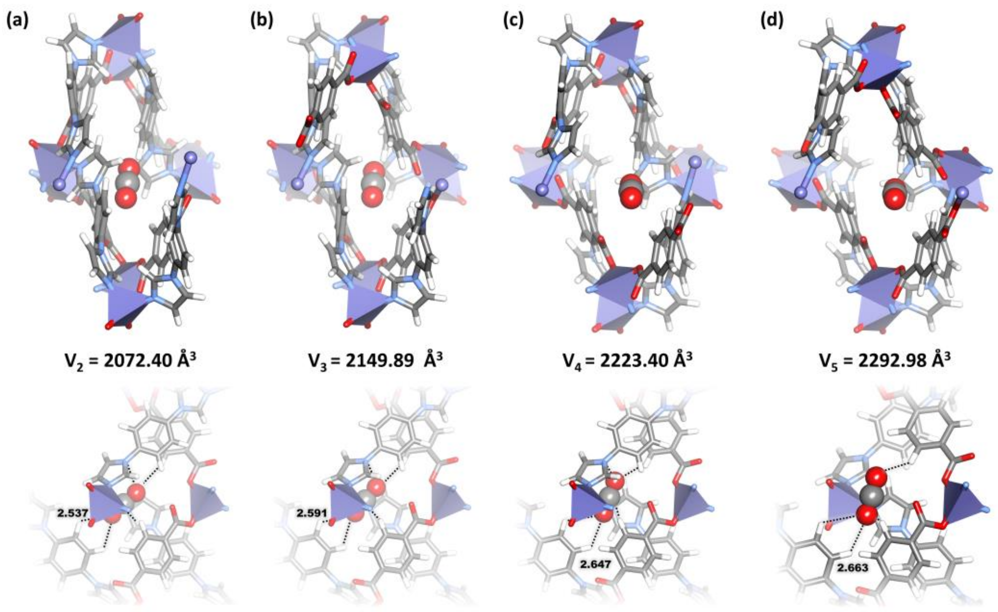

Figure S70. Binding sites of CO_2 in **X-dia-5-Co** for V_2 = 2072.40 Å^3 (a), V_3 = 2149.89 Å^3 (b), V_4 = 2223.40 Å^3 (c) and V_5 = 2292.98 Å^3 (d) obtained with DFT calculations. Selected close contact distances are shown in black dashed lines, while the shortest close contact distance in each framework is listed (in Å).

Table S13. Short contact distances between the framework and the adsorbed CO_2 molecule in the open phases of **X-dia-4-Co-1st, X-dia-4-Co-2nd, X-dia-4-Co-3rd and X-dia-4-Co-4th** at V_1 = 2036.27 Å^3 and **X-dia-5-Co** at V_2 = 2072.40, V_3 = 2149.89, V_4 = 2223.40 and V_5 = 2292.98 Å^3, optimized by DFT.

Interaction	Distance (Å)
**X-dia-4-Co-1st (V_1 = 2036.27 Å^3)**	
$O_{CO_2} \cdots H_{bimpy}$	2.657 / 2.785
$O_{CO_2} \cdots H_{bdc^{2-}}$	3.020
$C_{CO_2} \cdots H_{bimpy}$	3.045 / 3.296
$O_{CO_2} \cdots C_{bdc^{2-}}$	3.218
$C_{CO_2} \cdots O_{bdc^{2-}}$	3.349 / 3.402
**X-dia-4-Co-2nd (V_1 = 2036.27 Å^3)**	
$O_{CO_2} \cdots H_{bimpy}$	2.619 / 2.764
$C_{CO_2} \cdots H_{bimpy}$	3.120 / 3.310
$O_{CO_2} \cdots H_{bdc^{2-}}$	3.148
$O_{CO_2} \cdots C_{bdc^{2-}}$	3.309
$C_{CO_2} \cdots O_{bdc^{2-}}$	3.439 / 3.499
**X-dia-4-Co-3rd (V_1 = 2036.27 Å^3)**	
$O_{CO_2} \cdots H_{bimpy}$	2.890 / 2.984
$C_{CO_2} \cdots H_{bimpy}$	2.987 / 3.095
$O_{CO_2} \cdots H_{bdc^{2-}}$	3.100
$O_{CO_2} \cdots C_{bdc^{2-}}$	3.193
$C_{CO_2} \cdots O_{bdc^{2-}}$	3.390 / 3.429
**X-dia-4-Co-4th (V_1 = 2036.27 Å^3)**	
$O_{CO_2} \cdots H_{bdc^{2-}}$	3.195
$O_{CO_2} \cdots C_{bimpy}$	3.277 / 3.302
$O_{CO_2} \cdots C_{bdc^{2-}}$	3.296
$C_{CO_2} \cdots O_{bdc^{2-}}$	3.411 / 3.480
$C_{CO_2} \cdots N_{bimpy}$	3.465
**X-dia-5-Co (V_2 = 2072.40 Å^3)**	
$O_{CO_2} \cdots H_{bimbz}$	2.537 / 2.680 / 3.154
$C_{CO_2} \cdots H_{bdc^{2-}}$	2.659
$O_{CO_2} \cdots H_{bdc^{2-}}$	2.829 / 2.969
$O_{CO_2} \cdots N_{bimbz}$	2.999
$O_{CO_2} \cdots C_{bimbz}$	3.040 / 3.153
$C_{CO_2} \cdots C_{bimbz}$	3.192 / 3.207
**X-dia-5-Co (V_3 = 2149.89 Å^3)**	
$O_{CO_2} \cdots H_{bimbz}$	2.591 / 2.642 / 3.110
$C_{CO_2} \cdots H_{bdc^{2-}}$	2.824
$O_{CO_2} \cdots H_{bdc^{2-}}$	2.827 / 2.910

Table S13 (continued). Short contact distances between the framework and the adsorbed CO_2 molecule in the open phases of **X-dia-4-Co-1st, X-dia-4-Co-2nd, X-dia-4-Co-3rd and X-dia-4-Co-4th** at V_1 = 2036.27 Å^3 and **X-dia-5-Co** at V_2 = 2072.40 Å^3, V_3 = 2149.89 Å^3, V_4 = 2223.40 Å^3 and V_5 = 2292.98 Å^3, optimized by DFT.

$O_{CO_2} \cdots N_{bimbz}$	3.005
$O_{CO_2} \cdots C_{bimbz}$	3.104 / 3.147
$C_{CO_2} \cdots C_{bimbz}$	3.223
**X-dia-5-Co (V_4 = 2223.40 Å^3)**	
$O_{CO_2} \cdots H_{bimbz}$	2.647 / 2.820 / 3.027
$O_{CO_2} \cdots H_{bdc^{2-}}$	2.710 / 2.759
$O_{CO_2} \cdots N_{bimbz}$	3.057
$C_{CO_2} \cdots H_{bdc^{2-}}$	3.059
$O_{CO_2} \cdots C_{bimbz}$	3.184 / 3.203
**X-dia-5-Co (V_5 = 2292.98 Å^3)**	
$O_{CO_2} \cdots H_{bimbz}$	2.663 / 2.885 / 2.999
$O_{CO_2} \cdots H_{bdc^{2-}}$	2.689 / 2.839
$C_{CO_2} \cdots C_{bimbz}$	3.238
$C_{CO_2} \cdots O_{bdc^{2-}}$	3.357

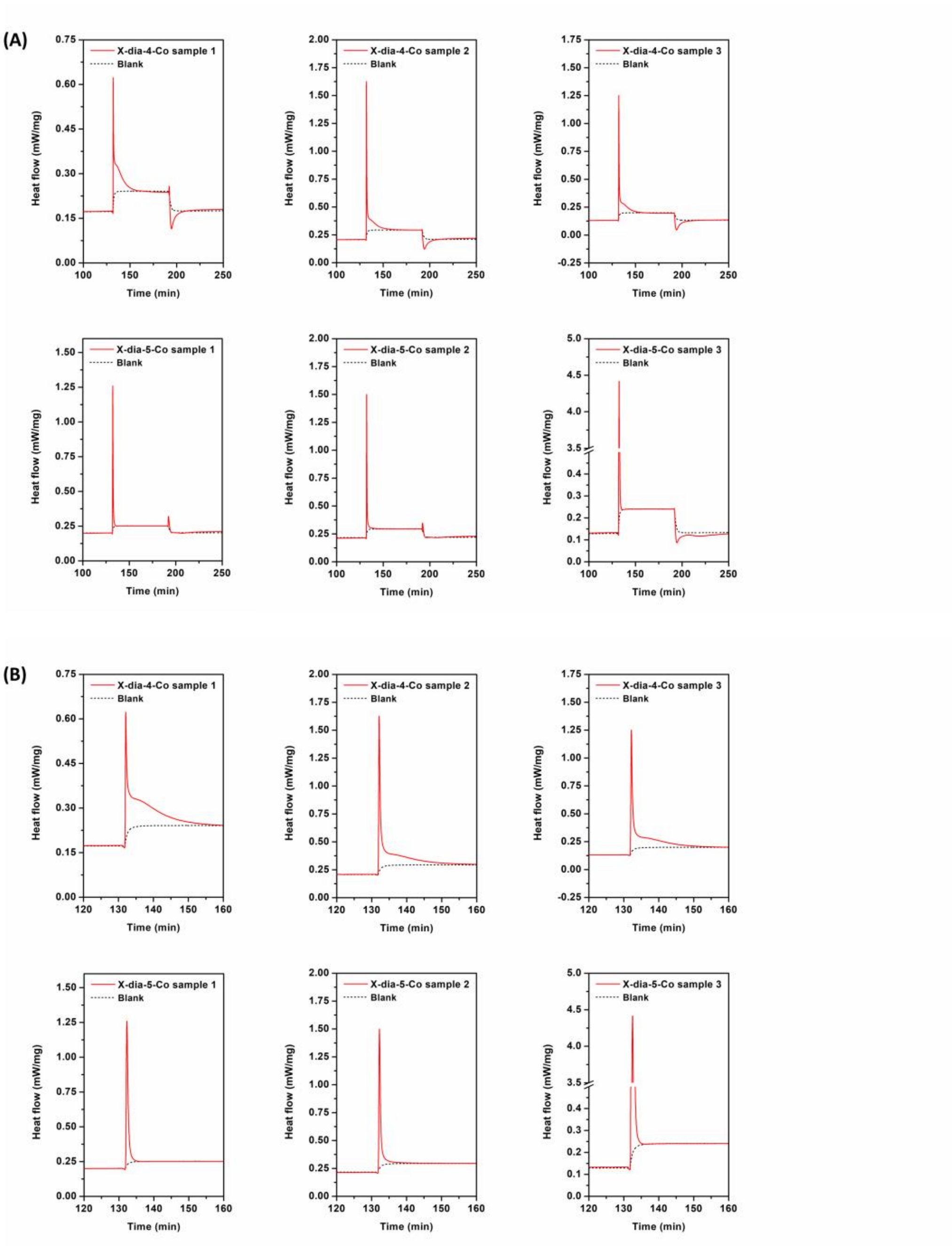

Figure S71. DSC analysis of **X-dia-4-Co-β** and **X-dia-5-Co-β** upon sorption of CO_2 at 198 K. (A) Adsorption and desorption peaks for three different physical samples for each compound. (B) Magnified adsorption peaks.

References

(1) Altman, R. A.; Buchwald, S. L. 4,7-Dimethoxy-1,10-phenanthroline: An Excellent Ligand for the Cu-Catalyzed N-Arylation of Imidazoles. *Organic Letters* **2006**, *8*, 2779-2782.
(2) Chen, H.; Wang, D.; Wang, X.; Huang, W.; Cai, Q.; Ding, K. Mild Conditions for Copper-Catalyzed N-Arylation of Imidazoles. *Synthesis* **2010**, *2010*, 1505-1511.
(3) Francart, T.; van Wieringen, A.; Wouters, J. APEX 3: a multi-purpose test platform for auditory psychophysical experiments. *J Neurosci Methods* **2008**, *172*, 283-293.
(4) Sheldrick, G. sadabs, Version 2008/1, Bruker AXS. *Inc.: Madison, WI* **2008**.
(5) Dolomanov, O. V.; Bourhis, L. J.; Gildea, R. J.; Howard, J. A. K.; Puschmann, H. OLEX2: a complete structure solution, refinement and analysis program. *Journal of Applied Crystallography* **2009**, *42*, 339-341.
(6) Boultif, A.; Louër, D. Indexing of powder diffraction patterns for low-symmetry lattices by the successive dichotomy method. *Journal of Applied Crystallography* **1991**, *24*, 987-993.
(7) David, W. I. F.; Shankland, K.; van de Streek, J.; Pidcock, E.; Motherwell, W. D. S.; Cole, J. C. DASH: a program for crystal structure determination from powder diffraction data. *Journal of Applied Crystallography* **2006**, *39*, 910-915.
(8) Toby, B. H.; Von Dreele, R. B. GSAS-II: the genesis of a modern open-source all purpose crystallography software package. *Journal of Applied Crystallography* **2013**, *46*, 544-549.
(9) Schneider, C. A.; Rasband, W. S.; Eliceiri, K. W. NIH Image to ImageJ: 25 years of image analysis. *Nature Methods* **2012**, *9*, 671-675.
(10) Bochevarov, A. D.; Harder, E.; Hughes, T. F.; Greenwood, J. R.; Braden, D. A.; Philipp, D. M.; Rinaldo, D.; Halls, M. D.; Zhang, J.; Friesner, R. A. Jaguar: A high-performance quantum chemistry software program with strengths in life and materials sciences. *International Journal of Quantum Chemistry* **2013**, *113*, 2110-2142.
(11) Schrödinger Release 2022-2: Jaguar; Schrödinger, LLC, New York, NY, 2021.
(12) Lee, C.; Yang, W.; Parr, R. G. Development of the Colle-Salvetti correlation-energy formula into a functional of the electron density. *Phys Rev B Condens Matter* **1988**, *37*, 785-789.
(13) Becke, A. D. Density-functional thermochemistry. III. The role of exact exchange. *The Journal of Chemical Physics* **1993**, *98*, 5648-5652.
(14) Ditchfield, R.; Hehre, W. J.; Pople, J. A. Self-Consistent Molecular-Orbital Methods. IX. An Extended Gaussian-Type Basis for Molecular-Orbital Studies of Organic Molecules. *The Journal of Chemical Physics* **1971**, *54*, 724-728.
(15) Blöchl, P. E. Projector augmented-wave method. *Physical Review B* **1994**, *50*, 17953-17979.
(16) Kresse, G.; Furthmüller, J. Efficiency of ab-initio total energy calculations for metals and semiconductors using a plane-wave basis set. *Computational Materials Science* **1996**, *6*, 15-50.
(17) Kresse, G.; Furthmüller, J. Efficient iterative schemes for ab initio total-energy calculations using a plane-wave basis set. *Physical Review B* **1996**, *54*, 11169-11186.
(18) Wellendorff, J.; Lundgaard, K. T.; Møgelhøj, A.; Petzold, V.; Landis, D. D.; Nørskov, J. K.; Bligaard, T.; Jacobsen, K. W. Density functionals for surface science: Exchange-correlation model development with Bayesian error estimation. *Physical Review B* **2012**, *85*, 235149.
(19) Monkhorst, H. J.; Pack, J. D. Special points for Brillouin-zone integrations. *Physical Review B* **1976**, *13*, 5188-5192.
(20) Ghysels, A.; Verstraelen, T.; Hemelsoet, K.; Waroquier, M.; Van Speybroeck, V. TAMkin: A Versatile Package for Vibrational Analysis and Chemical Kinetics. *Journal of Chemical Information and Modeling* **2010**, *50*, 1736-1750.
(21) BIOVIA, Dassault Systèmes, Materials Studio, San Diego: Dassault Systèmes, 2022.
(22) Wilmer, C. E.; Kim, K. C.; Snurr, R. Q. An Extended Charge Equilibration Method. *The Journal of Physical Chemistry Letters* **2012**, *3*, 2506-2511.
(23) Dubbeldam, D.; Calero, S.; Ellis, D. E.; Snurr, R. Q. RASPA: molecular simulation software for adsorption and diffusion in flexible nanoporous materials. *Molecular Simulation*

2016, *42*, 81-101.
(24) Dubbeldam, D.; Torres-Knoop, A.; Walton, K. S. On the inner workings of Monte Carlo codes. *Molecular Simulation* **2013**, *39*, 1253-1292.
(25) Elder, A. C.; Bhattacharyya, S.; Nair, S.; Orlando, T. M. Reactive Adsorption of Humid SO2 on Metal–Organic Framework Nanosheets. *The Journal of Physical Chemistry C* **2018**, *122*, 10413-10422.
(26) Loiseau, T.; Serre, C.; Huguenard, C.; Fink, G.; Taulelle, F.; Henry, M.; Bataille, T.; Férey, G. A Rationale for the Large Breathing of the Porous Aluminum Terephthalate (MIL-53) Upon Hydration. *Chemistry – A European Journal* **2004**, *10*, 1373-1382.
(27) Petit, C.; Bandosz, T. J. Enhanced Adsorption of Ammonia on Metal-Organic Framework/Graphite Oxide Composites: Analysis of Surface Interactions. *Advanced Functional Materials* **2010**, *20*, 111-118.
(28) Valenzano, L.; Civalleri, B.; Chavan, S.; Bordiga, S.; Nilsen, M. H.; Jakobsen, S.; Lillerud, K. P.; Lamberti, C. Disclosing the Complex Structure of UiO-66 Metal Organic Framework: A Synergic Combination of Experiment and Theory. *Chemistry of Materials* **2011**, *23*, 1700-1718.
(29) Yu, D.; Wu, M.; Hu, Q.; Wang, L.; Lv, C.; Zhang, L. Iron-based metal-organic frameworks as novel platforms for catalytic ozonation of organic pollutant: Efficiency and mechanism. *Journal of Hazardous Materials* **2019**, *367*, 456-464.
(30) Tan, K.; Nijem, N.; Canepa, P.; Gong, Q.; Li, J.; Thonhauser, T.; Chabal, Y. J. Stability and Hydrolyzation of Metal Organic Frameworks with Paddle-Wheel SBUs upon Hydration. *Chemistry of Materials* **2012**, *24*, 3153-3167.
(31) George, P.; Das, R. K.; Chowdhury, P. Facile microwave synthesis of Ca-BDC metal organic framework for adsorption and controlled release of Curcumin. *Microporous and Mesoporous Materials* **2019**, *281*, 161-171.
(32) Hadjiivanov, K. I.; Panayotov, D. A.; Mihaylov, M. Y.; Ivanova, E. Z.; Chakarova, K. K.; Andonova, S. M.; Drenchev, N. L. Power of Infrared and Raman Spectroscopies to Characterize Metal-Organic Frameworks and Investigate Their Interaction with Guest Molecules. *Chemical Reviews* **2021**, *121*, 1286-1424.
(33) Deacon, G. B.; Phillips, R. J. Relationships between the carbon-oxygen stretching frequencies of carboxylato complexes and the type of carboxylate coordination. *Coordination Chemistry Reviews* **1980**, *33*, 227-250.
(34) Tan, K.; Zuluaga, S.; Gong, Q.; Canepa, P.; Wang, H.; Li, J.; Chabal, Y. J.; Thonhauser, T. Water Reaction Mechanism in Metal Organic Frameworks with Coordinatively Unsaturated Metal Ions: MOF-74. *Chemistry of Materials* **2014**, *26*, 6886-6895.
(35) Colthup, N. B.; Daly, L. H.; Wiberley, S. E. *Introduction to Infrared and Raman Spectroscopy, 3rd edition.* **1990**, *Academic Press, San Diego, California*.
